# Radar for Europa Assessment and Sounding: Ocean to Near-Surface (REASON)

**DOI:** 10.1007/s11214-024-01072-3

**Published:** 2024-06-27

**Authors:** Donald D. Blankenship, Alina Moussessian, Elaine Chapin, Duncan A. Young, G. Wesley Patterson, Jeffrey J. Plaut, Adam P. Freedman, Dustin M. Schroeder, Cyril Grima, Gregor Steinbrügge, Krista M. Soderlund, Trina Ray, Thomas G. Richter, Laura Jones-Wilson, Natalie S. Wolfenbarger, Kirk M. Scanlan, Christopher Gerekos, Kristian Chan, Ilgin Seker, Mark S. Haynes, Amy C. Barr Mlinar, Lorenzo Bruzzone, Bruce A. Campbell, Lynn M. Carter, Charles Elachi, Yonggyu Gim, Alain Hérique, Hauke Hussmann, Wlodek Kofman, William S. Kurth, Marco Mastrogiuseppe, William B. McKinnon, Jeffrey M. Moore, Francis Nimmo, Carol Paty, Dirk Plettemeier, Britney E. Schmidt, Mikhail Y. Zolotov, Paul M. Schenk, Simon Collins, Harry Figueroa, Mark Fischman, Eric Tardiff, Andy Berkun, Mimi Paller, James P. Hoffman, Andy Kurum, Gregory A. Sadowy, Kevin B. Wheeler, Emmanuel Decrossas, Yasser Hussein, Curtis Jin, Frank Boldissar, Neil Chamberlain, Brenda Hernandez, Elham Maghsoudi, Jonathan Mihaly, Shana Worel, Vik Singh, Kyung Pak, Jordan Tanabe, Robert Johnson, Mohammad Ashtijou, Tafesse Alemu, Michael Burke, Brian Custodero, Michael C. Tope, David Hawkins, Kim Aaron, Gregory T. Delory, Paul S. Turin, Donald L. Kirchner, Karthik Srinivasan, Julie Xie, Brad Ortloff, Ian Tan, Tim Noh, Duane Clark, Vu Duong, Shivani Joshi, Jeng Lee, Elvis Merida, Ruzbeh Akbar, Xueyang Duan, Ines Fenni, Mauricio Sanchez-Barbetty, Chaitali Parashare, Duane C. Howard, Julie Newman, Marvin G. Cruz, Neil J. Barabas, Ahmadreza Amirahmadi, Brendon Palmer, Rohit S. Gawande, Grace Milroy, Rick Roberti, Frank E. Leader, Richard D. West, Jan Martin, Vijay Venkatesh, Virgil Adumitroaie, Christine Rains, Cuong Quach, Jordi E. Turner, Colleen M. O’Shea, Scott D. Kempf, Gregory Ng, Dillon P. Buhl, Timothy J. Urban

**Affiliations:** 1 Institute for Geophysics, University of Texas at Austin, Austin, TX 78758 USA; 2 Jet Propulsion Laboratory, California Institute of Technology, Pasadena, CA 91109 USA; 3 Applied Physics Laboratory, Johns Hopkins University, Laurel, MD 20723 USA; 4 Department of Electrical Engineering, Stanford University, Stanford, CA 94305 USA; 5 Department of Geophysics, Stanford University, Stanford, CA 94305 USA; 6 Geodesy & Earth Observation Division, DTU Space, Technical University of Denmark, 2800 Kongens Lyngby, Denmark; 7 Department of Earth and Planetary Sciences, Jackson School of Geosciences, University of Texas at Austin, Austin, TX 78712 USA; 8 Planetary Science Institute, Tucson, AZ 85719 USA; 9 University of Trento, Trento, Italy; 10 Smithsonian Institution, Center for Earth & Planetary Studies, MRC 315, Washington, DC 20013-7012 USA; 11 Lunar and Planetary Laboratory, University of Arizona, Tucson, AZ 85721 USA; 12 California Institute of Technology, Pasadena, CA 91125 USA; 13 University Grenoble Alpes, CNRS, CNES, IPAG, 38000 Grenoble, France; 14 Institute of Planetary Research, German Aerospace Center, Berlin, Germany; 15 Centrum Badan Kosmicznych Polskiej Akademii Nauk (CBK PAN), Warsaw, Poland; 16 Department of Physics and Astronomy, University of Iowa, Iowa City, IA 52242 USA; 17 University of Rome “La Sapienza”, Rome, Italy; 18 Washington University in St. Louis, St. Louis, MO 63130 USA; 19 NASA Ames Research Center, Moffett Field, CA 94035 USA; 20 Dept. Earth and Planetary Sciences, University of California, Santa Cruz, CA 95064 USA; 21 Department of Earth Sciences, University of Oregon, Eugene, OR 97403 USA; 22 Technische Universität Dresden, 01069 Dresden, Germany; 23 Department of Earth and Atmospheric Sciences, Cornell University, Ithaca, NY USA; 24 Department of Astronomy, Cornell University, Ithaca, NY USA; 25 School of Earth and Space Exploration, Arizona State University, Tempe, AZ 85287 USA; 26 Lunar and Planetary Institute, Houston, TX 77058 USA; 27 Kinemetrics Inc., Pasadena, CA 91107 USA; 28 Planet, San Francisco, CA 94107 USA; 29 Laboratory for Atmospheric and Space Physics, University of Colorado, Boulder, CO 80303 USA; 30 Heliospace Corporation, Berkeley, CA 94710 USA; 31 Tomorrow.io, Boston, MA 02210 USA; 32 Center for Quantum Computing, Amazon Web Services, Pasadena, CA 91125 USA; 33 The Boeing Company, El Segundo, CA 90245 USA; 34 B&R Electronics, Reno, NV 89500 USA

**Keywords:** Europa, Ice shell, Ice-penetrating radar, Europa Clipper

## Abstract

The Radar for Europa Assessment and Sounding: Ocean to Near-surface (REASON) is a dual-frequency ice-penetrating radar (9 and 60 MHz) onboard the Europa Clipper mission. REASON is designed to probe Europa from exosphere to subsurface ocean, contributing the third dimension to observations of this enigmatic world. The hypotheses REASON will test are that (1) the ice shell of Europa hosts liquid water, (2) the ice shell overlies an ocean and is subject to tidal flexing, and (3) the exosphere, near-surface, ice shell, and ocean participate in material exchange essential to the habitability of this moon. REASON will investigate processes governing this material exchange by characterizing the distribution of putative non-ice material (e.g., brines, salts) in the subsurface, searching for an ice–ocean interface, characterizing the ice shell’s global structure, and constraining the amplitude of Europa’s radial tidal deformations. REASON will accomplish these science objectives using a combination of radar measurement techniques including *altimetry*, *reflectometry*, *sounding*, *interferometry*, *plasma characterization*, and *ranging*. Building on a rich heritage from Earth, the moon, and Mars, REASON will be the first ice-penetrating radar to explore the outer solar system. Because these radars are untested for the icy worlds in the outer solar system, a novel approach to measurement quality assessment was developed to represent uncertainties in key properties of Europa that affect REASON performance and ensure robustness across a range of plausible parameters suggested for the icy moon. REASON will shed light on a never-before-seen dimension of Europa and – in concert with other instruments on Europa Clipper – help to investigate whether Europa is a habitable world.

## Introduction

### Europa Clipper Summary and Science Motivation for REASON

The icy landforms of Europa, some familiar and others enigmatic and unique in the Solar System, indicate a complex and recently (≲100 Myr) active history for its ice shell (Bierhaus et al. 2009; Doggett et al. 2009; Kattenhorn and Hurford 2009). The formation of these landforms, as well as the nature of the ice shell and character of the deep interior, have been driven by the analysis of data acquired during the Voyager 1 and 2 spacecraft encounters with Jupiter (1979) and the Galileo mission (1995–2003) (Alexander et al. 2009). These data include direct measurements in two dimensions of Europa’s surface (e.g., imaging and spectroscopy) and indirect measurements in a single dimension of Europa’s global subsurface properties (e.g., radio science and magnetometry).

In particular, analyses of these data have provided insights into the subsurface domains within and bounding Europa’s ice shell. Key among these insights was the presence of a global ocean hidden beneath the ice shell, inferred from the Galileo magnetometer data, (Carr et al. 1998; Khurana et al. 1998; Pappalardo et al. 1999; Kivelson et al. 2000) and a rocky mantle of unknown complexity, inferred from radio science data (Anderson et al. 1998). However, direct measurements of the near-surface (i.e., the upper few hundred meters) and subsurface of Europa’s ice shell, including its properties, structure, and bounding interfaces, are required to address the daunting framework of working hypotheses arising from interpretations of previous Voyager and Galileo mission data.

The key example of this are the many working hypotheses for the exchange of material between the surface and subsurface, and their implications for habitability,^1^ which made Europa a top candidate for exploration in both the 2003 and 2011 Planetary Decadal Surveys (National Research Council 2003, 2011). Assessing Europa’s potential for habitability requires understanding the structure and evolution of its ice shell as well as its coupling to the ocean/rocky mantle system and exosphere. The Radar for Europa Assessment and Sounding: Ocean to Near-surface (REASON) is designed to operate in conjunction with other investigations on Europa Clipper to accomplish this objective (see Pappalardo et al. 2024, this collection). REASON is an ice-penetrating radar^2^ optimized to probe Europa from its exosphere to its subsurface ocean. If successful, the REASON investigations will revolutionize our understanding of Europa’s ice shell by providing the first direct measurements of the structure and properties of these subsurface domains and their bounding interfaces at scales critical for understanding exchange processes that govern Europa’s potential for habitability.

REASON baseline science investigations of Europa require four types of measurement techniques: radar *altimetry* to determine surface topography, radar *reflectometry* to study surface roughness and near-surface^3^ structure and composition as well as radar *sounding* to probe both the shallow and full depth subsurface domains of the ice shell coupled with radar *interferometry* to discriminate nadir subsurface features from radar returns of cross-track surface features. Two additional measurement techniques, radar *plasma characterization* (to derive the total electron content (TEC) in the ionosphere) and radar *ranging*^4^ (to measure the differential range between the spacecraft and the surface through time at groundtrack crossover points) provide, respectively, a critical framework for understanding the boundary conditions for surface/near-surface exchange and the energetics of tidal deformation across the ice shell’s full breadth and depth.

### Radar Sounding (and Assessment) of Ice from the Inner to the Outer Solar System

Radar sounding refers to a technique where electromagnetic energy is directed and transmitted into the subsurface, producing reflections which are recorded as signals. This is distinct from side-looking imaging radars, where energy is directed and transmitted off-nadir and backscattered energy is recorded as signal. Consequently, smooth topographic features that are highly reflective and specular are “bright” (i.e., high signal-to-noise ratio, SNR) in radar sounding data and “dark” (i.e., low SNR) in imaging radar data. Ice penetrating radars have the unique capability to penetrate deep into the subsurface of icy bodies due to the high transparency of ice at MHz frequencies (Warren 1984; Blankenship et al. 2009; Kofman et al. 2010; Pettinelli et al. 2015). As these transmitted radio waves travel through the subsurface, their reflections, referred to as “returns” or “echoes”, vary in power and character as they interact with subsurface horizons and structures that are associated with dielectric contrasts (i.e., changes in their relative dielectric permittivity^5^). A critical consideration for radar studies of icy worlds across the solar system is the leverage provided by the thermophysical transformation of ice to water, which is characterized by a contrast in permittivity of over an order of magnitude.

From its early origins measuring the thickness of glaciers and the extent of floating ice shelves on Earth, ice penetrating radar has proven to be a versatile geophysical technique used to constrain the presence of sub-ice water (e.g., water channels, lakes, ocean incursion), properties of the entire ice column (e.g., porosity, thermal state, composition), basal conditions (e.g., frozen vs. thawed, floating vs grounded ice, topographic heterogeneity), and ice dynamics (e.g., variations in accumulation/melting and flow/fracture) (Schroeder et al. 2020). Application of radar sounding as a ground based and airborne geophysical technique has been extended to orbital studies of bodies beyond Earth, with extensive coverage of the Moon (Porcello et al. 1974) and Mars (Picardi et al. 2004; Seu et al. 2007; Jordan et al. 2009). Multiple mission concepts to study the icy Galilean moons of Jupiter have highlighted subsurface sounding of the ice as a necessary technique for accomplishing proposed science goals (Greely and Johnson 2004) or explicitly included ice-penetrating radar as part of the model payload (Ludwinski et al. 1998; Clark et al. 2009; Lewis et al. 2016). For Europa in particular, early feasibility studies supported radar sounding with an ice penetrating radar as capable of characterizing the three-dimensional distribution of subsurface water, including the potential detection of a subsurface ocean (Chyba et al. 1998; Blankenship et al. 1999). Ultimately, these early mission concepts evolved into the National Aeronautics and Space Administration (NASA) Europa Clipper mission (Brown 2021) and the European Space Agency (ESA) JUpiter ICy moons Explorer (JUICE) mission (Grasset et al. 2013), both of which include ice penetrating radars. The radar data collected in the Jovian system by REASON on Europa Clipper and by the Radar for Icy Moons Exploration (RIME) on JUICE (Bruzzone et al. 2013) will mark the ultimate extension of our capacity for radar sounding and assessment of ice from the inner to the outer Solar System.

### Earth’s Cryosphere

Radar sounding and assessment of Earth’s cryosphere represents the foundation from which planetary radioglaciology has been and will continue to be built. As such, terrestrial data sets, analysis techniques, and confounding factors represent critical historical context for interpreting future radar sounding data collected on other worlds.

**Fig. 1:**
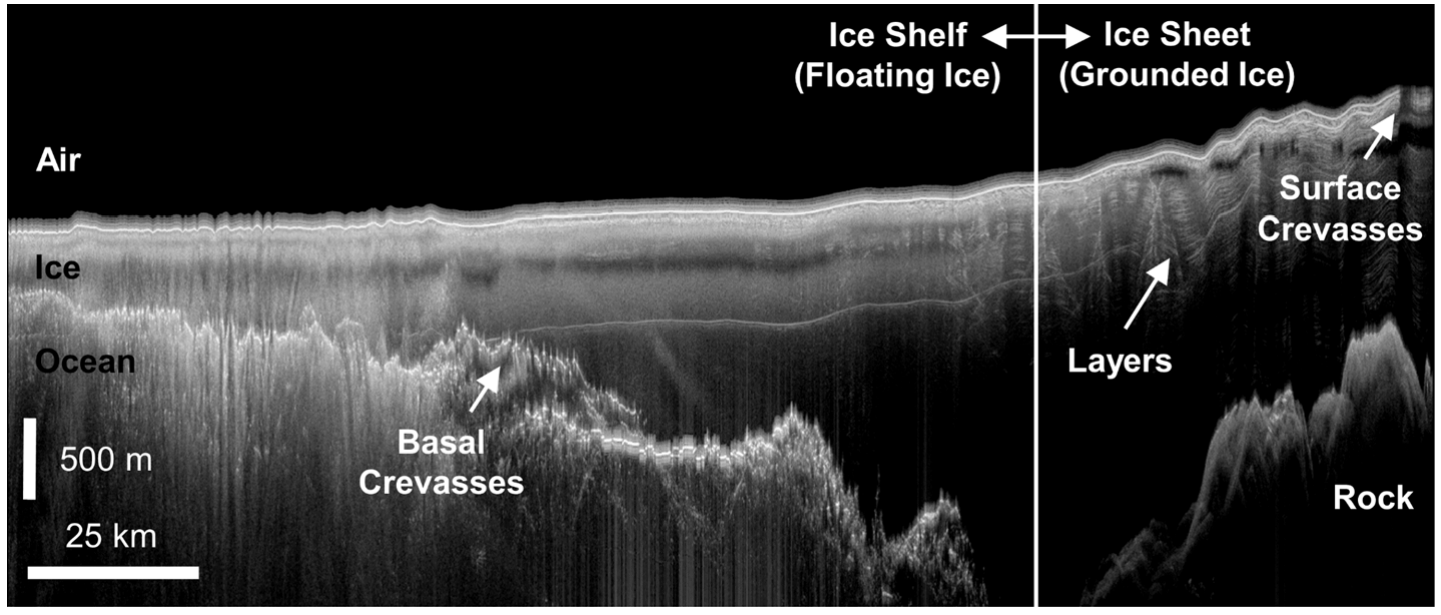
Radargram generated from ice-penetrating radar data collected over Totten Ice Shelf, Antarctica (TOT/JKB2d/X16a) modified from Lindzey (2015)

Radar surveys of the terrestrial cryosphere, both floating and grounded (Fig. 1), were originally pursued to generate maps of ice thickness and surface/basal topography (Bailey et al. 1964; Gudmandsen 1969) – an effort that continues to date (Lythe and Vaughan 2001; Fretwell et al. 2013; Frémand et al. 2023). Surveys in recent decades have expanded their scope from identification to characterization of the bed (Schroeder et al. 2020 and references therein), where bed characteristics are typically quantified using the power of the basal return (Peters et al. 2005b; Christianson et al. 2016; Chu et al. 2021). Radar sounding data are often visualized using radargrams (Fig. 1), which display the power of the return as a function of travel time (fast time) on the vertical axis and along-track distance (slow time) on the horizontal axis.

**Fig. 2:**
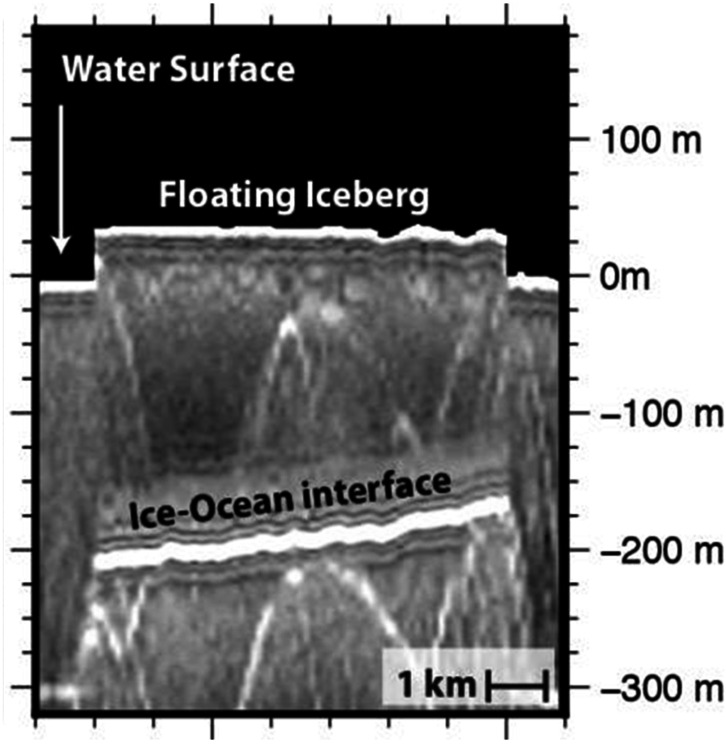
Radargram generated from ice-penetrating radar data collected over iceberg B15, Antarctica (MCM/SJB2/BERG04c; Blankenship, personal communication)

On Earth, radar altimetry profiles collected over ice have been an essential foundation for understanding the evolution of our glaciers, ice sheets, and ice shelves (Rémy and Parouty 2009; Fricker and Padman 2012). These profiles, obtained by identifying the surface echo and the associated delay, have long been used to quantify the basal stress regime for grounded ice, which is a function of ice surface slope (Cooper et al. 1982; Bentley 1987). Knowledge of the state of stress provides essential context for discriminating geologic features within the ice and the hypothesized processes responsible for their formation (e.g., Blankenship et al. 2001). For floating ice, the detection of liquid water can be confirmed by comparing the projected ice thickness, derived from surface elevations measured by altimetry, to the measured ice thickness, obtained directly by sounding (Jenkins and Doake 1991; Fricker et al. 2001). An example of particular relevance to Europa are floating icebergs (Peters et al. 2007b). As illustrated in Fig. 2, floating icebergs will protrude above the surface by ∼10% of the total ice thickness due to the ∼10% difference in densities of water and ice (∼1000 and 920 kg/m^3^, respectively).

**Fig. 3:**
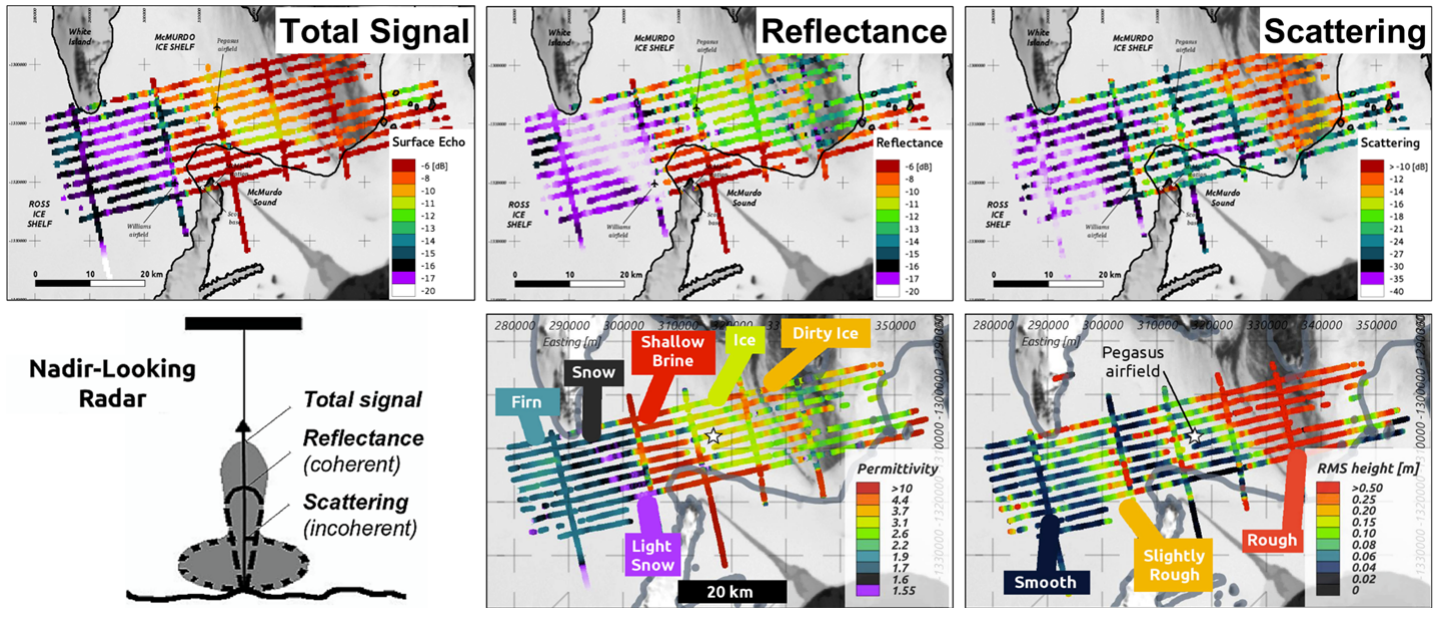
Near-surface properties of McMurdo Ice Shelf, Antarctica derived from ice-penetrating radar data, modified from Grima et al. (2016)

In addition, assessment of near-surface properties has become increasingly important for Earth’s cryospheric systems as the thermal/mass exchange between the ice and our atmosphere is increasingly perturbed (Humphrey et al. 2012; Machguth et al. 2016; Mortimer et al. 2016). The embodiment and implications of these changes can contrast substantially with the grounded and floating ice systems of Earth providing a broad diversity of constraints on ice accumulation and melting for its ice sheets and ice shelves. To understand these complex and heterogeneous ice/water systems, statistical analyses of surface echo amplitude have been used to separate the reflected and scattered components of radar signals of multiple frequencies to simultaneously obtain surface roughness and bulk permittivity of the near-surface for both floating and grounded ice systems at multiple scales (Fig. 3) (Grima et al. 2014a,b, 2016, 2017, 2019). It has also been possible to combine these multiple frequency approaches with a careful assessment of frequency segments across the band of a particular radar to constrain both the lateral and vertical heterogeneity of these bulk near-surface properties (Chan et al. 2023b).

**Fig. 4:**
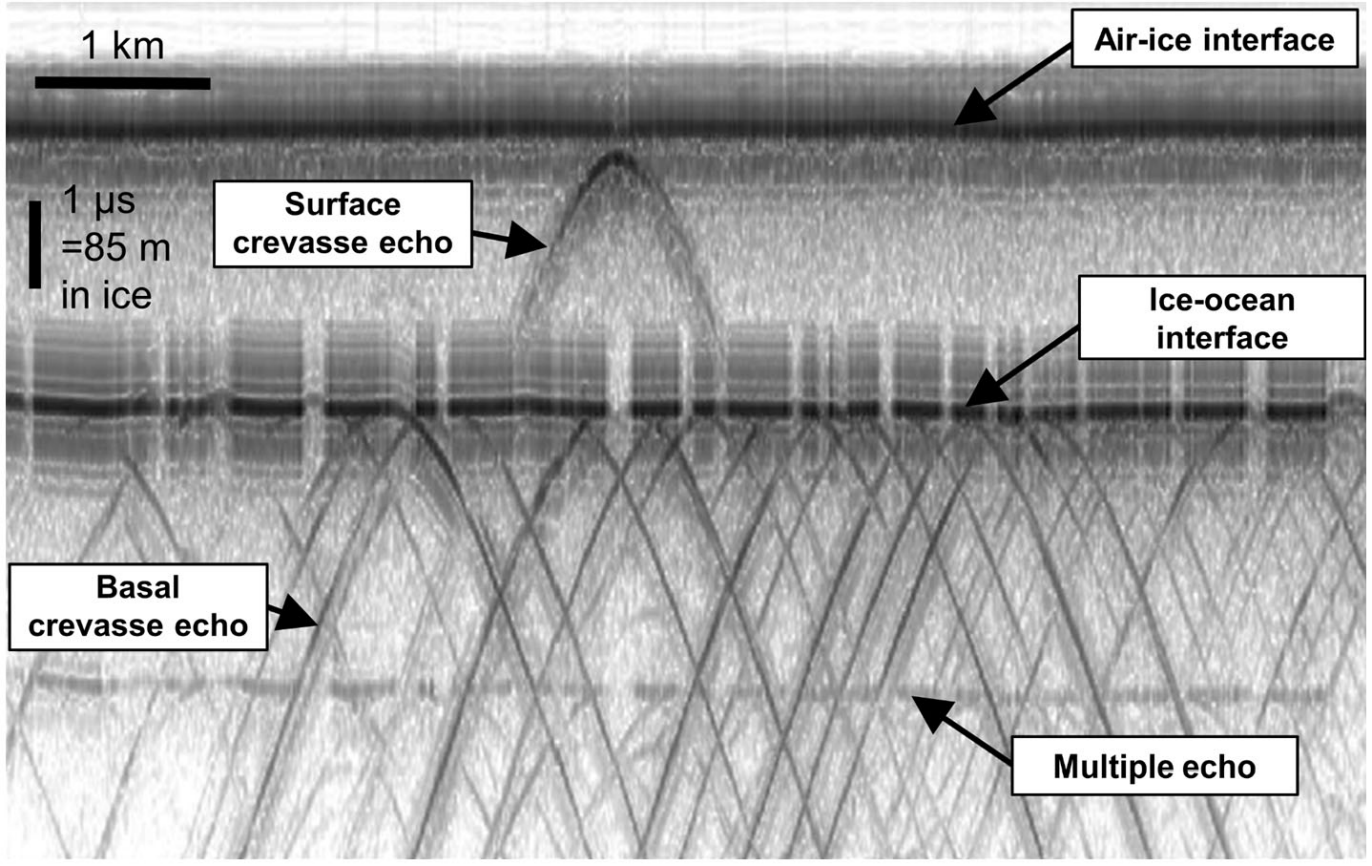
Radargram generated from ice-penetrating radar data collected over iceberg B15, Antarctica showing extensive surface and basal crevassing, modified from Peters et al. (2007b)

A great deal of the early airborne radar assessment and sounding of Earth’s cryosphere was focused on its ice shelves (Fig. 1) (Vaughan et al. 1995). It was hypothesized over a half century ago that instabilities in these ice shelves would be the primary trigger for rapid draining of the substantial interior grounded ice reservoir for large segments of the Antarctic ice sheet, causing sea level rise (Weertman 1974; Mercer 1978). Mapping of both surface and basal crevassing (Fig. 4) was an important element of this work with parallel studies of ice shelf thickness. These analyses, which initially focused on qualitatively identifying regions of clutter (Jezek et al. 1979; Jezek and Bentley 1983; Jezek 1984), have recently evolved to more quantitative assessments of crevassing processes, including the discrimination of water versus ice filled basal crevassing (Peters et al. 2007b). We anticipate that the arc of radar studies of the icy worlds of the outer planets will follow a similar pattern in the shallow subsurface, where brittle fracture might dominate.

**Fig. 5:**
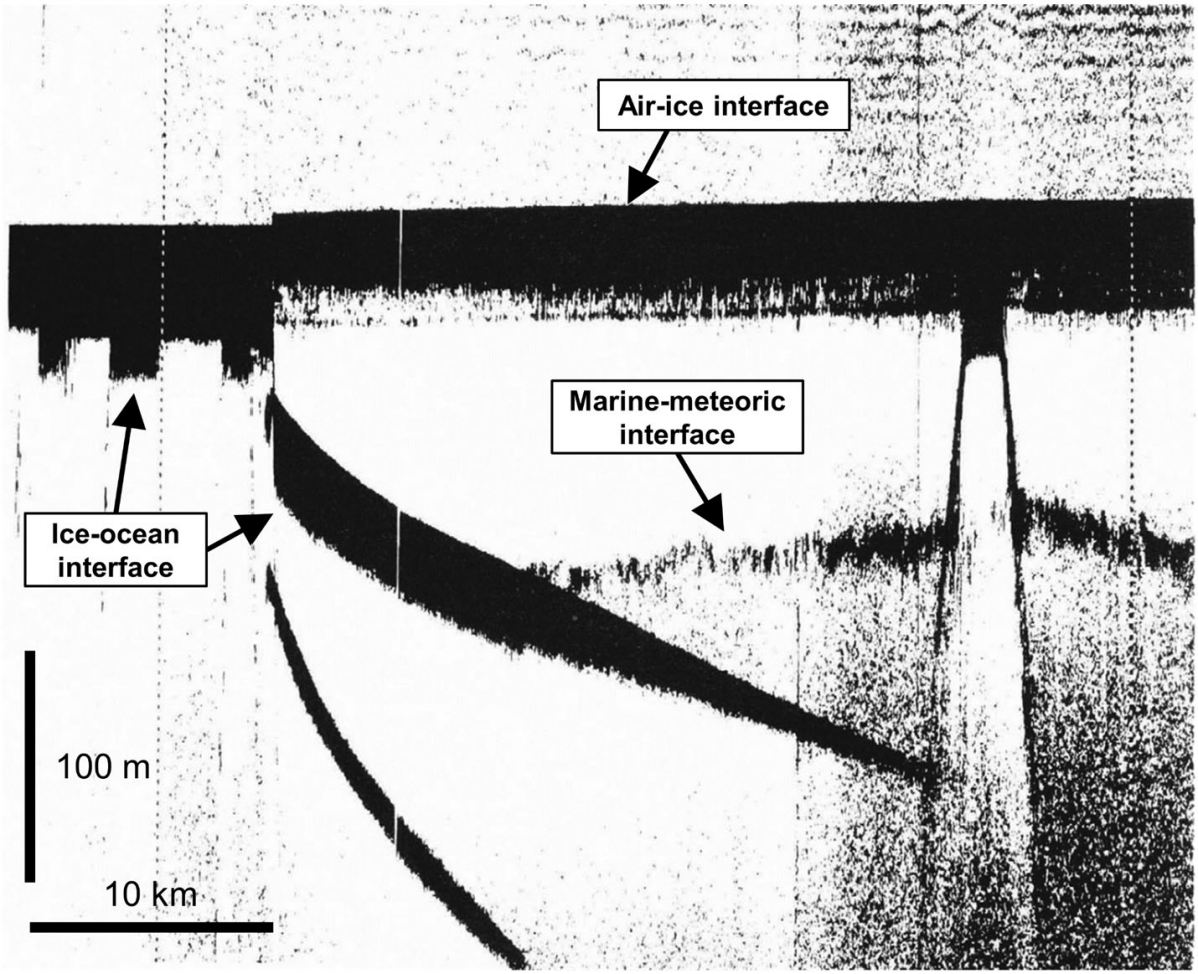
Radargram generated from ice-penetrating radar data collected over Filchner-Ronne Ice Shelf, Antarctica illustrating how a marine–meteoric ice interface could be mistaken as the ice–ocean interface, modified from Thyssen (1988)

An offshoot of the early radar sounding of Earth’s ice shelves, perhaps even more relevant to icy ocean worlds, was the misinterpretation of the sub-ice ocean interface from radar sounding over Filchner-Ronne Ice Shelf (Robin et al. 1983; Crabtree and Doake 1986). When more precise ice surface altimetry became available, it was discovered that the “measured” thickness from radar sounding could not be reconciled with the new measurements of surface elevation using Archimedes’ principle (Thyssen 1988). Subsequent drilling showed unequivocally that the basal feature that was interpreted as the ice–ocean interface was instead a boundary between the glacier-derived floating “meteoric ice” and saltier “marine ice” frozen from the ocean below (Engelhardt and Determann 1987; Oerter et al. 1992), as illustrated in Fig. 5. From that point on the radar altimetry and sounding observations were combined to establish and map the thickness of the underplated marine ice to test various working hypotheses for the distribution of melting and freezing associated with sub-ice ocean circulation (Fricker et al. 2001; Joughin and Vaughan 2004; Lambrecht et al. 2007).

**Fig. 6:**
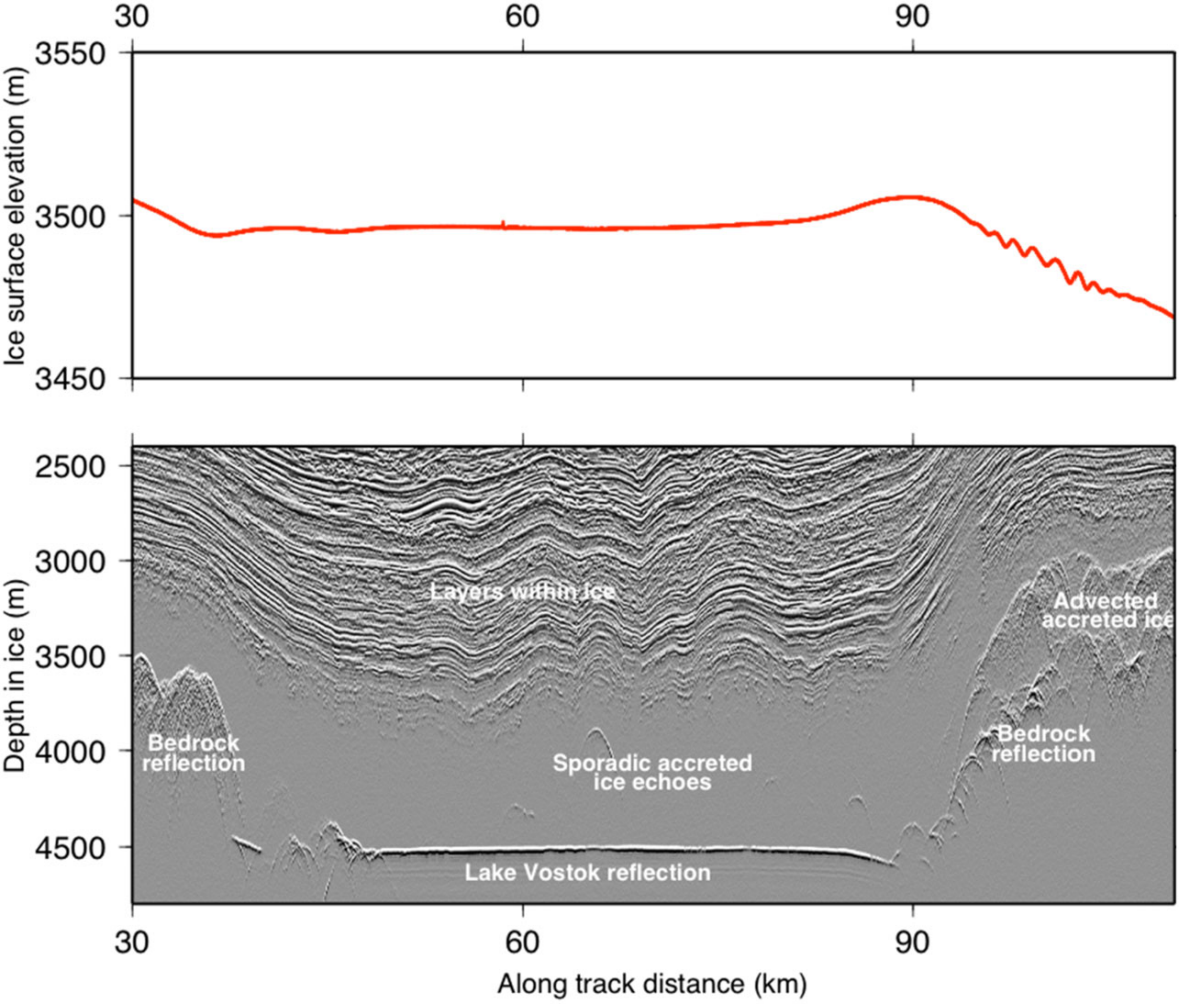
Altimetry profile (top) and radargram (bottom) generated from ice-penetrating radar and laser altimetry data collected over Lake Vostok, Antarctica illustrating how complementary altimetry and radar sounding data can support the identification of subglacial lakes, modified from Blankenship et al. (2009)

For grounded ice sheets on Earth, radar sounding has also led to the discovery of a vast inventory of subglacial lakes in both the Arctic and Antarctic (Livingstone et al. 2022). Traditionally, this is accomplished by detecting regions that appear “brighter” than the surrounding terrain (Carter et al. 2007; Ilisei et al. 2019). Again, surface altimetry serves as an essential complementary data set to identify these subglacial lakes (Fig. 6). Ice thickness estimates coupled with surface elevation measurements enable hydrostatic analysis to determine whether ice over putative lakes is in hydrostatic equilibrium (Vaughan et al. 2007; Ewert et al. 2012). Variations in ice surface topography through time can indicate drainage and recharge of active subglacial lakes (Wingham et al. 2006; Siegfried and Fricker 2021). Confounding interpretation, some subglacial lakes identified through altimetry do not appear radar-bright (Carter et al. 2007; Humbert et al. 2018; Lindzey et al. 2020). Ultimately these anomalous subglacial lakes can only be unambiguously confirmed through subglacial drilling (Talalay 2012; Tulaczyk et al. 2014; Priscu et al. 2021) or seismic surveys (Peters et al. 2008; Woodward et al. 2010; Horgan et al. 2012).

The pace of radar studies of Earth’s cryosphere is increasing rapidly, not only because of the threat of its role in rapid sea level rise on Earth, but also because of the recognition of the importance of potential ice-covered habitats hosted by both rock and water systems on Earth for understanding habitability across the solar system. Great advances have recently been made in quantifying radar reflectivity and scattering in pursuit of understanding exchange processes for the surface/near-surface and full depth/water interface studies on Earth. Reflectivity has often been considered synonymous with the Fresnel reflection coefficient, governed by the contrast in dielectric properties across an interface (Ulaby and Long 2015; Christianson et al. 2016). However, the apparent reflectivity is also influenced by scattering associated with the wavelength scale roughness of the surface and/or interface (Peters et al. 2005b; Campbell 2009; Rippin et al. 2014) and properties of the subsurface ice, including the presence of volume scatterers (Chu et al. 2018; Culberg and Schroeder 2020). Another factor influencing reflectivity is the electrical conductivity, which is governed by the thermal profile within the ice column as well as the concentration of lattice soluble impurities (Fujita et al. 2000; Moore 2000; MacGregor et al. 2007; Matsuoka et al. 2012; MacGregor et al. 2015). Uncertainty in these factors challenges interpretation of radar data (Matsuoka 2011). Methods have been developed to deconvolve these factors, which include estimating and correcting attenuation through the ice column (Ashmore et al. 2014; Hills et al. 2020), leveraging the statistics of echo power over a region (Grima et al. 2014a), or examining the properties of the return signal itself (Schroeder et al. 2014; Jordan et al. 2017). These novel analysis techniques have enabled the mapping of ice shelf brines (Grima et al. 2016), improved identification of frozen vs. thawed beds (Jordan et al. 2018; Franke et al. 2021), and, most notably, the discovery of new subglacial aqueous systems as candidates for habitable environments (Schroeder et al. 2013; Rutishauser et al. 2018, 2022).

### From the Moon to Mars and Back

One of the earliest applications of radar sounding beyond Earth was the Apollo 17 Lunar Sounder Experiment (ALSE), a multi-frequency imaging radar (5, 15, 150 MHz or 60, 20, 2 m) with a subsurface sounding requirement (Porcello et al. 1974). The longer wavelengths enabled deeper penetration into the lunar regolith, whereas the shorter wavelengths enabled higher quality surface images and profiles. The primary science objective was the detection of subsurface discontinuities (sounding) and three secondary science objectives were surface imaging, surface profiling, and radio astronomy (i.e., measurement of galactic noise) (Phillips et al. 1973; Porcello et al. 1974). Using the 5 MHz band, the ALSE produced elevation profiles of the Moon (Brown et al. 1974) and ultimately identified two reflectors beneath Mare Serenitatis at depths of 0.9 km and 1.6 km and one reflector beneath Mare Crisium at a depth of 1.4 km (Peeples et al. 1978).

After almost forty years, the ALSE was followed by the Mars Advanced Radar for Subsurface and Ionospheric Sounding (MARSIS) instrument flown on ESA’s Mars Express mission (Picardi et al. 2004; Jordan et al. 2009). MARSIS is a dual-channel radar sounder operating between frequencies of 1.3 and 5.5 MHz for subsurface sounding and 0.1 and 5.5 MHz for ionospheric sounding. The primary science objectives were to map the subsurface distribution of water ice and liquid water, probe subsurface geology, characterize the surface, and sound the ionosphere. The principal results of the first decade of observation by MARSIS are summarized in Orosei et al. (2015), including confirming the hypothesis of a predominantly icy composition for the Polar Layered Deposits (Plaut et al. 2007). Recent analyses of MARSIS data collected over the South Polar Layered Deposits (SPLD) suggest the presence of subglacial hypersaline bodies of water (Orosei et al. 2018; Lauro et al. 2021, 2022); however, this interpretation has been the subject of intense debate (Sect. 9.1.2). MARSIS was closely followed by the SHAllow RADar (SHARAD), a radar sounder flown on NASA’s Mars Reconnaissance Orbiter (MRO) designed to detect shallow subsurface interfaces (Seu et al. 2004). SHARAD operates at a center frequency of 20 MHz and with a bandwidth of 10 MHz. Major scientific findings derived from the first nine years of SHARAD are summarized in Croci et al. (2011), including the discovery that many mid-latitude lobate features are ice-rich, debris-covered glaciers (Holt et al. 2008). At the poles, SHARAD data revealed a highly complex and temporally-varying pattern of deposition and ablation/sublimation (Putzig et al. 2009; Smith et al. 2016; Campbell and Morgan 2018), and made the revolutionary discovery of CO_2_ ice deposits at the south pole trapped beneath a surface lag and capable of doubling the current atmospheric pressure if released (Phillips et al. 2011).

Following MARSIS and SHARAD, Mars became the most globally well-characterized body by ground-penetrating radar, and the target for new radar sounding data transitioned back to the Moon. The goals of the Lunar Radar Sounder (LRS) flown on JAXA’s SELenological and ENgineering Explorer (SELENE) were to map the subsurface structure of the Moon to 5 km depth with a vertical resolution of 100 m by operating between 4 and 6 MHz and to make passive observations of natural radio and plasma waves between 10 kHz and 30 MHz (Sasaki et al. 2003). Some key results from LRS are presented in Ono et al. (2010) and include the discovery of numerous subsurface reflectors several hundred meters deep in the nearside maria interpreted to be regolith interbedded with basaltic lava flows.

### Heritage from Existing Ice-Penetrating Radars

Technology has advanced significantly since the first radar soundings of glaciers (Stern 1930; Steenson 1951; Waite and Schmidt 1962; Turchetti et al. 2008). These early observations prompted the engineering of radar sounding systems dedicated to studying the cryosphere (Evans 1963; Gudmandsen 1969; Drewry 1983). However, these early systems were incoherent and thus limited to recording the radar signal amplitude. The development of terrestrial coherent ice-penetrating radars (Bentley et al. 1988; Raju et al. 1990; Chuah 1996; Gogineni et al. 1998; Moussessian et al. 2000; Peters et al. 2005b), which could record both the phase and amplitude, enabled improved along-track resolution and increased SNR using synthetic aperture radar (SAR) processing techniques (Peters et al. 2005b).

Unfocused SAR processing involves the coherent integration (summing) of the individual radar signals over an along-track distance (integration length) and is optimized when the integration length is equal to the Fresnel zone radius.^6^ Focused SAR processing leverages phase information to migrate reflected energy to its point of origin prior to coherent integration resulting in improved along-track resolution and processing gain relative to unfocused SAR processing (Legarsky et al. 2001). Focusing algorithms have been adapted and augmented to interrogate small-scale roughness and geometry of interfaces, permitting the characterization of subglacial water systems (Schroeder et al. 2014) and enhanced resolution of internal layers (Heister and Scheiber 2018; Castelletti et al. 2019; Ferro 2019).

**Fig. 7:**
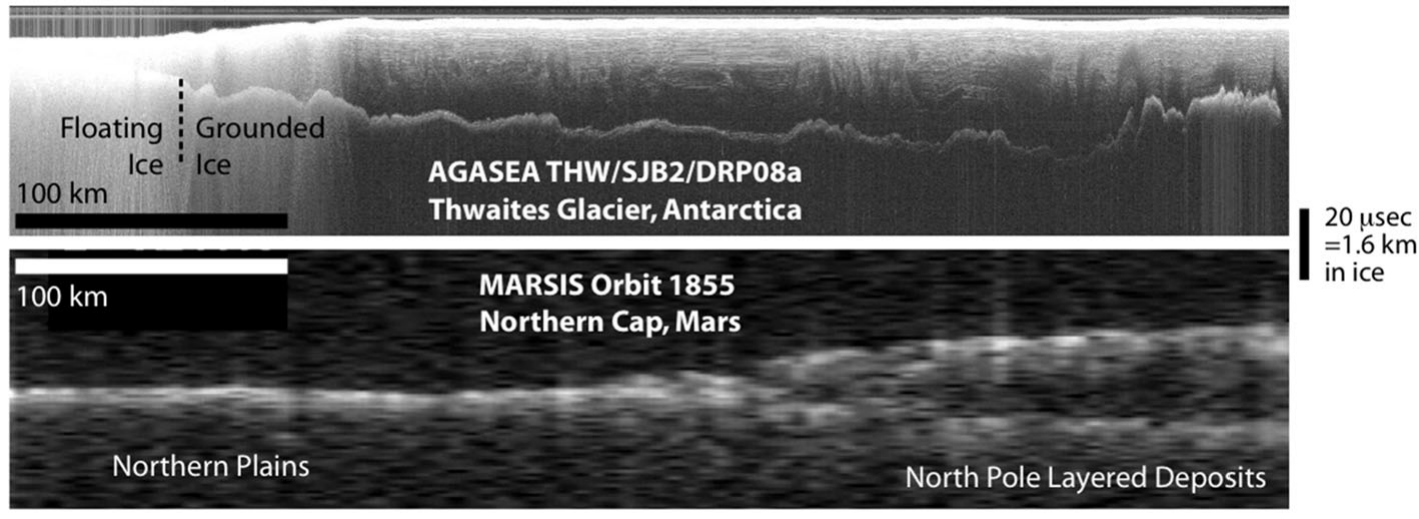
Comparison between radargrams generated from ice-penetrating radar data collected over ice on Earth at 60 MHz center frequency, 15 MHz bandwidth (top) and Mars at ∼1 MHz center frequency and bandwidth (bottom), modified from Picardi et al. (2005)

The coherent radar sounder developed by Moussessian et al. (2000) demonstrated the feasibility of collecting high altitude radar sounding data and served as a testbed for future spaceborne radar sounders. This sounder could operate at frequencies of 35, 50, 60, 75, and 150 MHz with a 15 MHz bandwidth. Initial field testing was done at 150 MHz with a University of Kansas antenna system (Chuah 1996). The system was subsequently tested at 60 MHz in Antarctic operations by the University of Texas Institute for Geophysics, the success of which led to the development of derivative designs used in polar aerogeophysics to the present day (Peters et al. 2005a). These radar sounders represent the predecessors for radar sounders flown on missions to Mars (Fig. 7) and ultimately Europa.

**Fig. 8:**
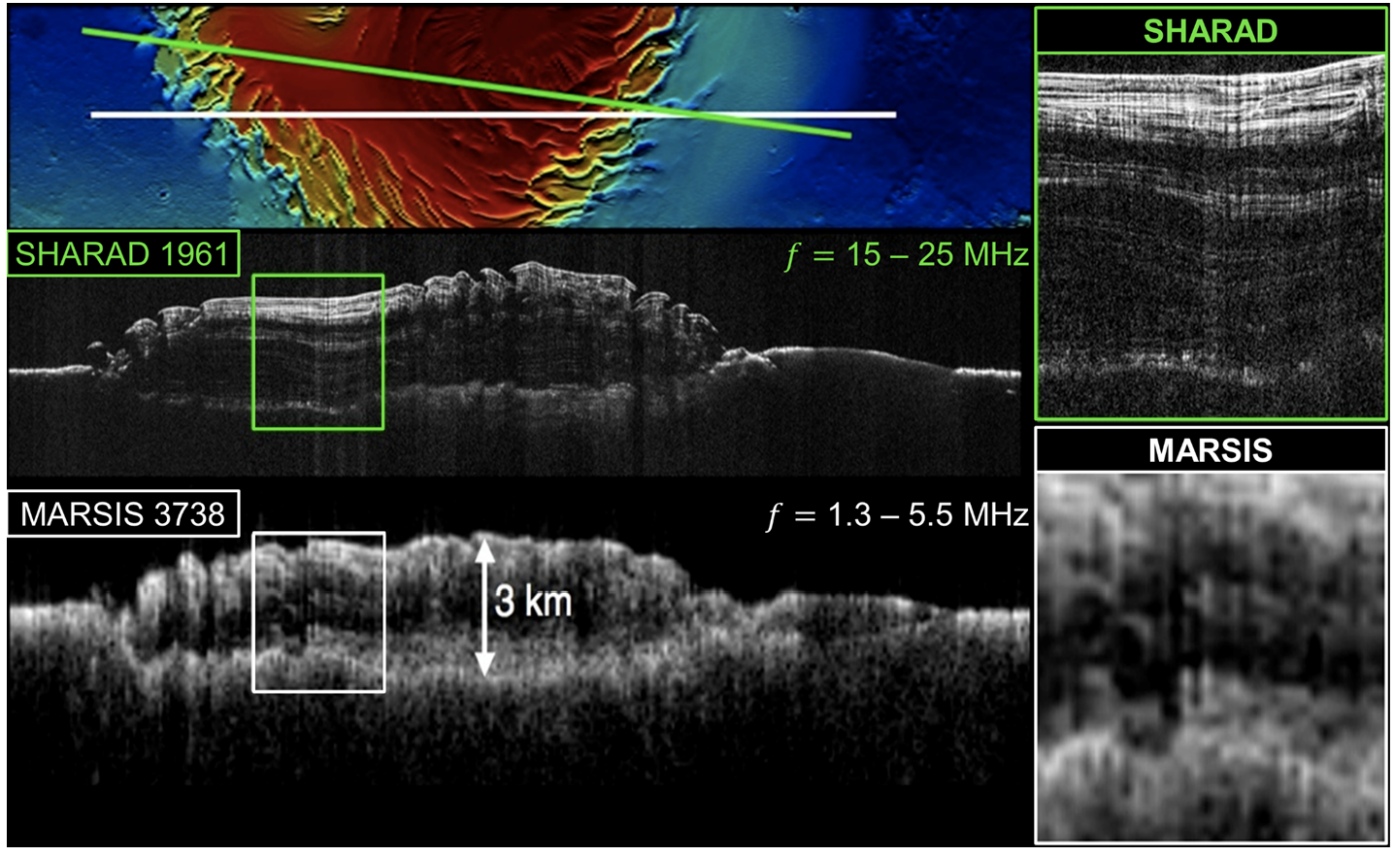
Comparison between radargrams generated from ice-penetrating radar data collected over the Mars North Polar Layered Deposits (top) by SHARAD (middle) and MARSIS (bottom), illustrating the scientific value of complementary sounding frequencies, modified from material provided by Ali Safaeinili

The complementary sounding frequencies of MARSIS (1.3 – 5.5 MHz) and SHARAD (15 – 25 MHz) at Mars demonstrated the scientific value of a dual-frequency approach to studying icy bodies (Seu et al. 2007; Jordan et al. 2009). The lower frequencies of MARSIS penetrated deeper within the ice column while the higher bandwidth of SHARAD enabled shallow internal layers to be resolved in greater detail (Fig. 8) (Croci et al. 2011). The use of two complementary frequencies represents a cornerstone of REASON’s approach to sounding Europa.

### REASON High-Level Instrument Description

REASON is a dual-frequency (9 MHz and 60 MHz) radar designed to penetrate deep into Europa’s ice crust, operating over a wide range of altitudes (35 km – 1000 km) across a flyby while simultaneously characterizing Europa’s surface/near-surface and exosphere. REASON uses a 1 MHz bandwidth at a 9 MHz (33.3 m wavelength) High Frequency (HF) center frequency and a 10 MHz bandwidth at a 60 MHz (5 m wavelength) Very High Frequency (VHF) center frequency to achieve low-resolution (300 m in ice) full-depth sounding and high-resolution (30 m in ice) shallow sounding simultaneously. The HF center frequency of 9 MHz was selected because it enables robust performance in areas of high surface roughness while avoiding prohibitive Faraday losses below 8 MHz. Although surface and volume scattering tend to decrease with increasing wavelength (i.e., VHF is more susceptible to scattering losses than HF), the HF band is highly susceptible to Jovian decametric noise. As such, HF is practically limited to the shielded anti-Jovian hemisphere of Europa or when Jovian noise is low. The unambiguous interpretation of subsurface interfaces from radargrams requires discriminating nadir subsurface signals from off-nadir surface returns, referred to as clutter. Along-track clutter will be discriminated by SAR processing, whereas across-track clutter will be discriminated using dual-channel VHF interferometry. REASON includes two VHF receive channels (separated by an across-track baseline) that will independently receive returns from both nadir subsurface sounding targets and off-nadir across-track surface clutter.

### REASON Measurement Techniques

**Fig. 9:**
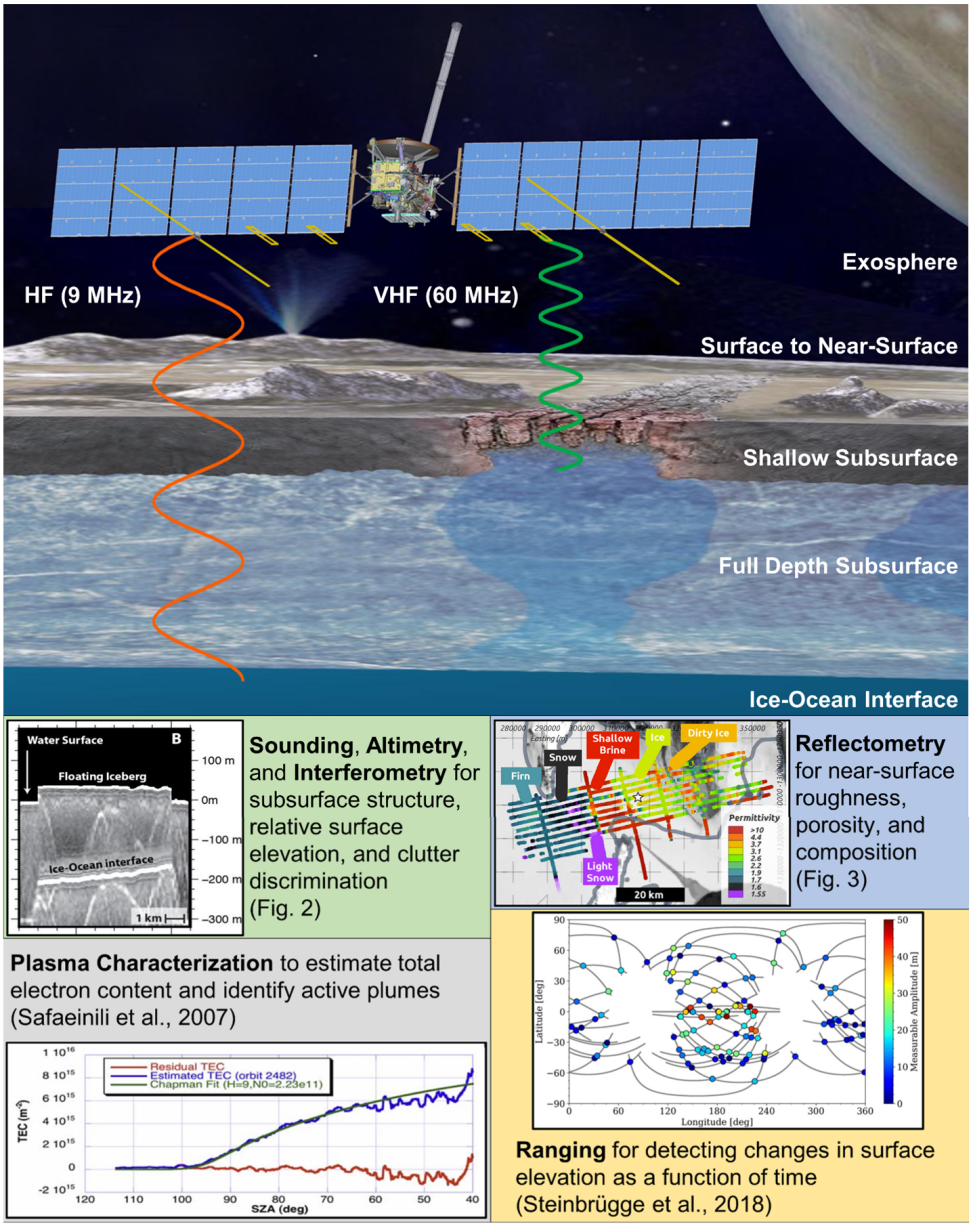
Summary of REASON ice shell domains and measurement techniques

REASON employs six measurement techniques: *altimetry*, *reflectometry*, *sounding, interferometry*, *plasma characterization*, and *ranging* (Fig. 9).

#### Altimetry (VHF)

Altimetry refers to measuring height profiles along-track by identifying the surface return. Due to spatial variations in attenuation within Europa’s ice shell, the observed strength of subsurface reflectors is an ambiguous indicator of the composition of the materials that define the interface (e.g., ice–water vs. ice–salt interface). As such, REASON’s *altimetry* measurement is required to discriminate hydrostatic reflectors, such as an ice–water interface from non-hydrostatic reflectors, such as an ice–salt interface. Subsurface water can be confirmed through hydrostatic analysis by comparing any inferred ice thickness, obtained using the measured surface elevation and an assumed density contrast between water and ice, to the measured ice thickness, obtained using surface and subsurface reflectors. This method has been integral to the identification of subglacial lakes under Earth’s ice sheets (Kapitsa et al. 1996; Carter et al. 2007). Similarly, this approach allows for non-water structures to be identified if the features do not have a hydrostatic relationship with the surface. It is the combination of *sounding* and *altimetry* that is required to characterize observed subsurface reflectors. REASON *altimetry* will be using a waveform fitting method derived from ocean altimetry to identify the surface return most likely originating from nadir. The method is based on the idea that from a rough surface the altimetric return can be described by a Brown model (Brown 1977). A subsequent parameter fit on the pulse compressed data then allows matching the surface return to the leading edge of the waveform. Despite being derived from ocean altimetry, the method has been applied with great success to SHARAD data on Mars (Steinbrügge et al. 2021).

#### Reflectometry (HF, VHF)

**Fig. 10:**
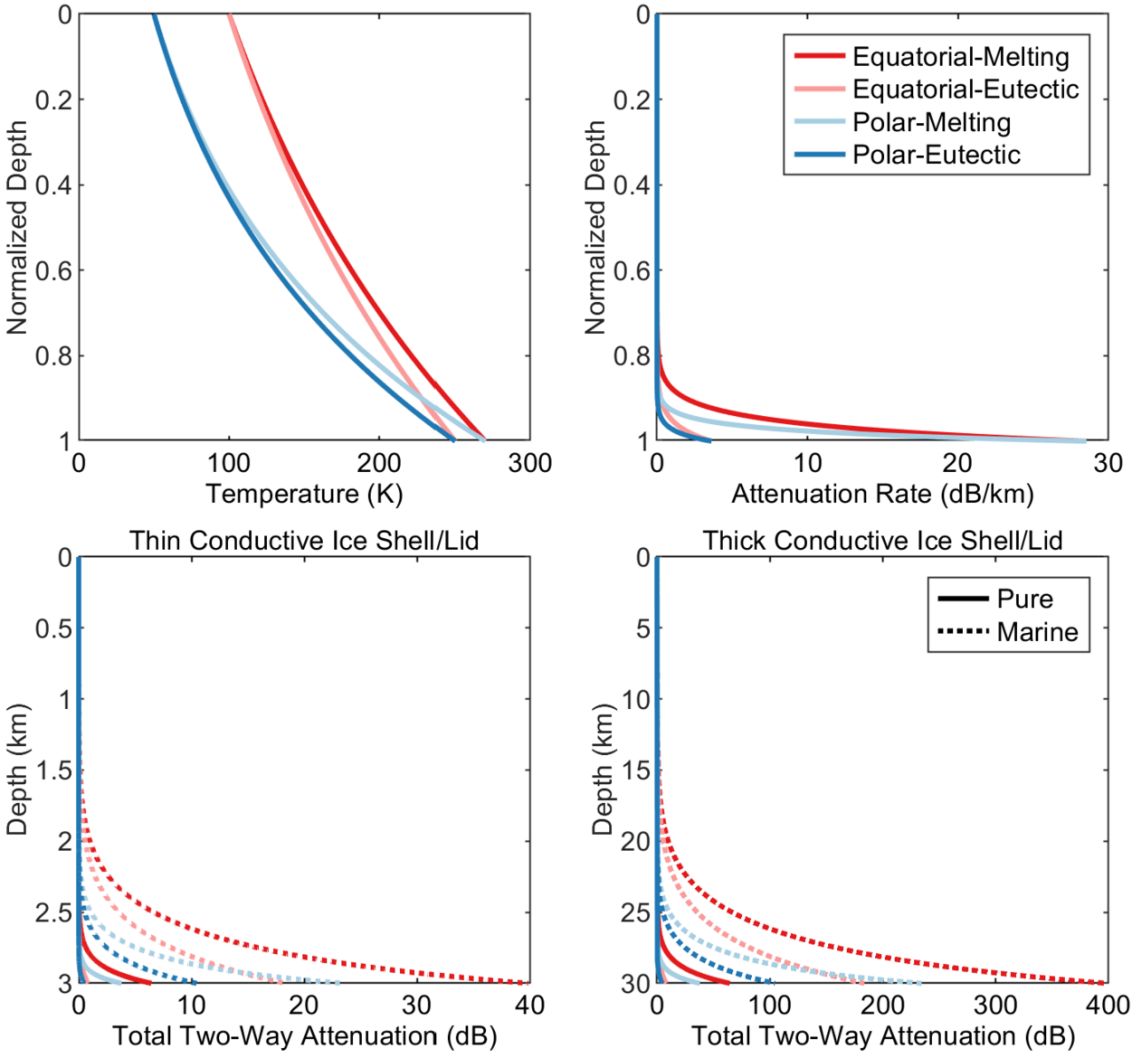
Modeled radar attenuation in Europa’s ice shell adopting the thermal model of Chyba et al. (1998), electrical parameters of Moore (2000), and the regime modes described in Table 4

The limited bandwidth (i.e., vertical resolution) of REASON limits direct imaging of the upper few hundred meters of the ice shell. As illustrated in Fig. 10, attenuation (i.e., electrical conductivity) should be very low in this cold, near-surface region, so all interfaces within the bandwidth-limited waveform should contribute to the echo. By comparing the distribution of the surface echo strengths from a resolution cell with a theoretical probability density function that accounts for stationary signal scattering, we can obtain the coherent and incoherent components of the surface echo for that cell (Grima et al. 2014b). Although these components are highly coupled, the coherent component is predominantly governed by the permittivity contrast at the surface and the deterministic structure of the near-surface, whereas the incoherent component is mainly controlled by scattering and surface roughness. The local geologic context and the observations (e.g., topography) acquired from other investigations can provide constraints or reasonable assumptions on some surface properties so that density or the presence of brine can be inferred (Grima et al. 2014a, 2016). Although brine intrusions would be transient and not thermodynamically stable in the near-surface of Europa (Vu et al. 2016; Thomas et al. 2017), their presence would generate echoes with a strongly coherent component. Such large anomalies in the coherent component of the backscattered signal may be a sign of recent brines within the near-surface (Grima et al. 2016; Haynes et al. 2018b), which would have significant implications for exchange processes.

#### Sounding (HF, VHF)

REASON’s *sounding* measurement is required to produce HF and shallow VHF radargrams up to depths of 30 km and 3 km, respectively. Full-depth VHF *sounding* extends to depths of 30 km, producing radargrams with higher vertical resolution (by a factor of ∼10) compared to the HF radargrams below 3 km. To reduce data volume, full-depth VHF sacrifices both radiometric fidelity (by reducing the bit depth) and *interferometry* (by combining channels). Simultaneous acquisition of HF and VHF allows us to correct for plasma effects through their different responses to charged particle density. By recording raw coherent radar echoes with phase intact and transmitting them to Earth, REASON has the capacity for SAR processing to achieve high along-track resolution through post-acquisition, ground-based processing. Requirements were defined assuming unfocused SAR processing, defined as the process of combining (coherently summing) the returns from individual pulses to increase the SNR. Due to the design of the Europa Clipper mission as a multiple flyby mission, the SAR focusing architecture must be tailored to REASON and will be distinct from previous orbital radar missions (Scanlan et al. 2021).

Note that although *sounding* measurements are required to produce radargrams capable of recording subsurface echoes from specific maximum depths, this does not imply that interfaces will necessarily be detected to these depths in practice. The depth to which a radargram can be produced is governed by the spacecraft altitude, length of the receive window, and data volume constraints, whereas the depth to which the HF and VHF signals can penetrate is governed by key parameters that describe the radar performance, observational parameters such as spacecraft altitude, and the unknown properties of Europa that can attenuate the transmitted signal.

Radar waves scatter from structures in the ice (e.g., voids), but the resulting losses are not expected to be significant obstacles to penetration (Aglyamov et al. 2017). In contrast, attenuation in warm ice with impurities could reduce penetration significantly (Fig. 10). An organizing principle of REASON’s approach to subsurface sounding is the concept of a nonlinear dependence of radar signal attenuation on temperature in a thermally conductive ice shell, overlying an ice–water interface, or a thermally conductive lid, overlying a convective ice layer (Fig. 10). Although the most reflective hypothesized interface on Europa will be that of a smooth ice–water interface, this interface will likely not be the brightest because of enhanced signal attenuation experienced as the interface is approached. Note that correlating variations in echo strength to variations in subsurface permittivity is challenging due to the possibility of local or regional heterogeneities in attenuation rate through the ice shell. *Sounding* will allow us to search for subsurface interfaces but to characterize the resultant echoes will require additional information provided by *altimetry*.

#### Interferometry (VHF)

Interferometry is a radar technique which involves comparing the phase of echoes recorded by separate receivers to establish whether the echo is from a subsurface nadir target or an off-nadir cross-track surface/subsurface feature (Castelletti et al. 2017; Haynes et al. 2018a; Scanlan et al. 2020). We exploit the fact that the VHF radar consists of several receivers separated by a baseline. When recording reflected signals, the four element VHF array is divided into two cross-track receive channels (one for each pair of VHF antennas on either side of the spacecraft). Because the path lengths traveled by radar waves reflected from flat surface and subsurface targets at nadir are equivalent between the two cross-track antenna pairs, the measured phase difference (interferometric phase) between the echoes will be generally close to zero. However, due to small differences in traveled path lengths, echoes from cross-track surface features will exhibit slightly different phases (Haynes et al. 2018a). Assuming that both the sub-surface nadir target and the surface off-nadir clutter will have similar phase measurement errors, the phase measurement accuracy should not be larger than half of the minimum interferometric phase of clutter minus any unknown interferometric phase bias. The actual phase measurement accuracy is taken as one standard deviation of the interferometric phase which depends on SNR and number of independent samples.

**Fig. 11:**
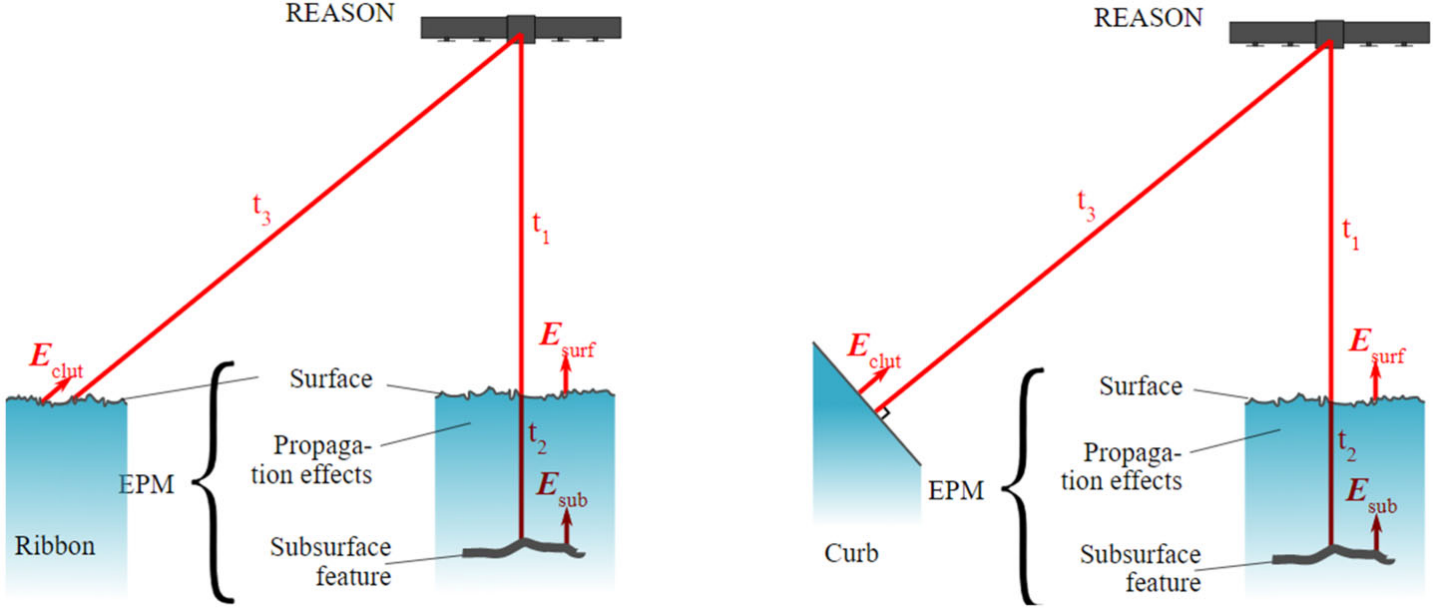
Illustration of the geometry involved in *interferometry*, including two representations of off-nadir surface features that could generate clutter, where “ribbons” represent rough, flat terrain and “curbs” represent smooth, angled terrain (EPM refers to Europa Point Model, Sect. 4.3)

By quantifying the interferometric phase between echoes recorded on the two cross-track VHF receive channels, we will discriminate whether the echoes are likely due to a subsurface nadir target or a cross-track off-nadir surface feature (Fig. 11). The magnitude of the interferometric phase between the echoes recorded by the two cross-track VHF receive channels will depend on the cross-track look-angle to the feature generating the reflection. A channel-to-channel phase measurement accuracy of 9° allows discrimination of clutter from the shallowest nadir sounding targets (150 m at 400 km altitude). For lower altitudes and deeper depths, the look angle (and the interferometric phase) of the corresponding surface clutter (at equivalent range) increases. However, points of ambiguity exist as the phase difference cycles over a wavelength. The capacity to use the two REASON VHF cross-track channels to separate echoes via phase will be a function of the SNR of the echoes, the phase stability of the VHF channels, and internal phase differences between the two VHF channels.

#### Plasma Characterization (HF, VHF)

The dispersion of the HF radar signal due to plasma and particles in the exosphere can be used to invert for the TEC below the spacecraft. Because increasing electron density introduces frequency-dependent phase shifts in radar signals (Safaeinili et al. 2003, 2007; Campbell et al. 2011, 2013b; Campbell and Watters 2016), the relative delay between the HF and VHF surface echoes can be used to infer TEC (Scanlan et al. 2019). We will use inversion techniques proven with MARSIS and SHARAD data (e.g., maximizing surface echo contrast) and verify our analyses through comparison to largely nondispersive VHF altimetry (Scanlan et al. 2019). However, this is a different approach than the autofocusing methods implemented for Martian radar sounders (Safaeinili et al. 2003, 2007; Campbell et al. 2011; Campbell and Watters 2016) as the 1 MHz REASON HF bandwidth is not wide enough to exhibit appreciable defocusing effects such as are observed in MARSIS and SHARAD data and no defocusing is expected across the REASON VHF band (Grima et al. 2015).

This inversion allows ionospheric correction for HF *sounding* (Mouginot et al. 2008) with simultaneous searching for active plumes, cryovolcanic eruptions of water originating from Europa’s subsurface, indicated by localized TEC anomalies caused by the ionization of plume-supplied neutral particles (Cartacci et al. 2013). Concurrent observations between the Plasma Instrument for Magnetic Sounding (PIMS) (see Westlake et al. 2023, this collection) and REASON instruments would provide electron densities at the spacecraft location along with the TEC between the spacecraft and surface. This combination could better constrain the local ionospheric density profile important for quantifying the plasma dynamic interaction at Europa and any potential contributions from the ionosphere to the observed induction signature.

#### Ranging (VHF)

Whereas *altimetry* refers to measuring height profiles along-track by identifying the surface return, *ranging* is intended to provide a differential range to a crossover point by combining stereo-derived Digital Terrain Models (DTMs) from the Europa Imaging System (see Turtle et al. 2024, this collection) with REASON radargrams. *Ranging*, when combined with orbit determination, allows the tidal Love number, h_2_, to be estimated directly (Mazarico et al. 2014). Simple confirmation of the ocean’s existence, via measurement of h_2_, requires sampling the amplitude of the tidal deformation with a spatial and temporal distribution that adequately samples the tidal potential (Sect. 2.3.4). REASON will make use of accurate orbit determination, in coordination with radio science, to contribute to determining the amplitude of gravitational tides using *ranging* at crossover points. Here, crossover points provide differential range measurements (Steinbrügge et al. 2018).

## REASON Science

### Driving Hypotheses, Science Objectives, and Strategic Science Guidance

The REASON instrument has been designed to test three key hypotheses:

1. The ice shell of Europa hosts lenses of liquid water and near-surface brines.
2. The ice shell overlies an ocean decoupled from the silicate interior and is subject to tidal flexing.
3. The exosphere, near-surface, ice shell, and ocean participate in exchange essential to the habitability of this moon.

To test these hypotheses and support future exploration, REASON will accomplish the following science objectives:

1. Characterize the distribution of any shallow subsurface water;
2. Investigate the processes governing material exchange among the ocean, ice shell, surface, and exosphere;
3. Search for an ice–ocean interface and characterize the ice shell’s global thermophysical structure;
4. Constrain Europa’s radial tidal deformations.

The Europa Clipper science team has defined four REASON Guiding Science datasets that are derived from the REASON driving hypotheses and objectives (Table 1) and directly contribute to Europa Clipper’s Level 1 (L1) science requirements (see Pappalardo et al. 2024, this collection). Seven additional REASON Extended Science datasets also compatible with these hypotheses and objectives will be collected in the course of REASON operations (Table 1). The requirements associated with these extended datasets are referred to as Planning Guidelines.

**Table 1:** Datasets for REASON Guiding and Extended Science

	REASON Dataset Name	Measurements	Description	Domain	Coverage
REASON Guiding Science	Near-Surface Properties	Reflectometry (HF, VHF) Altimetry (VHF)	Determine regolith cohesiveness, thickness, and subsurface layering; surface roughness and slopes; and the distribution of blocks	Surface to Near-Surface	Global Mapping
	Shallow Subsurface	Sounding (HF, VHF) Reflectometry (HF, VHF) Interferometry (VHF) Altimetry (VHF)	Map the distribution of subsurface water, near-surface brines, ice shell structure, and exchange processes by characterizing the electromagnetic properties and interface geometries	Shallow Subsurface	Global Mapping
	Full Depth Subsurface Exchange			Full Depth Subsurface	Global Mapping
	Ice-Ocean Interface	Sounding (HF, VHF) Altimetry (VHF)	Search for an ice-ocean interface by characterizing the surface elevation and ice shell thermophysical properties, as well as searching directly for any ice-ocean interfaces	Ice-Ocean Interface	Global Search
REASON Extended Science	Tidal Deformation	Ranging (VHF)	Characterize the topographic signature of the tidal shape by constraining the second-degree Love number h_2_ to 0.3 absolute accuracy, to confirm the existence of a subsurface ocean and constrain the ice shell thickness	Ice–Ocean Interface	Intersections distributed with respect to orbital position and geography
	Ice Shell Thermal Anomaly Search	Sounding (HF, VHF) Reflectometry (HF, VHF) Altimetry (VHF)	Characterize the thermal signatures of current or recent geological activity	Full Depth Subsurface	Global Mapping
	Subsurface Landform	Sounding (HF, VHF) Reflectometry (HF, VHF) Interferometry (VHF) Altimetry (VHF)	Characterize the subsurface structure, near-surface characteristics, and surface topography of major geologic landforms	Shallow Subsurface	Global Mapping
	Shallow Composition	Reflectometry (HF, VHF) Altimetry (VHF)	Assess the composition of surface materials, the geological context of the surface, the potential for geological activity, the existence of near-surface water and the potential for active upwelling of ocean-derived material	Surface to Near-Surface	Global Searching
	Surface Activity Evidence	Reflectometry (HF, VHF)	Search for spatial variations in surface density that are indicative of plume deposits	Near-Surface	
	Plasma Column Density	Plasma Characterization (HF, VHF)	Characterize the TEC of the local ionosphere with a detection threshold and measurement accuracy value of 3 × 10^14^ m^−2^.	Exosphere	
	Plume Search	Plasma Characterization (HF, VHF)	Search for active plumes below the spacecraft with a detection threshold TEC value of 3 × 10^14^ m^−2^	Exosphere	

### Requirements for Coverage Quality

Requirements imposed on the groundtrack distribution of flybys are similarly organized by the REASON Guiding Science datasets, and are specified in terms of panels, segments, and intersections. Panels are used to divide Europa’s surface into 14 subsections defined by latitude and longitude, which sample domains of Europa with properties that differ due to variations in tidal dissipation, surface temperature, and ocean forcing (see Pappalardo et al. 2024, this collection). Segments represent portions of the groundtrack within a panel where REASON measurements can be obtained (e.g., within altitude limits). Intersections are significant because they enable the independent registration and extension of 2D profiles, collected along segments, to understand 3D subsurface structure. Analysis of signal character at intersections additionally represents a path towards improved relative radiometric calibration and ensures consistent geologic interpretation over the mission.

Global coverage is considered achieved when ≥11 of the 14 panels are sampled by groundtrack segments. For all REASON Guiding Science datasets except for the Ice–Ocean Interface dataset, groundtrack segments must be at least 800 km in length, below an altitude of 400 km, and intersect multiple times (2 anti-Jovian and 1 sub-Jovian) to count toward global coverage. Note that global coverage is skewed towards the anti-Jovian hemisphere where both frequencies can operate (i.e., where the HF is shielded from Jovian decametric noise). For the Ice–Ocean Interface dataset, groundtrack segments are not required to intersect, but must be at least 1600 km in length, below an altitude of 1000 km. To search for an ice–ocean interface, correlated gradients in ice thickness and surface elevation must be tracked at the panel scale (∼1600 km per side).

This coverage supports a detailed characterization of the following ice shell domains (Fig. 9 and Table 1): (i) the bounding exosphere — composed of ionic species which are either sputtered from Europa’s surface or transported from the subsurface via plumes (ii) the surface to near-surface — likely heavily modified by Jovian system radiation products and any plume activity, (iii) the shallow subsurface (i.e., the upper few kilometers) — likely dominated by brittle fracture, (iv) the full depth subsurface (i.e., to tens of kilometers) — which has the potential for ductile rheologies that support convection of the deep interior and interfaces defined by phase transitions (e.g., eutectics^7^), as well as (v) the ice-ocean interface — modulated by accretion (freezing) and ablation (melting) processes and whose existence has implications for tidal deformation of the entire ice shell.

### Probing Europa from Exosphere to Ocean

Europa’s habitability is in part governed by the availability of oxidants and reductants, which life can harness as energy through redox reactions (McCollom 1999; Zolotov and Shock 2004; Hand et al. 2009). Expected redox disequilibria between reduced suboceanic rocks and corresponding H_2_-, H_2_S-bearing fluids, and oxidized oceanic water species (sulfate, bicarbonate) should provide metabolic energy for microorganisms (McCollom 1999; Zolotov and Shock 2004). In addition, Europa’s ice shell is thought to supply strong oxidants (e.g., O_2_, H_2_O_2_), which are radiolytically produced on the surface (Chyba 2000). Constraining exchange between surface materials and the ocean is essential to constrain Europa’s habitability–the main objective of the Europa Clipper mission. By probing Europa from exosphere to ocean, REASON will provide insights into processes of surface–ice–ocean exchange and the properties of the ice shell which modulate them.

#### Exosphere

Much of our knowledge of the magnetospheric environment of Europa comes from Galileo observations (Gurnett et al. 1998; Kurth et al. 2001; Bagenal et al. 2016, 2017). These are largely due to inference from the upper hybrid resonance frequency which, with the measured magnetic field, allows a determination of the electron plasma frequency, hence electron density. Kurth et al. (2001) showed that the electron density near Europa’s orbit could vary from about 50 cm^−3^ to over 500 cm^−3^ although the latter was an exceptional case observed near the E12 flyby. Typical values are closer to 100 cm^−3^. Ten vertical electron density profiles through Galileo radio occultations during the E4, E6, E19 and E24 flybys (Kliore et al. 1997; McGrath et al. 2009) reveal a mostly surface-bound ionosphere (i.e., with the highest density layer at or near the surface) except for one profile exhibiting a maximum electron density of $\sim 10^{4}\text{ cm}^{-3}$ at ∼100 km altitude. The plasma scale height is reported to be 240 ± 40 km near the surface and 440 ± 60 km above 300 km, and the TEC can reach up to $\sim 4\times 10^{15}\text{ m}^{-2}$. However, three occultation detections were negative (i.e., weak to non-detection) near the downstream wake region of Europa, supposedly due to the absence of ionization processes there at the time of observation.

The spatial and temporal variability of Europa’s exosphere results from concurrent ionospheric production processes prevailing in specific hemispheres independently rotating with different time constants: solar photoionization in the day side and impact ionization predominant in the trailing side from the Io plasma torus. The average ionosphere production rate from impact ionization exceeds photoionization (Saur et al. 1998). However, their combined action in asymmetrically wrapping Europa with an ionosphere, setting its exobase and pacing its overall temporal variability is still poorly explained (Bagenal and Dols 2020). Plumes represent another potential source of spatial and temporal variability. A re-examination of data collected during the E12 Galileo flyby revealed fluctuations in the magnetic field, including a possible peak in the electron density exceeding 2000 cm^−3^ (Jia et al. 2018). From these data, Jia et al. (2018) concluded that it was possible Galileo had passed through a plume with characteristics consistent with the putative plumes detected by Hubble (Roth et al. 2014).

REASON *plasma characterization* will enhance our understanding of electron density in the exosphere by characterizing the TEC in the nadir column between the spacecraft and Europa’s surface, complementing data obtained with PIMS. Additionally, REASON *plasma characterization* will support the search for active plumes alongside other remote sensing (e.g., Europa Ultraviolet Spectrograph (Europa-UVS), EIS, the Mapping Imaging Spectrometer for Europa (MISE), and the Europa Thermal Emission Imaging System (E-THEMIS)), and in situ (e.g., Europa Clipper Magnetometer (ECM), PIMS, the SUrface Dust Analyzer (SUDA), and the MAss Spectrometer for Planetary Exploration (MASPEX)) instruments. REASON’s ability to search for localized ionospheric anomalies possibly related to active plumes is limited to portions of the groundtracks where both HF and VHF data can be acquired (i.e., the anti-Jovian hemisphere). Finally, as REASON can only infer TEC in the nadir column between the spacecraft and Europa, the flyby tour geometry implies REASON will be least sensitive to active plumes altering the Europan ionosphere near closest approach, where the intervening ionosphere is the thinnest.

#### Surface to Near-Surface

Although the surface of Europa is largely dominated by water ice, a range of processes can alter its composition and properties. Exogenic processes like impact gardening through charged particles and micrometeorite bombardment can alter the chemical composition and porosity of the endogenous upper surface layers to a depth of 30 cm on average (Moore et al. 2009; Costello et al. 2021). Quantification of this process and its spatial distribution is necessary for assessing the delivery of radiolytically-produced oxidants into the deeper ice shell and down to the ocean (Hand et al. 2007). Radiation processes from charged particles can drive thermal sintering, increasing the strength and density of the surface at time scales <100 Myr (Molaro et al. 2019; Choukroun et al. 2020). REASON is highly suited to studying these processes due to the sensitivity of *reflectometry* to properties of the near-surface. For example, on Earth the reflection coefficient from fresh snow to compact ice spans a radiometric response of almost 30 dB (Grima et al. 2014a, 2016).

Spectroscopic evidence of salts associated with resurfacing features suggests endogenic material, possibly sourced from the sub-ice ocean, is being transported to the surface (Brown and Hand 2013; Fischer et al. 2015; Trumbo et al. 2019, 2020, 2022), consistent with hypotheses for the infiltration of subsurface brines into near-surface porous regolith (Head and Pappalardo 1999; Schmidt et al. 2011). Ephemeral liquid brines near the surface could be detected by *reflectometry* as a region that is anomalously bright relative to the surrounding near-surface (Grima et al. 2016). However, liquid brines near the surface are thermodynamically unstable and would rapidly freeze (Abramov and Spencer 2008), forming layer(s) of saline ice and hydrated salts within the porous regolith (Zolotov and Shock 2001; Thomas et al. 2017).

These layers, analogous to frozen melt lenses in firn, likely have a distinct scattering signature that can be similarly distinguished by *reflectometry* (Rutishauser et al. 2016; Chan et al. 2023b). Salt lag deposits, caused by sublimation of ice associated with frozen brines at the surface, could form additional layers (Zolotov and Shock 2001); however, the *reflectometry* signature associated with such features has not yet been evaluated. Fossil and/or contemporary plume fallout deposits represent another source of layering at Europa’s surface (e.g., Fagents et al. 2000; Fagents 2003). Near-surface densities derived from REASON reflectometry measurements (Fig. 3) will aid in distinguishing high porosity (0.5 to 0.9; Quick and Hedman 2020) plume fallout deposits from a conventional Europa surface. Because REASON’s VHF and HF frequencies respond differently to different layer thicknesses, properties, and depths (Mouginot et al. 2009; Scanlan et al. 2022; Chan et al. 2023b), comparing VHF and HF *reflectometry* results could provide additional insights. For candidate plume fallout deposits identified by EIS, this could constrain plume material flux and volume, longevity, and morphology (Scanlan et al. 2022), whereas for frozen brine layers, this could constrain layer thickness and depth (Chan et al. 2023b).

Surface temperature variations that may or may not be related to the presence of liquid brines near the surface will primarily be assessed through measurements made by the E-THEMIS (see Christensen et al. 2024, this collection). REASON *reflectometry* will supplement E-THEMIS investigations in the search for thermal anomalies by constraining porosity variations (Grima et al. 2014a) or roughness smoothing (Grima et al. 2014b) that could result from the cooling of a warm body. The skin depth of REASON *reflectometry* is also greater by one to two orders of magnitude than E-THEMIS, allowing surface properties to be reconciled with properties at depth within the near-surface.

#### Shallow and Full Depth Subsurface

Voyager and Galileo spacecraft observations of Europa’s surface revealed a rich variety of landforms (see Daubar et al. 2024, this collection) that suggest active geological processes and surface-interior exchange occurred at some period within the geological history of the shell (<100 Myr). Studies focused on the morphology of these features have prompted a range of hypotheses for processes occurring within the outer ice shell, many of which have involved the presence of shallow subsurface water.

The presence of liquid water in the shallow subsurface is both a driver and byproduct of hypothesized mechanisms of surface-ice-ocean exchange, all of which are likely modulated by tidal forcing. Ocean water may be directly injected into the ice shell interior through basal fractures (Michaut and Manga 2014; Craft et al. 2016), whereas others have proposed that water may be generated through ice shell melting caused by convection (Kalousová et al. 2017; Vilella et al. 2020), diapirism (Pappalardo et al. 1998; Nimmo and Manga 2002; Sotin et al. 2002; Pappalardo and Barr 2004; Schmidt et al. 2011), and/or strike-slip displacement (Kalousová et al. 2016; Gaidos and Nimmo 2000; Hammond 2020; Nimmo and Gaidos 2002).

The morphologies of some double ridges, as well as lenticulae,^8^ suggest subsurface sills of water may have been present at the time of formation (Dombard et al. 2013; Johnston and Montési 2014; Michaut and Manga 2014; Manga and Michaut 2017; Singer et al. 2021; Culberg et al. 2022). Similarly, the unique morphology of chaos terrains is hypothesized to be associated with warm ice, partial melt, and/or liquid water (Collins et al. 2000; Collins and Nimmo 2009). Based upon studies of surface topography, Schmidt et al. (2011) suggest that chaos terrain is formed by the collapse and refreezing of active subsurface water reservoirs. In the case of Thera Macula, depressions in topography suggest some part of the water lens could still be active (Schmidt et al. 2011). Recent imagery of Europa collected by the Juno Stellar Reference Unit revealed a new chaos feature with morphology consistent with the lens collapse hypothesis proposed for Thera Macula, as well as low albedo materials that could be evidence of brine infiltration (Becker et al. 2023).

Further features of interest are impact structures. While craters are rare on Europa’s surface, larger impact structures, such as Manannán crater, likely resulted in the formation of substantial melt reservoirs (Steinbrügge et al. 2020a; Carnahan et al. 2022). Callanish and Tyre are the only known multi-ring impact features on Europa (see Daubar et al. 2024, this collection). Their size and morphology make them the best candidates for complete penetration of the ice shell by impacts (Moore et al. 2001; Cox and Bauer 2015; Singer et al. 2023). As such, studying their subsurface morphology is likely to provide insights into the properties of the ice shell at the time of impact.

Liquid water within the shallow subsurface could remain liquid for up to a few hundred thousand years, depending on the reservoir volume and depth (Abramov et al. 2013; Manga and Michaut 2017; Chivers et al. 2021). However, as freezing progresses, these water bodies pressurize and cryoconcentrate. Overpressurization can induce cryovolcanism, forming plumes (Steinbrügge et al. 2020a; Lesage et al. 2022), whereas cryoconcentration can cause salts to precipitate, forming salt layers (Buffo et al. 2020; Chivers et al. 2021). Low albedo regions associated with ridges, ridge complexes, and lenticulae have been interpreted as potential indicators of cryovolcanism that accompanied formation/evolution of these geological features (Greenberg et al. 1999; Fagents et al. 2000; Fagents 2003; Mitri and Showman 2005; Prockter and Schenk 2005).

Because Europa’s surface features are likely direct expressions of the subsurface, REASON’s *sounding* and *altimetry* measurements will be able to differentiate between various hypotheses for the formation of these features by quantitatively characterizing the surface while simultaneously offering a window into the subsurface. REASON is capable of directly detecting the presence and distribution of subsurface water in the shallow subsurface on Europa by *sounding* the ice shell for sharp reflections from perched water bodies as well as brine-rich and/or salt-rich features. Reflections from vertical cracks infilled with liquid water have a distinct “corner-reflector” signature in unfocused radar data (Peters et al. 2007b) and may be detectable by REASON. If a plume were to be detected, REASON *sounding* will aid in isolating the source of the eruptive material, complementing observations from other investigations (e.g., E-THEMIS, see Christensen et al. 2024, this collection; EIS, see Turtle et al. 2024, this collection).

REASON *altimetry* will measure the elevation of surface features with horizontal extents spanning tens of kilometers. For features with detectable subsurface interfaces, such as ice blocks and chaos matrix regions, complementary *sounding* and *altimetry* measurements will enable interfaces at depth to be tested for hydrostatic compensation to confirm the presence of liquid water. Even in the absence of active liquid water, subsurface interfaces detected by *sounding* can be evaluated in the context of the surface topography provided by *altimetry* to evaluate the potential for relict liquid water (e.g., Culberg et al. 2022). Digital elevation models (DEMs) provided by EIS will provide additional context for interpretation of subsurface interfaces detected by *sounding*. REASON *interferometry* and cluttergrams^9^ generated from EIS DEMs will ensure clutter from off-nadir surface features is not mistaken for subsurface interfaces at nadir.

#### Ice-Ocean Interface

Measuring the tidal Love numbers h_2_, k_2_, and l_2_ and linear combinations of them has been suggested as a viable method to constrain the thermal and interior structure of icy satellites (Moore and Schubert 2000; Wahr et al. 2006). Whereas changes in the tidal potential can be determined by measuring the time-varying gravity field with radio Doppler tracking (see Mazarico et al. 2023, this collection), the determination of the radial deformation, parameterized by h_2_ requires altimetry measurements over several tidal cycles at different locations. Crossover points at which two altimetric measurements at the surface are taken at a different tidal phase are a means for determining tidal amplitudes. By combining altimetric data, in particular crossovers from REASON with surface topography from stereo-photogrammetric techniques with EIS, the determination of h_2_ is possible provided the signal is large enough (Steinbrügge et al. 2018). Numerical simulations of Europa’s tidal response (1.8-day semidiurnal tides) predict that tidal deflections are an order of magnitude larger if an ocean is present (∼30 m vs. 1 m) (Moore and Schubert 2000). Thus, h_2_ determination would provide evidence for (or against) a Europan subsurface ocean, complementary to subsurface *sounding* or other evidence from gravity field determination or magnetic field induction.

Typical values for h_2_ are around 1.2 in the presence of an ocean and drop below 0.1 if no ocean is present (Moore and Schubert 2000). Using the estimated error bounds for h_2_ alone would allow unambiguous confirmation or rejection of the hypothesis of a global ocean (Steinbrügge et al. 2018). To constrain the thickness of the ice shell a combination of both Love numbers $1+\mathrm{k}_{2}-\mathrm{h}_{2}$ would be most promising (Wahr et al. 2006). In that case the ice thickness could be constrained within an uncertainty of 15 km, provided rheological parameters of the ice shell (rigidity and viscosity) are known (Steinbrügge et al. 2018). The best constraints on the viscosity of the ice shell could be obtained if the phase-lag, the deviation of the tidal bulge from the sub-Jovian line, could be measured. However, such a measurement would be even more challenging and could only be obtained with REASON *ranging* if the ice shell is very dissipative. That would imply a phase-lag of a few degrees, which might be detectable if the deformation is strong enough. In case both phase lags in k_2_ and h_2_ could be measured, the phase-lag difference could further constrain whether strong tidal dissipation is occurring only in the ice shell or if the silicate mantle and/or crust of Europa is dissipative as well (Hussmann et al. 2016). Optical determination of Europa’s physical libration (Love number l_2_), as was done for Enceladus (Thomas et al. 2016), would further constrain Europa’s tidal dissipation properties.

Studies of the thickness of Europa’s ice shell and its thermal state – thick or thin, convective or conductive – have spawned competing hypotheses. Estimated ice thicknesses have ranged between ∼3 km (Carr et al. 1998; Greenberg et al. 1999; Walker and Rhoden 2022) and ≳30 km (Cassen et al. 1979; Squyres et al. 1983; Pappalardo et al. 1998; Schenk 2002; Vilella et al. 2020). Many of the estimates for a thin ice shell are based upon mechanical models (e.g., Billings and Kattenhorn 2005) and likely reflect an ice shell thickness earlier in Europa’s history. For Europa, the ice shell thickness and heat flow are inextricably linked. Heat flow is a fundamental property that determines a body’s level of internal activity and thermophysical structure. Actively deforming regions on Europa are hypothesized to exhibit brittle-ductile and ductile-plastic transitions at depth (Goldsby and Kohlstedt 2001; McCarthy et al. 2011). Thicker (≳10 km) ice shells are predicted to undergo solid-state convection at depth (McKinnon 1999; Barr and Showman 2009). A recent thermodynamic study suggests that the total ice shell thickness is likely >20 km, where the conductive layer thickness is ∼10 km (Howell 2021).

REASON *sounding* will help to resolve the ice shell thickness debate by searching globally for an ice–ocean interface indicated by a sharp, continuous (>1000 km long) radar reflection due to any major ice–water permittivity contrast up to a depth of 30 km below the surface. This involves tracking surface topography and the depth of the lower interface over gradients in ice shell thickness. To first order, these gradients in ice shell thickness are expected to be at the hemispheric scale (i.e., pole to equator). Because the ice–ocean interface is at the melting temperature (<273 K), and radar attenuation is strongly temperature-dependent (Fig. 10), the ice-ocean interface will likely be challenging to detect. If the ice shell is purely conductive, detection should be possible since these losses will be confined to the warm region close to the ice-interface. In a convecting ice shell, this warm region potentially spans the entire convective layer, resulting in large radar signal attenuation. As such, direct detection of the ice–ocean interface by REASON at the hemispheric scale will be unlikely if the ice shell is convective; although, the presence of a eutectic interface at the upper parts of convective cells might be detectable (Zolotov and Kargel 2009; Kalousová et al. 2017; Culha et al. 2020). Additionally, it has been hypothesized that cold downwellings produced by convection may provide a distribution of low attenuation windows into the ocean–ice interface (McKinnon 2005; Kalousová et al. 2017). In summary, by detecting spatially variable zones of anomalous radar scattering and absorption, REASON *sounding* will reveal the thermophysical structure of the ice shell, placing constraints on the heat flow. These data will also inform occurrences and sizes of convective cells, as well as regions of convective ice upwelling and downwelling (Kalousová et al. 2017). Note that detection of any interface serves as a constraint on minimum ice shell thickness.

## Requirements for Measurement Quality

There are nine types of requirements imposed on measurement quality that apply to the four baseline measurement techniques. These types of requirements are summarized in Table 2 and defined in Sects. 3.1–3.7.

**Table 2:** Requirements on measurement quality organized by baseline measurement technique

		Baseline Measurement Techniques			
		Sounding	Altimetry	Reflectometry	Interferometry
Requirements on Measurement Quality	Blind Zone	**HF/VHF**: <7× vertical resolution from the surface in ice	N/A	N/A	N/A
	Vertical Resolution	**VHF**: ≤30 m in ice (finer) in top 3 km **HF**: ≤300 m in ice to 30 km depth **HF/VHF**: ≤300 m in ice (coarser) from 3 km to 30 km depth	N/A	N/A	N/A
	Vertical Precision	**VHF**: ≤15 m in ice (finer) for top 3 km **HF**: ≤150 m in ice to 30 km depth **HF/VHF**: ≤150 m in ice (coarser) from 3 km to 30 km depth	**VHF**: ≤15 m in vacuum	N/A	N/A
	Along-Track Resolution	**VHF**: ≤2 km (finer) **HF**: ≤5.5 km **HF/VHF**: ≤10 km (coarser)	**VHF**: ≤2 km (finer) **HF/VHF**: ≤10 km (coarser)	N/A	N/A
	Sampled Along-Track Resolution	N/A	N/A	**VHF**: ≤10 km **HF**: ≤27.5 km	N/A
	Clutter Discrimination	N/A	N/A	N/A	**VHF**: >10 km for 80% of any groundtrack to 3 km depth
	Radiometric Precision	N/A	N/A	**VHF**: 1 dB over ≤ 10 km **HF**: 1 dB over ≤ 27.5 km	N/A
	Radiometric Accuracy	N/A	N/A	**VHF**: 1 dB over ≤ 10 km **HF**: 1 dB over ≤ 27.5 km	N/A
	Radiometric Stability	**HF**: 2 dB within a flyby in the subsurface	N/A	N/A	N/A

*We compare the different resolution requirements provided by the 10 MHz VHF bandwidth and the 1 MHz HF bandwidth using the terms “finer” for a smaller numeric requirement and “coarser” for a large numeric requirement.

### Blind Zone

It is expected that strong surface returns from a smooth and flat ice surface will lead to side lobes^10^ in the range echo whose power levels are proportional to that of the main lobe, and which can rise above the background noise level. The region where these side lobes constitute the noise floor is called the blind zone, although some bright reflectors may still be visible. The range extent and power level of each side lobe needs to be controlled or suppressed so that reflections from shallow subsurface interfaces could be detected. By applying a processing window to the receive echo, the range extent and power level of the side lobes can be reduced. The blind zone is required to be less than seven times the vertical resolution in ice, which corresponds to the predicted range extent of the side lobes assuming a Hann window.

### Vertical Resolution and Precision

Vertical resolution governs the theoretical minimum spacing between two reflecting interfaces that can be distinguished by the radar. A VHF vertical resolution of 30 m in ice, over an along-track resolution cell, is required to confidently separate subsurface features, as well as for clutter discrimination from subsurface features. 10% of the thickness of a 300 m thick floating ice block in water is ∼30 m, so a vertical resolution of at least 30 m is required to confidently map the subsurface. In Europa’s chaos regions, the lower range of ice block thicknesses is expected to be nearly 300 m thick (Nimmo and Giese 2005). This requirement is for two reflectors with the same returned power. A HF vertical resolution of 300 m, over an along-track resolution cell, is required to separate surface from subsurface return of objects such as liquid water lenses and bands (Blankenship et al. 2009). For example, resolving the ∼300 m thickness of a floating ice block requires a *sounding* vertical resolution of 300 m in ice. 3 km is the best estimate of the depth of eutectic lenses; 300 m is 10% of this.

In practice, it is the vertical precision, and not the vertical resolution, which limits the precision of a range estimate to an isolated target. The vertical precision is a function of both the vertical resolution and SNR, where vertical precision is improved with increasing SNR (Cavitte et al. 2016). Requiring the vertical precision be at least a factor of two better than the vertical resolution limits the uncertainty associated with depth and orientation of reflectors critical to testing for flotation or embayment. As such, a VHF vertical precision of 15 m in vacuum, over an along-track resolution cell, is required to measure the range to surface features such as pits, domes, and diapirs, whereas a VHF vertical precision of 15 m in ice, over an along-track resolution cell, is required to constrain the depth and orientation of subsurface structures.

### Along-Track Resolution

Along-track resolution governs the ability to discriminate adjacent features in *altimetry* and *sounding* data along an interface in the direction of the satellite groundtrack. A finer HF/VHF along-track resolution of 5.5 km/2 km allows us to characterize a wide range of resurfacing features (including pits, domes, and chaos) and resolve ice–water interfaces, hydraulic potential beneath floating blocks up to altitudes of 400 km. A coarser along-track resolution of 10 km allows any ice–water interface as well as associated thermophysical structures to be resolved up to altitudes of 1000 km.

### Sampled Along-Track Resolution

The sampled along-track resolution ensures statistical robustness of observations in support of *reflectometry*. Ideally, two consecutive pulses should be independent for best statistics recovery. However, the along-track spacing to ensure independence from pulse to pulse is not formally documented in the literature, but this length should vary with roughness. It is infinite in the end case of reflections from a perfectly flat surface, but only one surface echo is then necessary to recover the delta function that characterizes its statistical distribution. Then, the along-track spacing is expected to decrease with increasing roughness as the randomness of the scatterers increases. We associate the necessary along-track spacing to the radius of the effective area of constructive interference, i.e., the area of a self-affine surface beyond which constructive addition has dropped significantly (Eq. 20 in Shepard and Campbell 1999). This approximates to about 20% of the wavelength for the rougher terrains measured at Europa (Steinbrügge et al. 2020b). This minimum spacing is sufficient to recover robust statistics from 1000 empirical echoes spaced by 1 m at a 5-m wavelength for airborne observations (Grima et al. 2016). The VHF along-track resolution ≤10 km/27.5 km at spacecraft altitude ≤400 km is required to constrain surface statistical echo properties to discern porous or impermeable ice and liquid or frozen brines or brine-soaked ice. The 10 km/27.5 km comes from the requirement that a patch must be at least five times the along-track resolution of *sounding* measurements (2 km/5.5 km) for sufficient sampling. This requirement assumes that >1000 samples will be collected over the 10 km/27.5 km region for statistical robustness. Although HF has a reduced along-track resolution compared to VHF, it has a higher capacity and robustness to penetrate deeper regions where liquid water may be present. This HF requirement is optimized for *sounding*. HF and VHF operate as complementary wavelengths.

### Clutter Discrimination

REASON’s VHF beam pattern (Fig. 25) is such that echoes from cross-track, off-nadir surface features can arrive at the spacecraft and be recorded at the same time as nadir subsurface echoes from the upper 3 km of the ice shell. This introduces an inherent ambiguity as to whether received echoes originate from a subsurface target of interest or a surface feature. As such, in order to properly interpret the resulting radargrams, it is important that these “clutter” features be discriminated from subsurface nadir targets. For the VHF shallow *sounding* and *interferometry* measurements in the Shallow Subsurface and Full Depth Subsurface Exchange datasets, the REASON VHF will discriminate between surface clutter and subsurface nadir features longer than 10 km at observation depths between the near-surface blind zone to 3 km for greater than 80% of any groundtrack at altitudes between 35 km and 400 km. A groundtrack percentage of 80% is derived from a 95% probability of successful clutter discrimination at an intersection. If we can discriminate subsurface features from surface clutter at an intersection, we know that the extension of the reflector across both groundtracks (assuming the reflector is continuous) is a subsurface feature and not clutter (Castelletti et al. 2017; Haynes et al. 2018a; Scanlan et al. 2020).

### Radiometric Precision and Accuracy

The radiometric precision requirements ensure the ability to measure relative power, without azimuth gain, over the required reflectometry resolution cell for HF and VHF *reflectometry*. This requirement allows the statistics to be constrained over the resolution cell, and the incoherent portion of the energy be measured. The radiometric accuracy requirements ensure the ability to measure relative power, without azimuth gain, over a REASON flyby for HF and VHF *reflectometry*. This requirement allows the coherent energy to be compared across the profile, and thus observe variations in the permittivity of the near-surface. A precision and accuracy of 1 dB on the HF/VHF signal over length scales of less than or equal to 27.5 km/10 km within a given groundtrack allows us to resolve the coherent and incoherent energy variations corresponding to the smallest difference in permittivity across the full range of snow densification phases observed in Earth analogs, specifically the difference between compact hexagonal ice (reflectivity: −11 dB, permittivity: ∼3.15) and ice at pore close-off (reflectivity: −12 dB, permittivity: ∼2.8) (Grima et al. 2014a). We note that there is uncertainty in the empirical laws used to relate density and permittivity, which could be addressed by future laboratory measurements (Sect. 9.1.1).

Because the 1-dB precision and accuracy requirement is imposed on the total power measured by the receiver, this does not represent the precision and accuracy of the coherent and incoherent power derived from reflectometry, which is ultimately governed by the relative balance of these two quantities. If the coherent and incoherent power are within 5 dB of each other (coherent/incoherent power ratio between −5 dB and 5 dB), the uncertainty is relatively stable and corresponds to a bias on the predicted coherent and incoherent power of less than 1 dB (Grima et al. 2022b). The spacecraft altitude will also influence the coherent/incoherent balance since geometric spreading affects each term differently (Haynes et al. 2018b). Additional uncertainty will come from the strategy for absolute radiometric calibration (e.g., recording returns from well-characterized terrains (Grima et al. 2014a) or listening to well-characterized radio noise (Gerekos et al. 2024)).

### Radiometric Stability

The radiometric stability requirements ensure the ability to measure relative power for *sounding* to track variations in subsurface attenuation, accounting for drifts that could occur over a flyby and over shortened timescales. Attenuation as a function of depth within the ice shell can tell us about the structure of heat transfer and compositional variations within the ice shell (Kalousová et al. 2017). If the ice shell is convecting, radar attenuation rates may vary along a groundtrack. For a subsurface interface that changes 1 km in depth over tens of kilometers, a change in attenuation rate of 2 dB/km due to thermal/compositional variations requires 2 dB radiometric stability.

## Verification & Validation (V&V) for Requirements on Measurement Quality

### Overview of Science V&V Approach

Europa’s ice shell is a challenging target from the perspective of developing, verifying, and validating requirements for an ice-penetrating radar. REASON’s baseline measurement techniques and requirements on measurement quality are sensitive to properties of Europa, many of which are poorly constrained and continue to be debated. The permittivity ($\varepsilon = \varepsilon ' -j \varepsilon ^{\prime \prime }$) governs the radar signal attenuation, contrasts in permittivity produce reflections, and rough interfaces and the presence of voids can induce scattering losses. These phenomena govern the ability for a particular measurement to be successful for a given instrument performance.

Given the lack of constraints on the properties of Europa, an extremely wide parameter space of possible Europas currently exists. To ensure that REASON requirements on measurement quality are robust for a selection of plausible Europas, we evaluate requirements in the context of models for Europa consistent with the hypotheses REASON is designed to test. We represent Europa as 1D models to evaluate all REASON requirements on measurement quality, except for the requirement for interferometric clutter discrimination, because it is a 2D phenomenon.

Each 1D model is constructed using twelve geophysical regimes that describe Europa from exosphere to ocean (Sect. 4.2), sampling a range of published scientific hypotheses and incorporating Europa’s known properties. Because predictions of Europa’s geophysical character tend to be bimodal (e.g., thin versus thick ice shell), we define representative parameter values for two modes in each regime. Each geophysical regime in a point model contributes to terms in a 1D link budget that allows for the calculation of the SNR for a given target interface (Table 3). We include both subsurface water and subsurface structure as target interfaces and consider an SNR ≥ 0 necessary for a Measurement Quality Requirement to be met for a possible Europa (i.e., a target interface cannot be studied if it is not detectable).

**Table 3:** Terms included in REASON 1D link budget

	Link Budget Term	Description	Relevant Regimes	Reference
Performance, Operations, Geometry	Radar Potential	SNR of a flat, perfect, infinite reflector at a range of 400 km, assuming SAR processing	–	Sect. 4.4.1
	Reference SAR Gain	Gain resulting from SAR processing at the reference altitude (400 km)	–	Sect. 4.4.4
		Reference SAR Gain is subtracted from Radar Potential so that SAR Gain can be recalculated for the altitude of interest		
	Geometric Spreading	Loss due to spreading of the wavefront, calculated with respect to the reference altitude (400 km)	–	Schroeder et al. (2014) Haynes et al. (2018b)
	Flat Europa Assumption	Loss to account for a spherical (as opposed to flat) Fresnel zone area	–	Haynes et al. (2018b)
	Short Chirp	Loss associated with a chirp length (RF pulse width) below the 200 μs assumed in deriving the radar potential	–	Table 9
	SAR Gain	Gain resulting from SAR processing (not applied for Reflectometry)	–	Peters et al. (2005b)
	1-Bit Sampling	Loss (VHF only) resulting from recording only the signal sign below 3 km to conserve data volume	–	Scanlan et al. (2020)
	Infinite Mirror Assumption	Loss to account for spherical (as opposed to flat) wavefront	–	Haynes et al. (2018b)
	Range Side Lobe Noise	Loss due to side lobes (Hann processing window)	–	Sect. 4.4.5
Europa	Surface Scattering	Loss to due signal scattering from a rough surface	Surface Roughness	Fung et al. (1992)
	Surface Transmission	Loss to due signal transmission through a rough surface	Surface Roughness	Fung et al. (1992)
	Volume Scattering	Loss due to volume scattering from voids in the ice shell	Deep Regolith	Aglyamov et al. (2017)
	Ice Shell Attenuation	Loss due to electrical conductivity of the ice shell	Surface Temperature	Moore (2000)
			Ice Shell Composition	
			Ice Shell Thickness	
			Basal Temperature	
	Interface Reflection Coefficient	Fresnel reflection coefficient at the target interface	Near-Surface Density	Mouginot et al. (2009)
			Near-Surface Composition	
			Near-Surface Thickness	
			Salt Layer Thickness	
			Salt Layer Depth	
			Subsurface Water Interface	

Because there is uncertainty in the physical properties of Europa which govern major loss terms in the 1D link budget (e.g., the surface roughness at radar wavelengths, volume scattering, and ice shell attenuation), multiple point models are constructed and organized into Ensembles of Point Models (EPMs) (Sect. 4.3). These EPMs are connected to, but defined independently from, REASON’s Guiding Science Datasets. EPMs are used to V&V requirements on measurement quality and to validate requirements at the instrument level that are used to generate Key Radar Parameters (KRPs). The KRPs serve as a bridge between REASON Science and System Engineering, translating estimated instrument performance, including spacecraft-level impingements, to input parameters for verifying requirements on measurement quality (Sect. 3).

This approach to V&V is adopted to ensure the point models for Europa used to evaluate requirements are consistent with the hypotheses and measurement techniques associated with each dataset, since each dataset was defined according to specific hypothesis tests. Because the hypotheses are related to the structure and properties of Europa, they imply the selection of a particular mode for each regime as well as their combinations with other modes to form an EPM. A single requirement on measurement quality may be evaluated using multiple EPMs if the requirement traces up to multiple datasets. As a result, a given requirement on measurement quality may pass for one dataset and fail for another. This feature of the REASON science verification and validation approach allows for enhanced traceability to evaluate the impact of instrument performance for specific datasets.

### REASON-Relevant Europa Regimes

The twelve geophysical regimes and two modes used to represent REASON-sensitive properties of Europa are summarized in Table 4 and described in Sects. 4.2.1–4.2.10. We divide the geophysical regimes into two classes: one which applies to the surface and near-surface and another which applies to the subsurface. The near-surface regime modes only vary in the Altimetry and Reflectometry EPM, since the variations in the properties of the near-surface are not relevant to *sounding* the subsurface. Two regimes that are not included here are ionospheric dispersion and Jovian noise. Ionospheric dispersion causes a proportional delay in the echo receive time (much greater for HF than VHF) (Grima et al. 2015), and decametric noise from Jupiter can obscure HF echoes (Cecconi et al. 2012). However, these effects are mitigated by REASON’s requirements. HF ionospheric delays can be corrected using the relatively unperturbed VHF, and HF *sounding* measurements are not required on Europa’s sub-Jovian hemisphere.

**Table 4:** Geophysical regimes used to build 1D point models of Europa

	Regime	Mode 1	Mode 2
Surface & Near-Surface	Surface Roughness	Ridged Plains	Chaotic Terrain
		$\sigma _{HF}= 2.2\text{ m}$	$\sigma _{HF} = 6\text{ m}$
		$\sigma _{VHF} = 0.5\text{ m}$	$\sigma _{VHF}= 2.2\text{ m}$
	Density	Non-Porous	Porous
		910 kg m ^−3^	518 kg m^−3^
	Composition	Clean Ice	Brine-Filled Ice
		*ε* = 3.15	*ε* = 9
	Thickness	Thin	Thick
		0.5 m	200 m
Subsurface	Deep Regolith	Absent	Present
		Non-Porous	22% Porosity, 22 cm Pore Radius
	Surface Temperature	Polar	Equatorial
		50 K	100 K
	Ice Shell Composition	Pure	Marine
		0 *μ*M Cl^−^	60 *μ*M Cl^−^
	Ice Shell Thickness	Thin	Thick
		3 km	30 km
	Salt Layer Thickness	0.2 m	1 m
	Salt Layer Depth	1/2 Ice Shell Thickness	10 km
	Subsurface Water Interface	Sharp	Mush
		Water	30% Ice-Water Mixture
	Basal Temperature	Melting Temperature	Eutectic Temperature
		270 K	250 K

#### Surface Roughness

Surface roughness affects both the coherence and strength of the radar echo reflected from Europa’s surface (i.e., *altimetry* and *ranging*), as well as the coherence and strength of the transmitted signal component that continues to propagate downwards into Europa’s subsurface (i.e., *sounding*) (Schroeder et al. 2015). Reflection and transmission losses are quantified using scattering models applicable to wavelength-scale roughness (Fung et al. 1992) and are not scaled across the Fresnel zone or pulse-limited footprint.

Data constraining the roughness of Europa’s surface are sparse. High resolution Galileo images of Europa are rare, and regions of good stereo coverage are even rarer. Although Europa’s surface is complex, assessments of DTMs tend to show that there are two dominant roughness regimes (Nimmo and Schenk 2008; Steinbrügge et al. 2020b): (i) Ridged Plains (Prockter and Patterson 2009) and (ii) Chaotic Terrain (Collins and Nimmo 2009). Estimates for the self-affine structure of Europa’s surface based on the DTMs of Nimmo and Schenk (2008) (see Table 1 in Steinbrügge et al. (2018)) allowed the roughness estimates to be scaled to REASON radar wavelengths, yielding the values in Table 4.

#### Near-Surface Density, Composition, and Thickness

Europa’s shell is dominated by water ice. The density of the ice will affect the real part of the permittivity and thus the reflection coefficient of the near-surface. We use 910 kg m^−3^ to represent nonporous water ice and 518 kg m^−3^ for porous water ice. The latter mode corresponds to the limit of random loose packing for uniform spheres (Onoda and Liniger 1990) and is adopted to represent the case of a relatively porous ice using a rigidity percolation threshold, since observational constraints of the porosity of the near-surface are lacking. Note that these densities are used to represent porosity and as such their magnitudes are not necessarily comparable to the density of ice expected at Europa, which could exceed 930 kg m^−3^ for nonporous ice at surface temperatures (Carnahan et al. 2021). Also note that the density imposed for the near-surface does not describe the porosity at depth. The porosity at depth is defined by the deep regolith regime (Sect. 4.2.3), which governs total integrated volume-scattering losses.

We define two modes for near-surface composition: (i) clean ice ($\varepsilon = 3.15$) and (ii) brine-filled ice ($\varepsilon = 9$). The permittivity for brine-filled ice corresponds to an empirically-determined value for the near-surface layer of brine-saturated firn of the McMurdo Ice Shelf, Antarctica, calculated from the brine zone reflectance of −6 dB shown in Figure S3 of Grima et al. (2016). Brine-saturated firn in Antarctica represents an analog for brine-filled ice that could be present in the near-surface of Europa (Schmidt et al. 2011). Although such brines would be transient in nature and not expected to remain liquid under near-surface Europan conditions (Vu et al. 2016; Thomas et al. 2017), their detection would be scientifically relevant for a potential future lander mission and could represent a potential habitat (Pappalardo et al. 2013; Wolfenbarger et al. 2022c). The presence of fossil brines (i.e., salt hydrates) is not represented here since the influence of porosity on the near-surface reflection coefficient dominates over the presence of impurities, except when liquid brine is present.

The near-surface thickness can influence the reflection coefficient if the layer thickness is on the order of wavelength scale (Lalich et al. 2022; Scanlan et al. 2022; Lauro et al. 2023). Incident and reflected waves within a thin layer can interfere constructively or destructively, depending on the permittivity and thickness of the thin layer (Mouginot et al. 2009). We define two modes for the thickness of the near-surface layer: (i) 0.5 m and (ii) 200 m. The 0.5-m surface layer mode is defined from observations of Europa’s surface using S-band Earth-based radar (Black et al. 2001), consistent with modeled estimates of the gardened layer thickness (Costello et al. 2021). The 200 m surface layer mode corresponds to the thickness equal to the height of a typical Europan ridge (Head et al. 1999).

#### Deep Regolith

The presence of volume-scatterers within the ice shell, referred to here as deep regolith, could influence the radar signal penetration depth. An early study of volume scattering losses found that regolith at depth would pose an “insurmountable obstacle” to radar sounding of Europa’s ice shell (Eluszkiewicz 2004). A follow-on study determined these estimates were “unduly pessimistic” and found that much of the range of estimated volume scattering losses due to regolith at depth are minor at Europa for both HF and VHF frequencies (Aglyamov et al. 2017). They found large losses for the VHF at high porosities (>10%) when void size was large (>22 cm); however, losses were insignificant for the HF. These extreme values are consistent with the porosity needed to generate observed positive band topography in the case of a thin ice shell (Nimmo et al. 2003). We adopt two modes to represent volume scattering losses due to the presence of deep regolith: (i) a 22% porosity regolith with 22 cm pore radius, which correspond to upper bounds of the parameter space defining regolith porosity and size considered in Aglyamov et al. (2017), and (ii) nonporous ice. The upper bound in regolith loss is intended to represent conditions where the VHF band is unable to make *sounding* measurements and as such represents conditions that could be considered extreme relative to other models for the porosity of Europa’s ice shell at depth.

#### Surface Temperature

Surface temperatures affect the equilibrium conductive ice shell temperature profile, which in turn affects the attenuation. As Fig. 10 demonstrates, higher ice shell temperatures result in higher signal attenuation. To first order, Europa’s surface temperature is driven by latitude. We adopt estimates for the mean annual equatorial and polar surface temperatures, consistent with those used in previous models for radar attenuation in Europa’s ice shell (Chyba et al. 1998; Moore 2000). Values are consistent with those obtained from numerical thermal models of Europa’s ice shell that account for moderate internal heat flow and second order influences related to orbital geometry (Ashkenazy 2019). Unlike other regimes, point models are evaluated for both surface temperature modes since observations span multiple latitudes over a flyby. Polar surface temperatures translate to a less attenuating ice column and thus more optimistic performance. Although an equatorial temperature represents the conservative endmember from the perspective of radar signal attenuation, we evaluate both surface temperatures to ensure that the influence of latitude on success or failure of a point model is tracked.

#### Ice Shell Composition

Bulk ice shell composition is represented by two regime modes: (i) pure ice and (ii) marine ice. These modes represent the impurity contribution to the high-frequency electrical conductivity of ice, which governs radar signal attenuation in ice (Moore 2000; MacGregor et al. 2007, 2015). Note that only lattice-soluble impurities (impurities that are incorporated within the ice crystal lattice and not accommodated interstitially) contribute to the high-frequency electrical conductivity of ice. On Europa, chloride is thought to be the most ubiquitous and dominant lattice-soluble impurity (Grimm and Stillman 2019; Moore 2000), incorporated through entertainment of oceanic material at the ice–ocean interface (Buffo et al. 2020; Wolfenbarger et al. 2022a). Note that sulfate, which has been predicted to be a dominant constituent of Europa’s ocean (Kargel 1991; Zolotov and Shock 2001), is not lattice-soluble in ice (Pettinelli et al. 2016; Grimm and Stillman 2019; Moore 2000). Similarly, radiolytically generated sulfuric acid (Carlson et al. 1999, 2002), concentrated along Europa’s trailing hemisphere (Fischer et al. 2015; Ligier et al. 2016; Trumbo et al. 2020), is not expected at depth in the ice column. Impactor material is not represented as an ice shell impurity because it is not expected to contribute to the electrical conductivity or be present in high enough volume fraction to contribute to volume-scattering losses, with the possible exception of impact crater ejecta (Schenk and McKinnon 1991; Tomlinson and Hayne 2022). The electrical properties of ice used to model the attenuation rate in Fig. 10 are adopted from Moore et al. (1994) and are comparable with values typically used in radioglaciology (MacGregor et al. 2007, 2015). The marine ice chlorinity mode (60 $\mu $M) was derived from the modeled Europa K1a ocean composition of Zolotov and Shock (2001) and the equilibrium solute distribution coefficient of Gross et al. (1977).

#### Ice Shell Thickness

There has been considerable controversy in estimating the thickness of Europa’s ice shell. A full discussion on estimates for the thickness of Europa’s ice shell can be found in Howell (2021), Nimmo and Manga (2009), and Billings and Kattenhorn (2005). From the perspective of *sounding* in the REASON investigation, we are interested in the thickness of the cold, thermally conductive lid, where the relatively low temperature results in minimal radar signal attenuation (Fig. 10). This may also include cold downwellings associated with a slowly convecting ice layer below the thermally conductive lid (Kalousová et al. 2017). We consider two modes for ice shell thickness: (i) a thin ice layer extending to 3 km depth, and (ii) a thick ice layer extending to 30 km depth. The thin ice layer mode represents multiple hypotheses including: a thin global ice shell directly over an ocean — hypothesized based on chaos block elevations and crack geometry (Greenberg et al. 1999), local regions of thin ice over water lenses within a thicker ice shell (Schmidt et al. 2011), and a thin conductive layer over convecting ice (Mitri and Showman 2005). The thick conductive layer accommodates upper bound estimates from mechanical models, impact cratering analyses, and thermodynamic analyses (Billings and Kattenhorn 2005) and is compatible with the current best estimate of 10.4 km obtained by Howell (2021).

#### Salt Layer Thickness and Depth

Salt layers represent a form of possible structure within the ice shell, and thus a potential target interface. Recent works have modeled the formation of salt layers from the freezing of ocean-injected sills and found salt layers on the order of meters thick (Buffo et al. 2020; Chivers et al. 2021). Here we consider two processes for the formation of a sill: (i) the generation of melt from native ice shell material (Schmidt et al. 2011) and (ii) injection from a subsurface ocean (Michaut and Manga 2014). Because the bulk ice shell salinity is likely to be over an order of magnitude less saline than the ocean (Buffo et al. 2020; Wolfenbarger et al. 2022a), a salt layer formed from melt is likely to be an order of magnitude thinner than one formed from ocean water (e.g., 0.2 m vs. 1 m, respectively). We adopt $\varepsilon = 5$ to represent the permittivity of the hydrated salt layer (Grimm et al. 2008; Pettinelli et al. 2016; Grimm and Stillman 2019). We position salt layers formed from ice shell melt 10 km from the surface, consistent with a maximum measured topographic relief (≅6 km) and inferred freeboard (≅4 km) over Thera Macula (Schmidt et al. 2011). We position salt layers formed from ocean water halfway through the ice shell, consistent with estimates for the base of an elastic layer within an ice shell, thought to be important for sill emplacement (Michaut and Manga 2014).

#### Subsurface Water Interface

Two modes are used for determining the reflection coefficient associated with a subsurface water interface: (i) water and (ii) mush. The water mode represents flat, pure ice over terrestrial seawater (Peters et al. 2005b). The mush mode represents flat, pure ice over a 30% ice-water mixture. The latter mode is intended to represent the reflection coefficient from a eutectic interface within the ice column, consistent with the sharp interface in Culha et al. (2020). Physically, the mush case represents the transition across the eutectic temperature, where melt becomes stable (Zolotov and Shock 2001; Zolotov and Kargel 2009; Wolfenbarger et al. 2022b). We do not include a model for a gradient in liquid volume fraction. REASON is not designed to penetrate an increasingly mushy brine layer, which would be highly electrically conductive and thus attenuating to radar (Stillman et al. 2018). Although penetration of a mushy layer is unlikely, the properties that cause the layer to be high loss in turn make the eutectic interface (the top of the mushy layer) more reflective and thus easier to detect. The eutectic interface represents a proxy for an ice–ocean interface allowing for total ice shell thickness and thermophysical structure to be constrained.

#### Basal Temperature

We use two modes for the basal temperature: (i) the predicted melting point of pure water ice, which is approximately 270 K beneath a range of hypothesized ice shell thicknesses on Europa (Melosh et al. 2004), and (ii) the eutectic temperature of aqueous NaCl, which is approximately 250 K. Changes in basal temperature influence the temperature profile within the ice column, particularly near the basal interface. The temperature profile directly influences the radar signal attenuation in ice, given the nonlinear temperature dependence of radar signal attenuation in ice.

#### Coupled Regimes

Certain regimes are coupled, either due to physical constraints or to represent specific hypotheses. The character of a subsurface water interface is thermodynamically constrained and as such is coupled to the basal temperature. Because we define mush as a eutectic boundary and consider salt to be the only non-ice impurity, we couple this mode to a basal temperature of 250 K, which is consistent with the eutectic temperature of an aqueous NaCl solution. A pure ice–water interface is only thermodynamically stable at the melting temperature, and as such this mode is coupled to a basal temperature of 270 K (i.e., the approximate melting temperature for pure ice). Since diapirism is considered to be the mechanism for generating melt in a thick ice shell, a 0.2-m-thick salt layer is only considered stable for a thick ice layer. The 1-m-thick salt layers can exist in both the thick and thin ice layer cases, since the formation mechanism for the sill involves fracture and injection which could occur in either a thin shell or thick shell case.

### Ensembles of Point Models (EPMs)

There are four EPMs used to V&V REASON requirements on measurement quality for *sounding*, *reflectometry*, and *altimetry* (Table 5). Note that the requirement for *interferometry* is verified through evaluation of the interfero-metric^11^ KRP (Sect. 4.4.7).

**Table 5:** EPMs used to evaluate REASON requirements on measurement quality

EPM	Measurements	Datasets	Altitudes
Shallow Subsurface Structure	Sounding	Shallow Subsurface	≤400 km
Full Depth Subsurface Exchange	Sounding	Full Depth Subsurface Exchange	≤400 km
Ice Shell Properties	Sounding	Ice–Ocean Interface	≤400 km (VHF)
			≤1000 km (HF)
Altimetry and Reflectometry	Altimetry and Reflectometry	Shallow Subsurface	≤400 km
		Full Depth Subsurface Exchange	
		Near-Surface Properties	
		Ice–Ocean Interface	≤1000 km

**Fig. 12:**
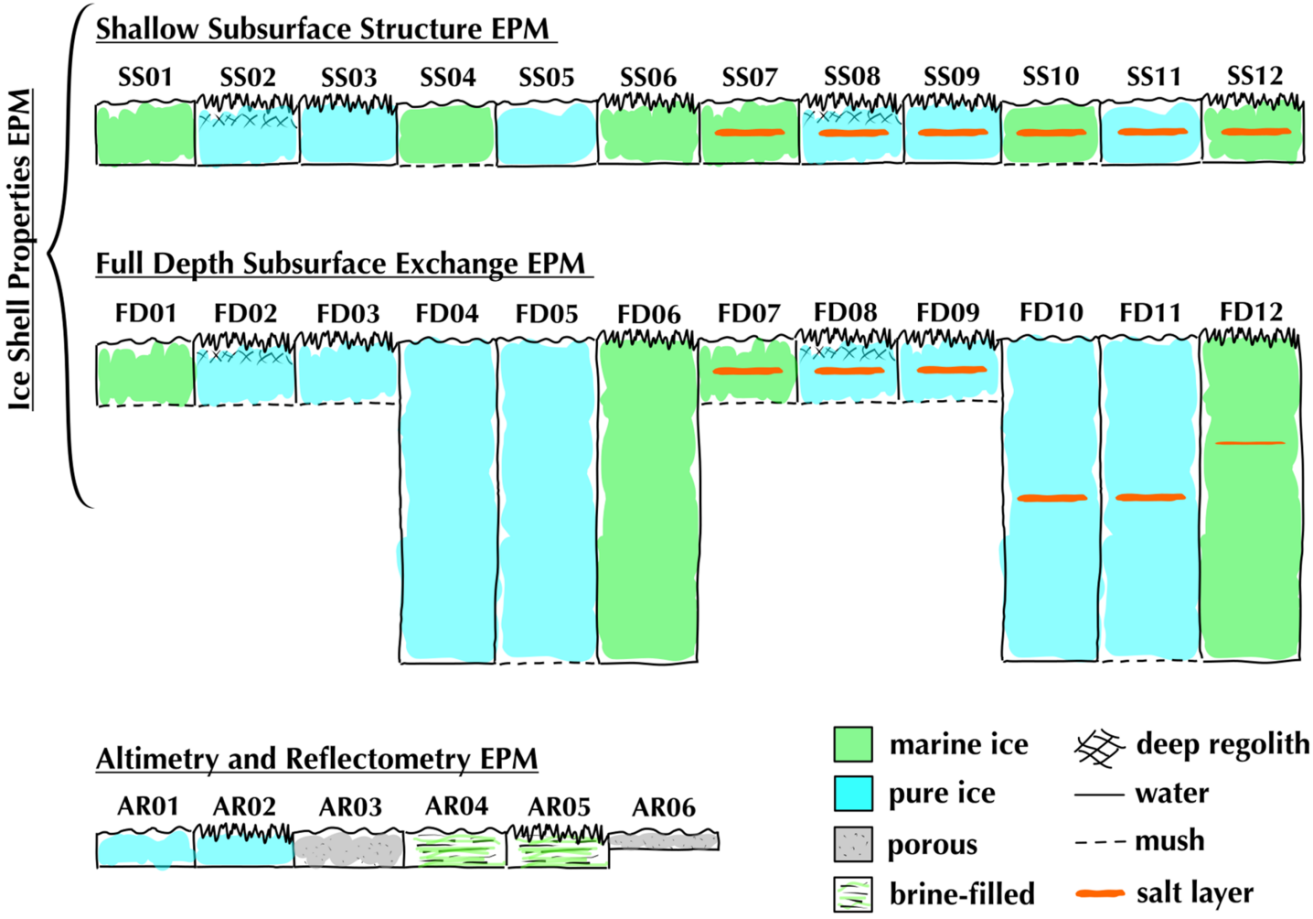
EPMs used to represent Europa for evaluating Measurement Quality Requirements

These EPMs are represented graphically in Fig. 12 and detailed in Tables 12–14 in the Appendix.

Each point model in the EPM is intended to represent a specific hypothesis, considered by the REASON team as part of instrument development. The point models in the Shallow Subsurface Structure EPM are represented in the Full Depth Subsurface Exchange EPM but include modifications to either the basal temperature and/or the ice shell thickness. In the Shallow Subsurface Structure and Full Depth Subsurface Exchange EPMs, the last six point models are the same as the first six but include salt layers as the target interface, as opposed to an ice–water interface.

A requirement on measurement quality is considered verified and validated if the measurement is successful for a majority of the point models in an EPM given performance derived from KRPs/instrument-level requirements. EPMs are used to verify and validate that the requirements on measurement quality are capable of “characterizing” and “searching” where applicable (Table 1). Where a Measurement Requirement traces up to a dataset that involves “characterizing” or “mapping” a property of Europa, the requirement must be met for both a subsurface structure target, represented by salt layers, and a subsurface water target interface. Where a Measurement Requirement traces up to a dataset that involves searching for features at Europa, we consider the requirement met for either a subsurface structure or subsurface water target interface.

### Key Radar Parameters (KRPs)

The KRPs are supplied by the REASON System Engineering team to the REASON science verification and validation (SVV) team. KRPs translate estimated instrument performance (including spacecraft-level impingements) and are used as an input for REASON SVV. Current Best Estimate (CBE) values are used to verify requirements on measurement quality, whereas the values derived from instrument-level requirements are used for validation.

Not all KRPs are needed to evaluate a particular requirement on measurement quality. Table 6 presents the types of requirements on measurement quality evaluated in REASON SVV and the KRPs that contribute to their verification and validation. Radar potential (Sect. 4.4.1) and SAR gain (Sect. 4.4.4) are included in the evaluation of all requirements. Analysis of the center frequency uncertainty (Sect. 4.4.2) is used to justify the assumption in SVV that the center frequency corresponds to the required value. Vertical resolution (Sect. 4.4.3) is converted to an effective bandwidth in SVV and used to evaluate all vertical resolution and vertical precision requirements. The Normalized Side Lobe Envelope (NSLE) (Sect. 4.4.5) is used to evaluate all requirements that apply to the subsurface.

**Table 6:** Key Radar Parameters (KRPs) used to verify REASON requirements on measurement quality

		Key Radar Parameters (KRPs)						
		Radar Potential	Center Frequency Uncertainty	Vertical Resolution	SAR Gain	NSLE	Radiometric Stability	Interfero-metric
Requirements on Measurement Quality	Blind Zone (3.2.1.)	×		×	×	×		
	Vertical Resolution (3.2.2.)	×		×	×	×		
	Vertical Precision (3.2.2.)	×	×	×	×	×		
	Along-Track Resolution (3.2.3.)	×			×	×		
	Sampled Along-Track Resolution (3.2.4.)	×			×			
	Clutter Discrimination (3.2.5.)	×						×
	Radiometric Precision (3.2.6.)	×			×		×	
	Radiometric Accuracy (3.2.6.)	×			×		×	
	Radiometric Stability (3.2.7.)	×			×	×	×	

#### Radar Potential

Radar potential is defined as the SNR of a flat, perfect, infinite reflector at a range of 400 km, with no environmental perturbations (e.g., neglecting the ionosphere). Radar potential includes terms for the gain resulting from SAR processing as well as impacts from instrumental effects and capabilities.

#### Center Frequency Uncertainty

Center frequency uncertainty quantifies the variation in center frequency expected over the mission. Uncertainty in the center frequency represents uncertainty in the STAble Local Oscillator (STALO) clock, and thus the uncertainty in measuring the time between chirp transmission and echo receive, which can feed into *altimetry* and *ranging* errors.

#### Vertical Resolution

Vertical resolution in vacuum is an instrument-level requirement and can be used to calculate an effective bandwidth. It is taken as the 3 dB width of the main peak lobe in vacuum after range compression. A Hann window is used to reduce the impact of the range side lobes (see the NSLE below in Sect. 4.4.5), but results in broadening of the peak lobe and loss of resolution. In operational processing, other windowing functions may be used.

#### SAR Gain

SAR Gain is the gain obtained from focused SAR processing at 400 km, assuming an aperture length equal to the Fresnel zone radius. The scatterer is assumed to be a specular (smooth/flat) surface at nadir. Reflectometry measurements do not benefit from SAR gain since the technique depends on analysis of the returns with minimal azimuth processing.

#### Normalized Side Lobe Envelope (NSLE)

**Fig. 13:**
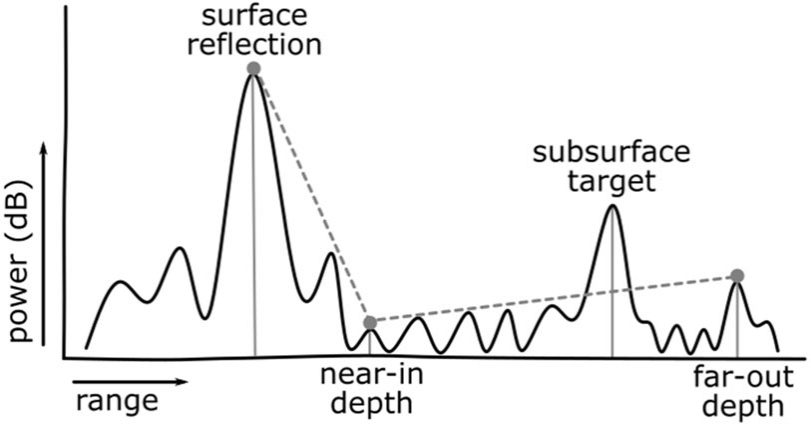
NSLE

The NSLE is used to describe artifacts generated during the range compression of the surface echo and is illustrated in Fig. 13. In order to minimize side lobe power levels, range compression of the reflected chirps is typically performed using a windowed version of the reference chirp, here assumed to be a Hann window. Range compression with a windowed reference chirp leads to a steep decrease in the power of undesirable side lobes on either side of the surface echo that eventually reaches a minimum at some range (i.e., the “near-in” position). At ranges beyond the near-in position, side lobe power levels will begin to increase and eventually reach a local maximum (i.e., the “far-out” position). The NSLE KRP describes the range and relative power of the near-in minimum side lobe power as well as the far-out local maximum. The near-in portion of the NSLE KRP is used in the analysis of the REASON blind zone requirement while the far-out, if above the noise floor, is used to calculate a reduction in the SNR of echoes from subsurface targets. The absolute power levels associated with the near-in and far-out NSLE KRP are scaled relative to the strength of the surface echo. The near-in and far-out formulation is only relevant for cases with modest electromagnetic interference (EMI) and radio frequency interference (RFI).

#### Radiometric Stability

The radiometric stability KRP quantifies the expected variation in power across a flyby caused by temperature variations, calibration errors, and other instrument-related phenomena. The radiometric stability KRP includes metrics for both a radiometric precision (short term) and radiometric accuracy (over a flyby). The radiometric stability KRP is used to evaluate requirements related to radiometric stability, precision, and accuracy. There are no radiometric stability KRPs or requirements for longer periods. The radiometric stability KRPs do not include imaging geometry effects.

#### Interfero-Metric

Unlike the other KRPs, the interfero-metric includes geophysical effects. The interfero-metric KRP quantifies whether *interferometry* is successful for a given point model at a specific altitude. The point models used in generating the interfero-metric include all those in the Shallow Subsurface Structure and Full Depth Subsurface Exchange EPMs that have target depths less than or equal to 3 km. For point models with salt layers, the salt layer depth is swept from the blind zone down to 3 km in 10-m increments. To assess whether *interferometry* is successful, both detection and discrimination must be evaluated. For each point model, the SNR and interferometric phase of both the target and the clutter source is calculated. The central assumptions for the interfero-metric are that a subsurface target or surface target is present, but never both, and that the subsurface target has a 10 km extent along-track. A feature is considered “detected” if its SNR is above a certain threshold, defined as 0 dB, consistent with the detection criteria for evaluation of other REASON Measurement Quality Requirements. A feature is considered “discriminated” if the expected value of its interferometric phase can reliably be determined to be below or above a given threshold. The roughness of either the nadir or off-nadir extended target will cause a spreading of the phase of the backscattered echo. As such, the interferometric phase can be described by a statistical distribution. Because of this, statistical criteria are used to establish whether the interferometric phase is above or below the threshold.

## Instrument Design

### Description of Radar Functions, Architecture and Operation

The radar consists of three stacks which reside inside the spacecraft vault: Digital and Synthesizer Electronics Stacks (DSES) that includes two redundant Digital and Synthesizer Electronics (DSE-A and DSE-B) units with cross-strapped interfaces to the Radio Frequency (RF) electronics. This allows for radar operation during the mission to be tolerant to single DSE faults. Each DSE includes a Frequency Synthesizer and Digital and Power Unit (DPU). The DPU consists of a Radar Acquisition & Management Board (RAMBo) and a DSE Power Converter Unit (DSEPCU) that powers the RAMBo and the Synthesizer. The RF Electronics System (RFES) for REASON is divided into HF and VHF Stacks. Each stack includes the receive and transmit electronics.

**Fig. 14:**
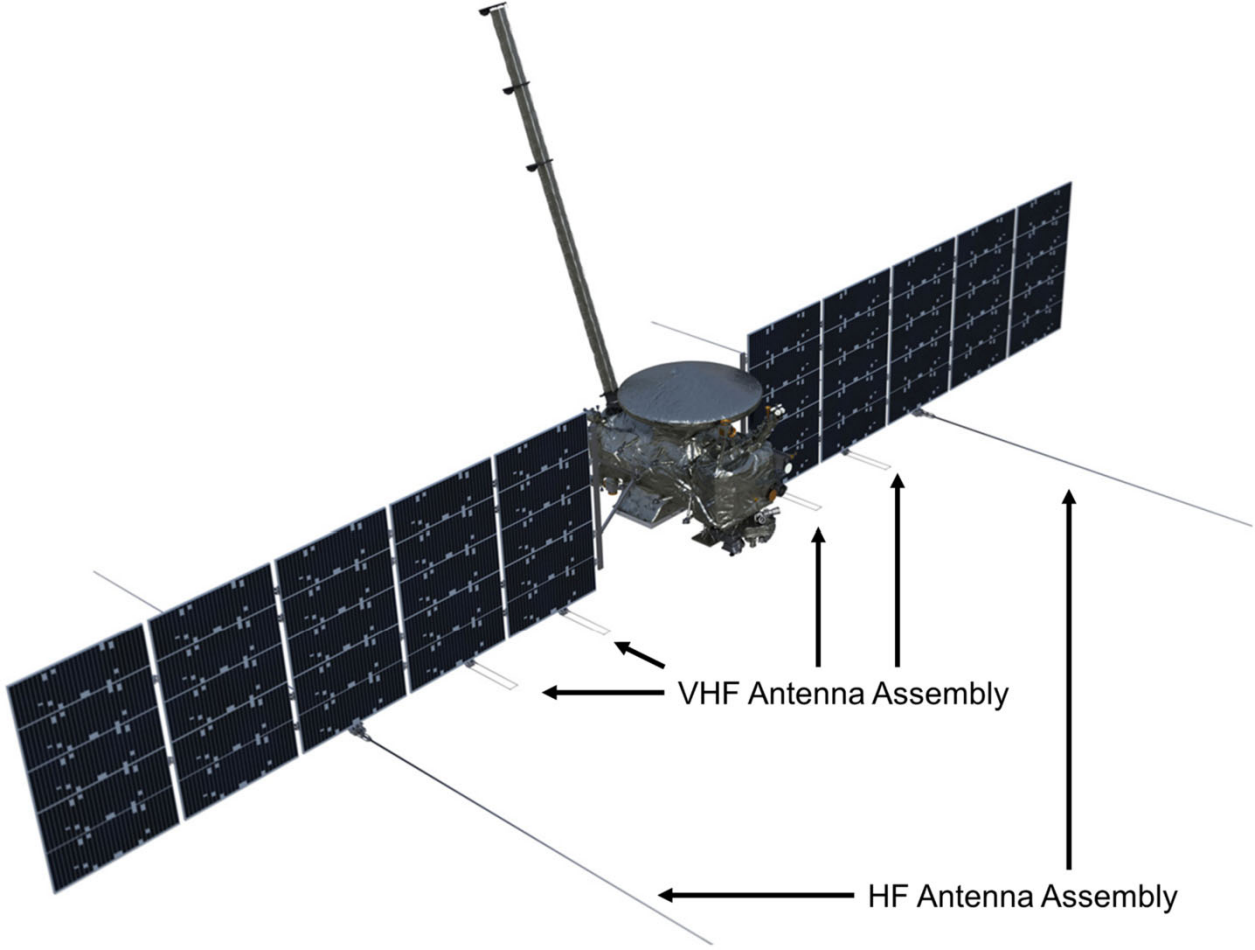
Depiction of Europa Clipper spacecraft with REASON antenna assemblies

The Antenna Subsystem (AS) is located outside of the vault and is mounted on the Solar Arrays (SA). The AS is an integrated array including two 17.6-m HF dipole antenna assemblies and four 2.76-m VHF folded dipole antenna assemblies. Each antenna assembly in the array contains a radiating element and an impedance Matching Network (MN) to transform the RF front-end nominal 50-ohm characteristic impedance to the impedance of each radiating element. The antenna assemblies are designated with “+X” (Starboard side) or “–X” (Port side), as well as “inner” or “outer” for the VHF, to identify their location on either solar array wing. Coaxial cable assemblies, both semi-rigid and flexible, are used to connect the antenna assemblies to the vault electronics. The antenna assemblies are scheduled for deployment roughly one month after launch. A rendering of the Europa Clipper spacecraft with REASON’s AS is shown in Fig. 14.

**Fig. 15:**
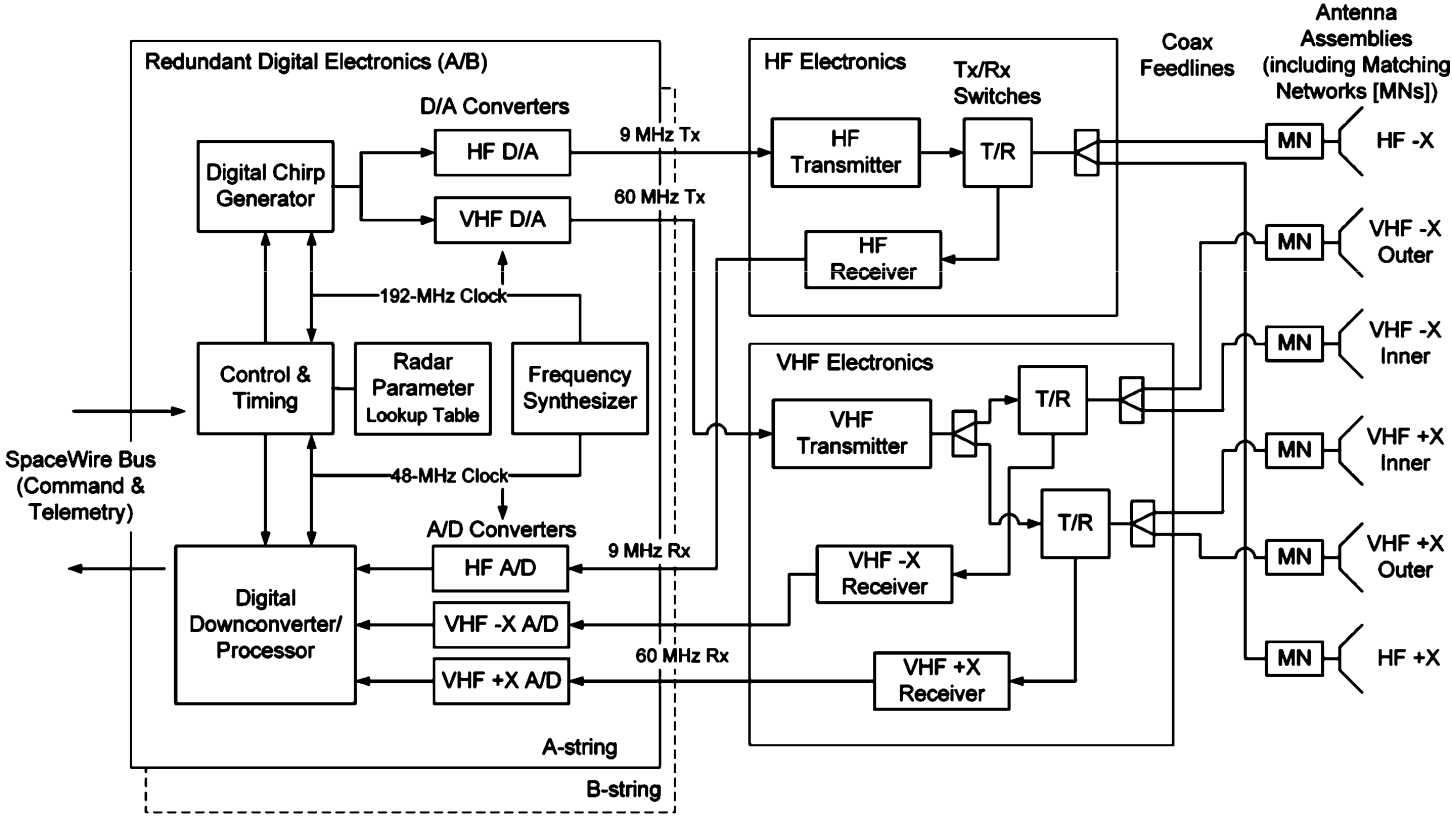
REASON functional block diagram

Figure 15 shows the functional block diagram of the REASON 9- and 60-MHz ice-penetrating radar, showing the frequency plan and transmit-receive signal flow. Coherent radar signals are derived from: 1) a 48-MHz STALO for synchronizing radar control-and-timing, analog-to-digital conversion, onboard processing and data compression of received echoes; and 2) a frequency-multiplied 192-MHz clock for the digital chirp generation and digital-to-analog conversion of transmit pulses. Given the relatively low HF and VHF frequencies involved, all transmit frequency generation and receive downconversion is done completely in the digital electronics, without the need for upconversion or downconversion in the RF section. The digital electronics design uses a single, reconfigurable field-programmable gate array (FPGA). The FPGA firmware architecture is state-machine-based and does not require a flight computer module or flight software. The radar Control and Timing Unit (CTU) firmware component orchestrates the measurement sequence during a Europa flyby through a programmable Observation Plan (OP) script file stored in the RAMBo’s Static Random Access Memory (SRAM). For a given flyby, the spacecraft uploads the OP contents to SRAM and initiates science data collection via a SpaceWire command. When data collection starts during the approach, the CTU plays out a deterministic sequence of radar transmit and receive events as defined by the OP, with radar parameters updated once every few-second dwell interval. The FPGA processes, assembles, and packetizes REASON science data and engineering telemetry and sends these data back to the spacecraft via SpaceWire for downlink.

**Fig. 16:**
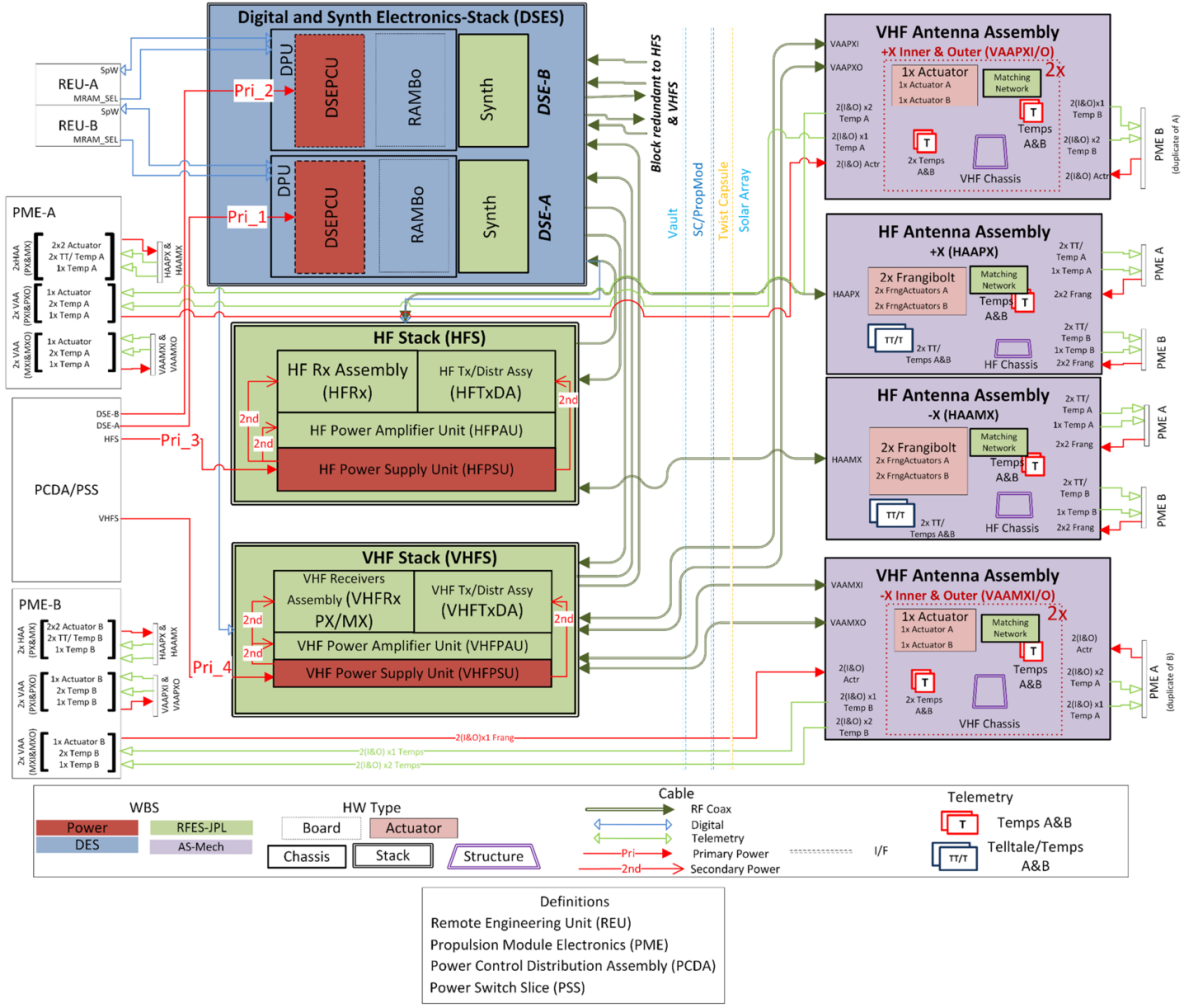
REASON implementation block diagram

The RF front-end architecture for HF and VHF electronics stacks (HFS, VHFS) uses a high-efficiency Gallium Nitride (GaN) power amplifier plus Transmit/Receive (T/R) switches with coupled transmit-loopback calibration signal injection back into each of three receiver channels (single-channel HF and dual-channel interferometric “+X/–X” VHF). Loopback provides a calibration reference to compensate in post-processing for any RF transmitter/receiver amplifier gain variations over the flyby collection period. T/R pulses are distributed through front-end power combiners-dividers, through a network of six coaxial feedlines to the two HF and four VHF antenna assemblies. A summary of radar system parameters is shown in Table 7, and a block diagram of the overall REASON architecture is shown in Fig. 16.

**Table 7:** REASON radar system parameters (actuals)

	HF	VHF
Peak Transmit Power at input to the antennas, per antenna assembly	11.75 W	5.5 W
Center Frequency	9 MHz	60 MHz
Bandwidth (Fractional Bandwidth)	1 MHz (11%)	10 MHz (17%)
Operational Altitudes	25 km to 1000 km from Europa	
Pulse Repetition Frequency (PRF)	50 Hz–3 kHz	
Approximate On-time per Orbit	20 min warm-up and 16 min science collection	
Dynamic Range	40 dB*	35 dB*
Antenna Gain at Nadir	4 dBi*	7.3 dBi
Mass	65.7 kg	
AveragePower	61 W	

*Requirement or CBE at time of publication.

#### Jovian Environment as a Design Driver for REASON

A major driver for REASON’s design was the challenging Jovian radiation environment (Kim et al. 2019). Parts and materials were selected with total ionizing dose (TID) in mind such that REASON will survive and perform throughout the mission design life. Internal electrostatic discharges (IESD), caused by dielectric materials accumulating charge in the environment and then discharging, were also an important consideration. IESD poses both a damage risk and a data quality risk to REASON, and both effects were factored into the design choices for REASON. In general, all non-metallic materials were either tested or analytically assessed for their suitability for use in the expected radiation environment. Electron beam testing was conducted at either the Jet Propulsion Laboratory (JPL) Dynamitron (Pasadena, CA) or at the Goddard Space Flight Center (GSFC) Radiation Effects Facility (Greenbelt, MD).

In order for REASON to survive and avoid damage from the worst-case IESD event, which could either be generated by the instrument itself or by another part of the spacecraft and coupled into the instrument via the antennas, dielectric materials had to be carefully selected, particularly in the antenna assemblies, as they are located on the solar arrays and relatively exposed to the Jovian environment (Sect. 5.2.3). Furthermore, REASON needed to be hardened to the worst-case potential discharges that it might experience during the mission. To this end, the MNs underwent a thorough qualification campaign to demonstrate that they will be able to survive a 1-kV, worst-case event.

Additionally, REASON is sensitive to low-amplitude IESD events that would not otherwise pose a damage risk to electronic hardware. A high rate of these events has the potential to affect radar performance and effectively inject noise into the science data collected. REASON has determined that it can tolerate a 5% data loss; this corresponds to 436 IESD events per second. Discharges from both within REASON as well as the entirety of the spacecraft must be accounted for, and a total bounding discharge rate across the spacecraft is bookkept to ensure that it will be tolerable for REASON’s performance. This was also an important consideration in the materials selection for REASON.

### Radar Subsystems

#### Digital Electronics Subsystem

The REASON Digital Electronics Subsystem (DES) consists of three main boards housed in a common chassis: the DSEPCU, the synthesizer, and the RAMBo. As described previously, there is an A- and B-side for DSE redundancy. In addition to interfacing with the spacecraft, the primary purpose of the REASON digital subsystem is to coherently transmit chirps, receive the echoes, and reduce the receive data rate and data volume, while preserving the SNR necessary for science. The radar’s operation is controlled by the OP sequence file. The OP defines when the radar transmits chirps, when it collects receive data (transmit loopback or receive echoes) and configures the receive data processing. An OP is constructed on the ground, with operating parameters determined by the flyby trajectory, science goals, SpaceWire maximum data rate, and spacecraft storage constraints. The radar’s CTU processes the OP to generate transmit signals to the RF interfaces, trigger collection of receive echoes, and send processing parameters to the on-board processor (OBP). The OBP performs digital downconversion, filtering, and decimation of the receive echoes, reducing the sampling rate from 48 MHz at the analog-to-digital converters (ADCs) to complex-valued baseband sampling rates of 1.2 MHz for HF and 12 MHz for VHF. The receive data are optionally coherently summed (to reduce the data rate); the number of coherently summed echoes is commandable up to 255 for transmit loopback or up to 40 for receive echoes. Coherent summing is enabled by round-trip delay tracking logic that aligns receive echoes to within a fraction of a sample. The coherently summed echoes are then compressed using Block Floating-Point Quantization (BFPQ), which is similar to quantization algorithms used in previous planetary missions (Kwok and Johnson 1989). It is a vector quantizer that generates an 8-bit exponent code (power estimate) for a block of samples, and lower bit-width mantissa codes for each complex-valued baseband sample within the block. The BFPQ block size is programmable, as is the number of bits per mantissa sample. As an example of the data compression performance, the BFPQ input data rate for VHF is 576 Mbps $(12\text{ MHz} \times (24+24))$, while the output data rate for a block size of 32 and 4-bit mantissa codes is 99 Mbps $(12\text{ MHz} \times (32 \times (4+4) + 8)/32)$, i.e., a reduction of around six times, while maintaining a signal-to-quantization noise ratio of over 20 dB. During the flyby, the mantissa bit-width is adjusted to capture the expected SNR, i.e., lower bit-widths at higher altitudes, and higher bit-widths at lower altitudes. During a flyby the radar digital subsystem generates hundreds of science packets per second (for about a dozen unique science packet types), along with health and status and engineering packets once per second. A summary of DES parameters is shown in Table 8.

**Table 8:** DES parameters

Parameter	HF	VHF
ADC sampling rate	48 MHz	
Complex-valued baseband sampling rate	1.2 MHz	12 MHz
Downconverter center frequency	9 MHz	12 MHz
ADC bit resolution	9-10 bits (effective)	

##### Digital and Synthesizer Electronics Stacks (DSES)

Within each DSE, the synthesizer generates the 48 MHz and 192 MHz clocks used by the radar and was built by Wenzel Associates (Austin, TX). The RAMBo was built by L3 Harris (Mason, OH) and utilizes the Xilinx Virtex-5QV radiation-hardened FPGA. The digital-to-RF transmit interface is implemented using a 4-bit, 384 MHz sample rate (192 MHz double-data-rate) Digital-to-Analog converter (DAC) R-2R resistive ladder network, with separate interfaces for HF and VHF chirp generation. The RF-to-digital receive interface is implemented using three STMicroelectronics RHF1201 12-bit ADCs operated at 48 MHz to sample the HF, VHF+X, and VHF-X receiver outputs. The Spacecraft interface is implemented using a 192 Mbps SpaceWire interface, and a low-voltage differential signaling (LVDS) discrete input that controls the FPGA configuration source. The FPGA configures from one of two Honeywell 64 Mbit magnetoresistive random-access memories (MRAMs). The current plan is for both MRAMs to launch with identical configurations: one MRAM is read-only, while the other can be updated post-launch. The RAMBo also contains a telemetry ADC, analog multiplexers, current sources, and buffer amplifiers for measuring voltages, currents, and temperatures from the RFES, DSEPCU, synthesizer, and itself. Telemetry is averaged and reported once per second.

##### Digital and Synthesizer Electronics Power Converter Unit (DSEPCU)

The DSEPCU converts the spacecraft voltage into six voltage outputs (isolated from the spacecraft bus) to the Synthesizer and RAMBo. It shares a chassis with the RAMBo and interfaces with it directly via an HMM connector. The DSEPCU contains standard housekeeping functions such as an undervoltage lockout, voltage and current telemetry of its outputs, and an EMI filter. It also mitigates concerns that come with a high radiation environment. The 300 krad TID environment drove the design of the flyback and buck converters due to the limited selection of pulse-width modulation (PWM) controllers at this dose. The DSEPCU includes a remote sense for the 1-V rail to increase the voltage accuracy delivered to the RAMBo by compensating for the voltage drop along the high-current path. It also contains a dual flyback output that shares a feedback network. A key function of the DSEPCU is to provide power sequencing. To ensure the proper powering off and on, digital logic is used. Additionally, discharge circuits and robust energy storage are used to guarantee the power off sequencing under worst case loading.

##### Synthesizer Assembly

The Synthesizer Assembly (Synth) generates 48 MHz and 192 MHz clock signals from a single 48 MHz temperature-stabilized oscillator circuit. The oven maintains the temperature of the crystal at a fixed temperature to provide excellent phase noise and frequency accuracy performance over the entire flight temperature range. In addition to providing temperature telemetry, the Synth provides an oven status indicator to the DPU that is used to monitor the health of the Synth.

#### Radio Frequency Electronics Subsystem (RFES)

**Fig. 17:**
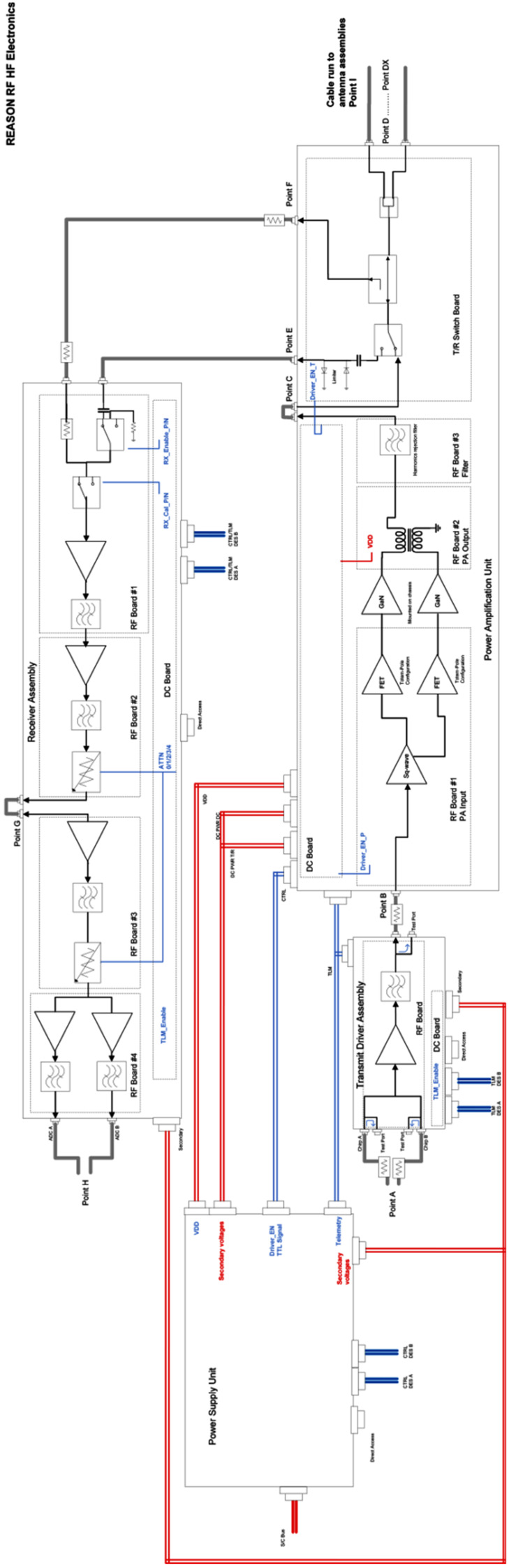
HF RF In-vault block diagram

**Fig. 18:**
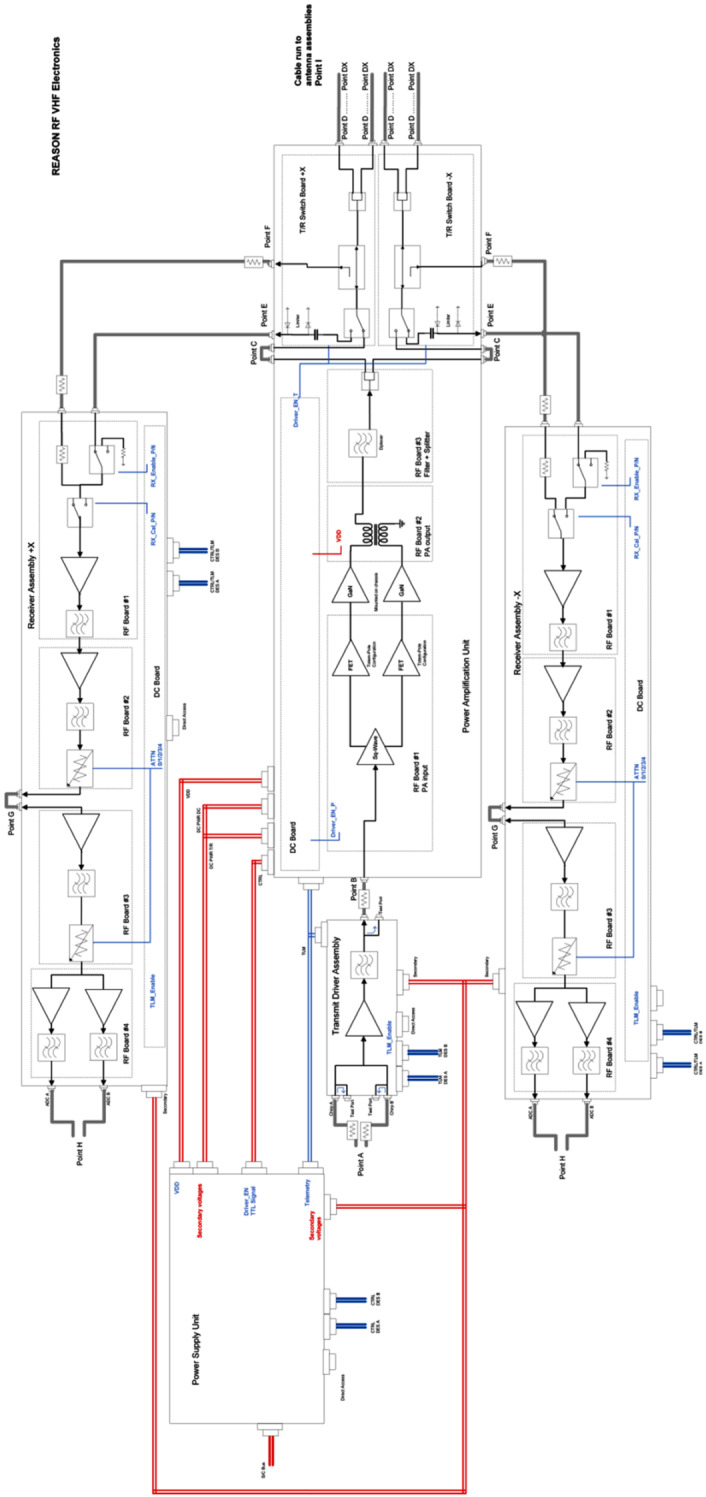
VHF RF In-vault block diagram

The RFES includes the HF and VHF electronics stacks. Figures 17 and 18 show block diagrams for the HFS and VHFS, and the key requirements for the RFES are listed in Table 9. The electronics for the two frequencies are independent to increase the fault tolerance of the system. Each RF electronics stack consists of a Transmit Driver Assembly (TxDA), a Power Amplification Unit (PAU), Receiver (Rx) and an RF Power Supply Unit (RFPSU). The HF Stack consists of one receiver, while the VHF Stack includes 2 receiver assemblies for across-track interferometric measurements. The TxDA consists of a bandpass filter and an amplifier operating in saturation that drives the PAU. A GaN device-based power amplifier operating in a Class-D configuration, a PIN diode T/R switch, and a discrete-element Wilkinson power divider form the transmit path of the PAU. A directional coupler in the T/R switch provides a calibration path to sample the transmitted pulse. The T/R switch also includes a bandpass filter and RF limiter on the receive path to protect the downstream receiver electronics from any potential IESD generated by the out-of-vault elements of the radar and spacecraft.

**Table 9:** Key RFES requirements

Requirement	HF	VHF
Operating Center Frequency	9 MHz	60 MHz
Bandwidth	1 MHz	10 MHz
Peak Transmit Power	7.5 W (min) at each antenna	3.75 W (min) at each antenna
Pulse Droop	<0.005 dB/*μ*s	<0.005 dB/*μ*s
Pulse Ripple Over Bandwidth	<1 dB	<1 dB
Harmonic Rejection	>80 dBc	>80 dBc
Noise Figure	<13.5 dB*	<13.5 dB*
Receive Path Gain	49.5 ± 2.5 dB	59.5 ± 0.25 dB
Amplitude Balance	<0.3 dB	<0.3 dB
Phase Balance	<3 deg	<3 deg
Differential Phase Stability	N/A	<2 deg
Amplitude Stability Outside Calibration Loop	<0.4 dB	<0.4 dB
RF Pulse Width	10 *μ*s to 236 *μ*s	25 *μ*s to 200 *μ*s
Duty Cycle	10% max	10% max

*Requirement driven by Jovian noise

The Rx electronics includes low noise amplification stages and the noise bandwidth filters. The Rx electronics also includes a switch that selects between the receive path from the T/R switch and the calibration path. Suitable digital attenuators are included in the Rx electronics to maximize the dynamic range of the system to allow operation over a wide range of altitudes during a flyby.

JPL designed a Power Supply Unit (PSU) for the HF and VHF RF electronics. The magnetic amplifier (magamp) topology of the PSU was chosen to reduce challenges of qualifying components in a harsh radiation environment. The primary inductor in the magamp is optimized for saturation and serves as the switch operated at 150 kHz. A reset circuit in the PSU resets the magamp on a cycle per cycle basis to transition from a saturated state. Magamps inherently are low noise since there are no EMI spikes associated with field-effect transistor (FET) switching in typical converter topologies. The PSU provides five independent outputs including the primary pulse output to the RF electronics. A key challenge solved on the PSU magnetics was compression of the core by potting and coating resulting in voltage drop out due to a change in the squareness of the ratio of magnetic flux density to magnetic field strength (B/H).

#### Antenna Subsystem (AS)

The REASON AS consists of the HF and VHF antennas and their respective Matching Networks.

##### Antennas

The REASON antennas were built by Heliospace Corporation in Berkeley, California. Heliospace was also responsible for the mechanical design of the antennas, while JPL was responsible for the RF design of the antennas and antenna arrays. The antennas consist of beryllium copper Spiral Tube and Actuator for Controlled Extension and Retraction (STACER) elements that deploy due to their own push force. When deployed, STACERs have a similar bending stiffness to a thin-walled tube, but they stow into a compact volume. The modeled antenna beam patterns are shown in Fig. 25.

As discussed in Sect. 5.1.1, REASON is sensitive to IESD events, therefore materials selection was an important consideration, especially for out-of-vault hardware such as the antennas. Conductive materials were used everywhere possible, but in places that required electrical isolation, polyetheretherketone (PEEK) was used in mechanical joints to isolate parts of the antenna. In order to minimize discharge from PEEK, it was heavily shielded and plated with gold to provide a reliable bleed path. Wire harnessing was shielded in metallic conduit and wrapped with conductive material to minimize discharge amplitudes. Other nonmetallic elements in the actuators and connectors were tested or analyzed as discussed in Sect. 5.1.1. In addition to materials selection, the VHF antenna elements were moved further from the Solar Array, which is a known source of IESD events.

In addition to the EM and FM antenna assembly units, one flight-identical Qualification Model (QM) was built per operating frequency. The QM was subjected to more rigorous testing than the FMs to fully qualify the design for flight environments while reducing risk to the flight hardware. The QM antennas were characterized on the outdoor antenna range at JPL because no indoor ranges are available at this frequency. The FM units were not exposed to this test environment in order to preserve flight cleanliness levels. Instead, a proxy stowed antenna measurement was developed to check antenna aliveness, which is equivalent to making a return loss measurement.

##### HF Antennas

**Fig. 19:**
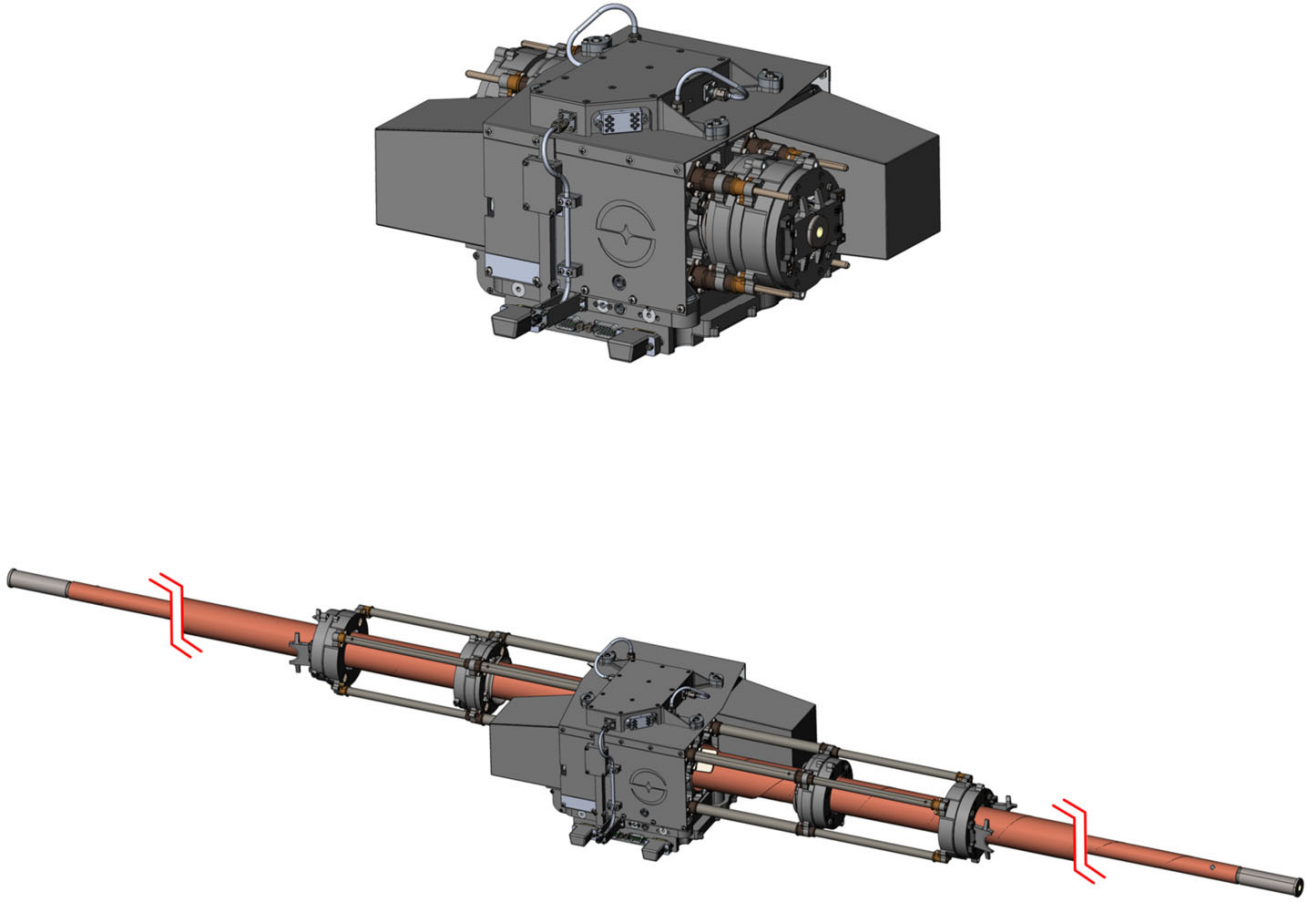
REASON HF stowed (top) and deployed (bottom)

The HF antenna assembly consists of two 8.8-m monopoles that deploy to create a 17.6-m antenna. Deployment is actuated by an Ensign-Bickford Aerospace & Defense (EBAD) Frangibolt on each monopole, and deployment speed is controlled by a flyweight brake attached to a lanyard that runs inside each STACER. It will take less than three minutes to fully deploy. Each monopole has a Deployment Assist Device (DAD) that provides a stiff load path at the base of the STACERs and helps ensure proper formation of the STACER coils. Figure 19 shows the HF antenna assembly stowed and deployed.

##### VHF Antennas

**Fig. 20:**
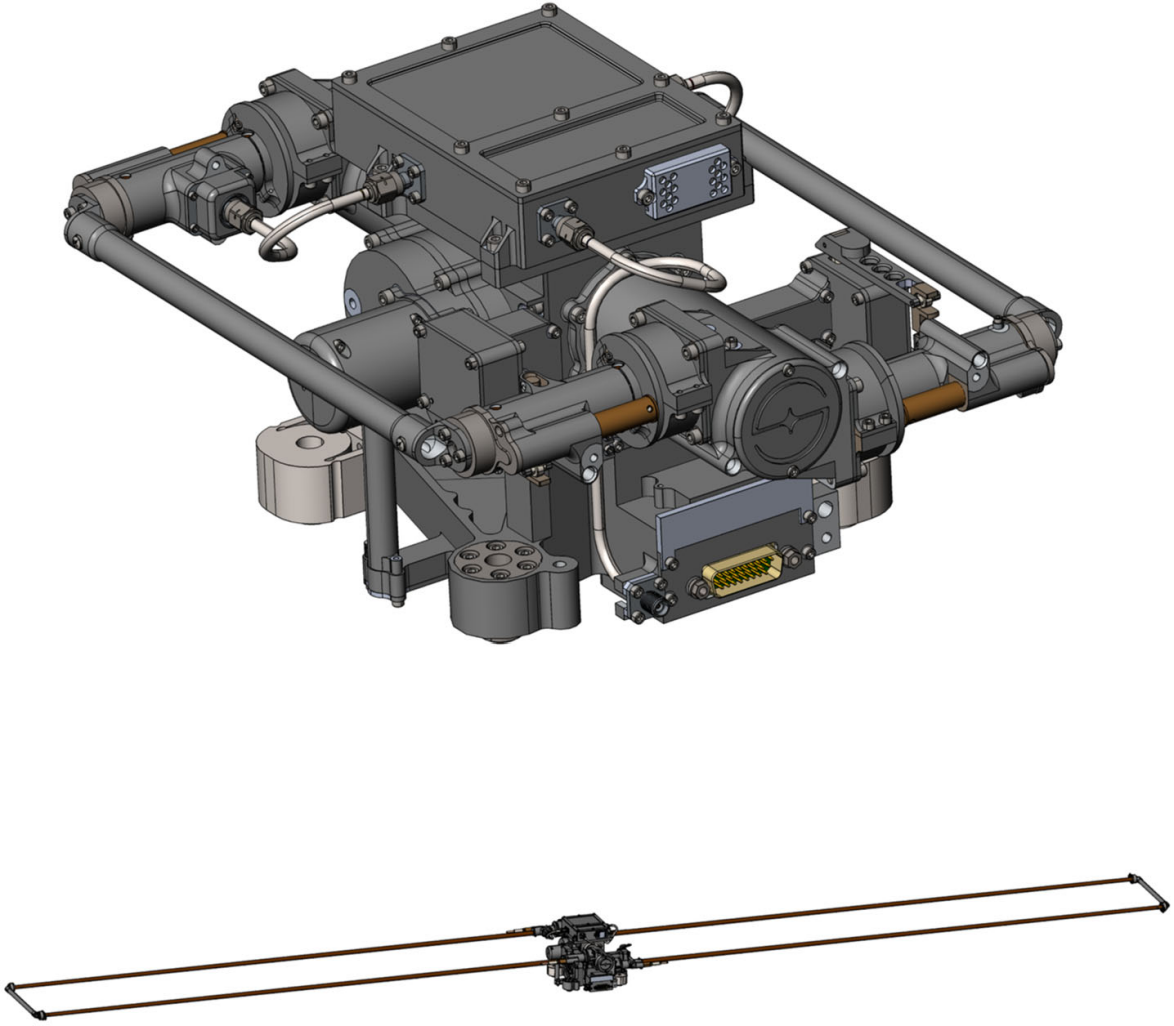
REASON VHF antenna assembly stowed (top) and deployed (bottom)

The VHF antenna assembly is a deployable folded dipole with a tip-to-tip length of 2.76 meters. Each antenna assembly is comprised of a total of four STACERs with two STACERS tied together to form a loop on each side of the antenna. All STACERs have a synchronized deployment due to lanyards that run inside each STACER and around a central axis. Deployment speed is controlled with a flywheel. Deployment is actuated by a pinpuller, and it will take less than one second to fully deploy. Figure 20 shows the VHF antenna assembly stowed and deployed.

##### HF and VHF Matching Networks (MNs)

The antenna MNs are the interface between the RF electronics and the antenna radiating elements. They are responsible for matching the impedance of the electronics to the radiating elements via inductor-capacitor (LC) circuits and a balun. There is one MN per antenna assembly (two for HF and four for VHF). From a material and processes standpoint, the MNs are similar. The challenge of these units is the environment. Given the temperature range (−240 °C to 125 °C) and radiation environment, which includes TID up to 5 Mrads as well as IESD effects, only passive components are used, and the qualification campaign was extensive. Substrates, epoxy, and other materials and processes were carefully selected. Capacitors, inductors, and the baluns were tested to the extreme environments to ensure that performance will hold in the Jovian environment.

#### Coaxial Assemblies

**Fig. 21:**
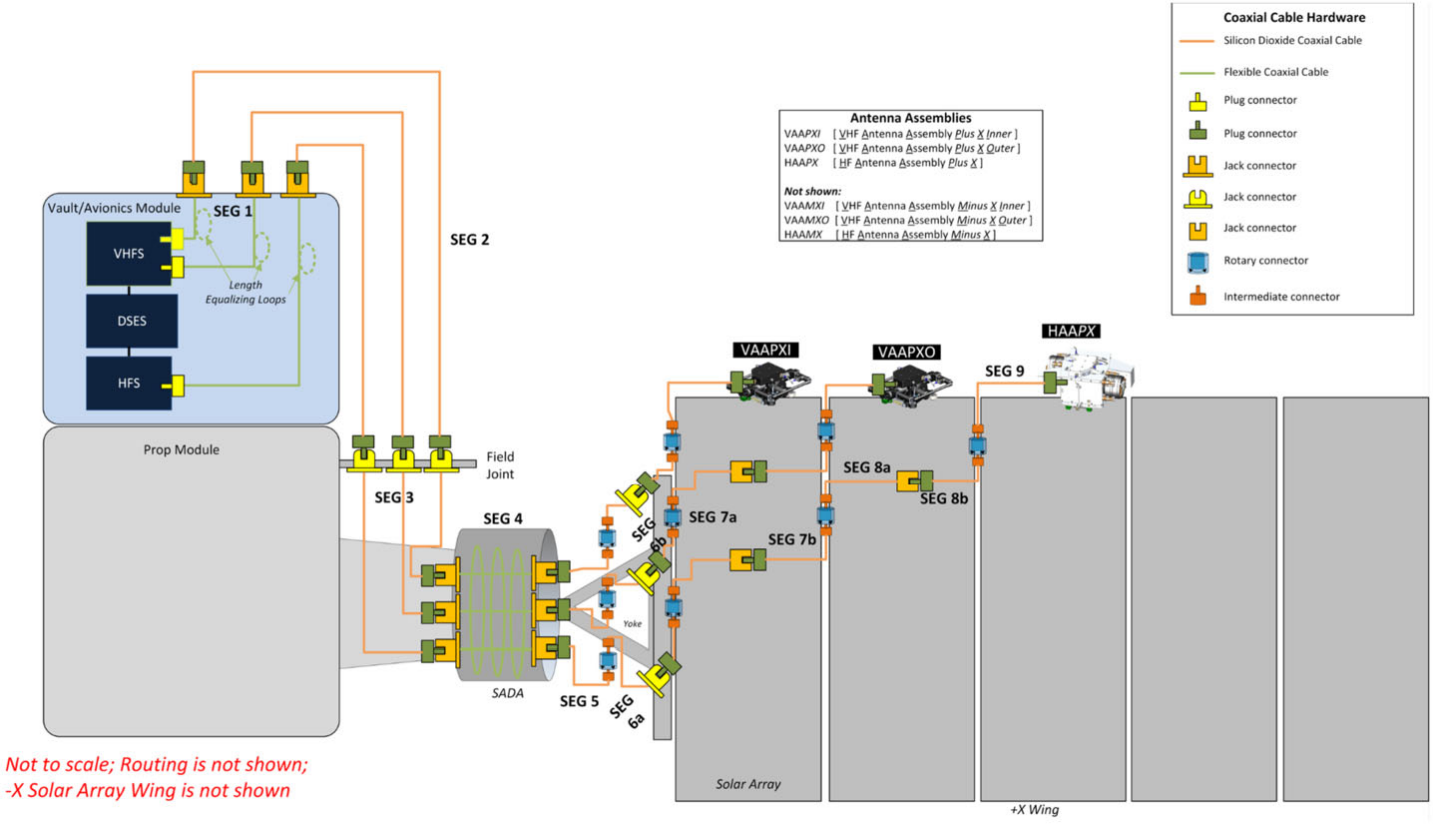
REASON out-of-vault hardware

Coaxial assemblies connect the electronics inside the avionics module (the vault) to the MNs and antennas. Two types of cable assemblies are used. Flexible assemblies are used inside the vault and inside the solar array drive assembly (SADA); this allows for the actuation of the solar array without interruption of the REASON signal path. Semi-rigid SiO_2_ assemblies are used where the cables would be directly exposed to the Jovian environment, as they are more robust to both the temperature and radiation extremes. The entire run of coaxial assemblies between the vault and the antennas were divided into short segments, with each segment consisting of a length of coaxial cable and two connectors. Rotary connectors were also incorporated into the REASON signal path, installed at the solar array hinges, in order to allow for the deployment of the solar arrays without bending or stressing the semi-rigid coaxial cables. A schematic showing the out-of-vault hardware, including antennas, cables, and connectors, is shown in Fig. 21. All cable and connector types were appropriately qualified to their thermal and radiation environments.

### Radar Electronics Integration and Testing

The REASON team integrated and tested the in-vault radar electronics (DSES, HFS, VHFS stacks and inter-stack cable harness), first using the Engineering Model (EM) hardware for test procedure development, and then using the Flight Model (FM) hardware for “run for record” testing. The EM is a close replica of the FM hardware in form, fit, and function, and is slated for use in the Europa Clipper System Testbed (STB) during the mission. The FM was delivered to the Europa Clipper Assembly, Test, and Launch Operations (ATLO) team for spacecraft-level integration. REASON separately tested the EM/QM and FM antenna assemblies, including antenna range testing for the EM/QM. The FM antennas are then delivered to ATLO, where they are integrated and tested onto the spacecraft, along with the vault electronics and coaxial feedline segments.

**Fig. 22:**
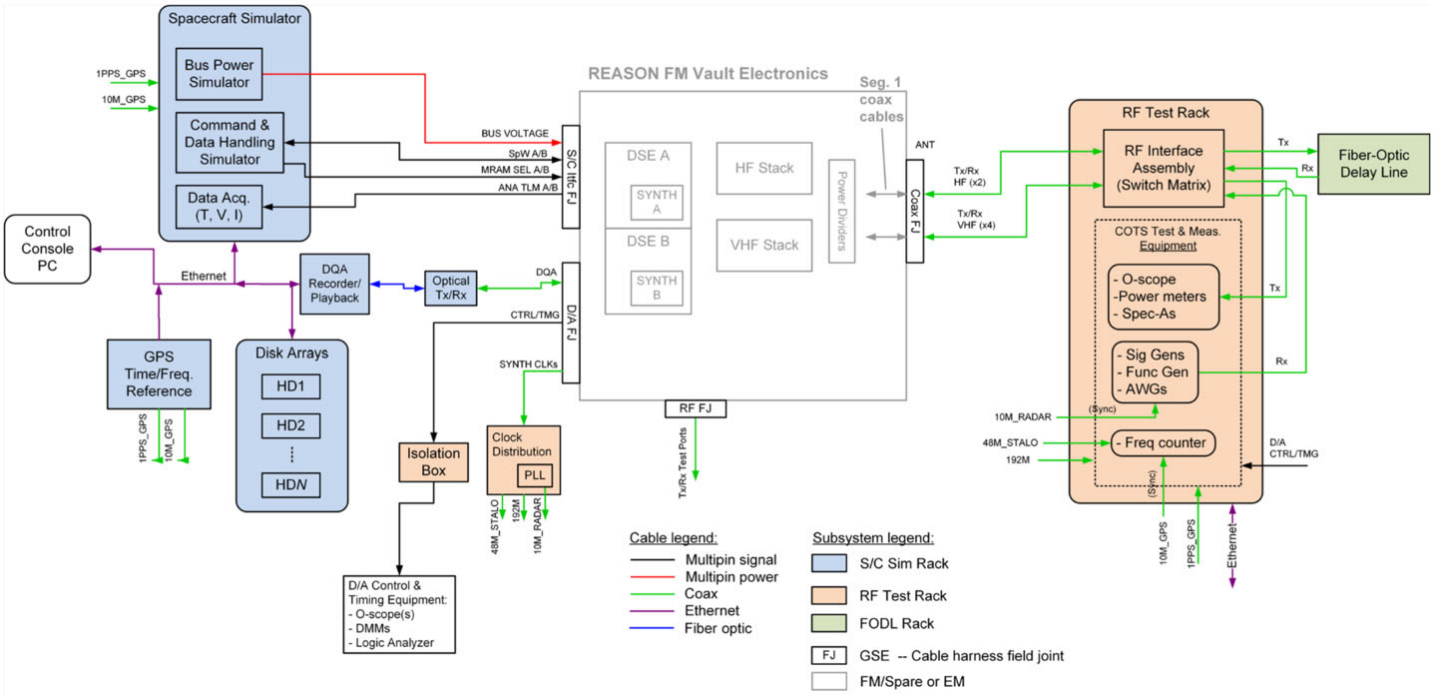
Block diagram of the EGSE used in the in-vault radar hardware I&T campaigns

Before the FM integration phase, the team completed EM vault electronics Integration-and-Test (I&T) with Electronic Ground Support Equipment (EGSE) developed with our partners at NASA GSFC. Figure 22 shows a functional block diagram of the EGSE system, including: a spacecraft simulator that emulates spacecraft electrical interfaces for bus power, SpaceWire command/telemetry, and discrete control of the DSES MRAM configuration bank; an RF test rack that interfaces to the 2 HFS and 4 VHFS antenna ports for acquiring transmit pulse waveform data and injecting simulated echoes into the radar receiver; and a Fiber Optic Delay Line (FODL) to test the radar point target response using a fixed round-trip delay for the echo pulses.

The RF Test Rack contains a programmable switch matrix for routing the antenna port Tx/Rx signals to several types of commercial, off-the-shelf (COTS) test-and-measurement equipment for monitoring transmit pulses and injecting on receive. A control console with EGSE software communicates with the switch matrix and COTS equipment to accommodate automated testing of all six antenna ports via a software script. The EGSE system includes radar target simulator capabilities. The target simulator is composed of several components – RF signal generators, a waveform function generator, the FODL, and Arbitrary Waveform Generators (AWGs) – which together generate several classes of echo signals (continuous waveform, pulsed waveforms, linear frequency modulated chirp point targets, noise, distributed targets) at both HF- and VHF-bands for comprehensively testing the radar.

The radar electronics are designed with direct-access test port provisions to allow us to synchronize the EGSE to the radar in time and to lock them in phase. The EGSE is externally triggered via direct access control-and-timing signal markers coming from the radar. For example, these markers can indicate the start of an OP or the start of a particular dwell within the OP, among several other possibilities. An RF test port from the DSE/Synthesizer provides a copy of the 48-MHz STALO to an EGSE phase-lock multiplier, from which a synchronous 10-MHz reference clock is derived and distributed to the EGSE COTS equipment. These provisions allow phase-coherent testing of the radar and synchronization of the EGSE to each dwell interval within the OP.

The team ran several radar functional and performance tests on the integrated vault electronics/EGSE system to verify instrument-level requirements. EM tests were performed in an ambient environment with the Ground Support Equipment (GSE) staged in the same electrical configuration (including thermal vacuum-compatible test cables) planned later for FM environmental testing. These EM trial runs were valuable in order to identify and resolve any remaining issues in the test setup and helped to facilitate a successful FM test execution.

FM vault electronics testing included ambient and environmental test phases. During the environmental phase, the team successfully completed a month-long Thermal Vacuum (TVAC) test campaign over the flight acceptance temperature range (−25 °C to +55 °C over three cycles, plus +70 C bakeout). TVAC was followed by an 11-day Electromagnetic Interference and Compatibility (EMI/EMC) test in an anechoic chamber; all of these tests passed the Europa Clipper environmental requirements for radiated and conducted emissions/susceptibility.

As the DSES FPGA firmware features were being developed, Instrument Integration-and-Test (II&T) had opportunities to test the firmware configuration file upload capability via SpaceWire command to MRAM. These tests verified a key requirement that the firmware be re-programmable during the mission. II&T likewise gained experience running a suite of functional and performance regression tests when new versions of the firmware were released by the firmware team.

## Instrument Operations

### Description of Instrument Science Operations Center

The REASON Instrument Science Operations Center (ISOC) interfaces with the Europa Clipper Mission Operations System (MOS). The ISOC consists of three elements: the Experiment Planning System (EPS), the Instrument Operations System (IOS), and the instrument Science Data System (ISDS). The EPS is located primarily at the Johns Hopkins University Applied Physics Laboratory (APL), the IOS is located primarily at JPL, and the iSDS is located primarily at the University of Texas Institute for Geophysics (UTIG). During the Europa tour, the ISOC elements, each assisted by a REASON liaison to the Europa Clipper Project’s Tactical Science Group (TSG), will engage in regular meetings for consensus on flyby planning, science production, and instrument operations objectives and issues. During Europa Campaigns 1 and 2 (EC1, EC2), when flybys of Europa occur every 2–3 weeks, ISOC interactions may be frequent and intense.

### Experiment Planning System (EPS)

The EPS is primarily tasked with planning REASON Europa flyby operations during the Europa Campaigns to meet science requirements and iST objectives. The EPS will establish observational priorities in consultation with the rest of the ISOC. The EPS will track science requirement compliance and progress towards meeting these objectives and REASON Planning Guidelines defined for the Europa Clipper mission (Table 1).

The EPS is the primary element of the ISOC to interact with the Europa Clipper Science Planning Operations Coordination (SPOC) team during the long-range mission planning and science and instrument planning phases of the uplink planning process, all under the Europa Clipper Science and Instrument Operations Subsystem (SIOS). The EPS will need to review and provide input for potential updates of the Reference Activity Plan (RAP) for Europa Clipper mission planning every week.

The EPS will define, develop, implement, and operate a variety of planning and visualization tools. These will be based on the Cadmus tool currently in use by the EPS for Europa tour and trajectory evaluation. The EPS will feed flyby definition information to the IOS, which will develop the corresponding radar command files.

### Instrument Operations System (IOS)

The IOS is primarily tasked with instrument operations. On the uplink planning side, the IOS builds and tests instrument command and control files (i.e., OPs), based on input received from the EPS. The IOS interfaces with the SPOC to deliver uplink products and review command sequences as part of the science and instrument planning and sequence planning and generation processes.

On the downlink side, the IOS evaluates instrument health, status, successful data collection, and hardware performance. It feeds this information as needed back to the EPS, iSDS, and iST, as well as the Europa Clipper MOS. The IOS generates a raw data product file from the REASON downlink data and produces a Partially Processed Data Product (PPDP) file containing REASON and Spacecraft data and ancillary Ephemeris and related information. Both files are passed to the iSDS for further processing and eventual delivery to the Planetary Data System (PDS). The earliest downlinked data, known as “Feed-Forward data”, are used to initially evaluate instrument health and to assist in fine-tuning flyby operations over the next few Europa orbits.

The IOS will develop, test, and maintain several software tools and interface mechanisms for producing and delivering the REASON command products and for monitoring and evaluating REASON health and performance over the life of the mission. The IOS will participate as a contributing member in science planning and prioritization, as well as science data evaluation and analysis.

### Instrument Science Data System (iSDS)

The iSDS is primarily tasked with generating the REASON science data products, following the guidance of the Europa Clipper Science Data Management Plan (Sect. 8).

This iSDS will monitor and evaluate science data product quality, and will develop, maintain, and update as needed the algorithms and science data processing software needed to generate the data products. The iSDS interfaces with Europa Clipper primarily through the mission SDS (m-SDS) and participates both in the MOS as well as the Europa Clipper Science System. The iSDS will distribute archival data to the PDS via the Geoscience Node at Washington University in St. Louis.

### Instrument Science Team (iST)

Each element of the ISOC has a designated liaison to the broader REASON iST, which includes deep expertise in both radar science and planetary science of icy worlds. These liaisons manage the iST contributions by defining, refining, and monitoring progress toward meeting REASON science measurement requirements (Sect. 4) as well as defining data products and algorithm development priorities for the iSDS. During the Europa tour, the iST will evaluate and validate science data products and provide input to the Europa Clipper Project Science Team. The iST, assisted by their liaisons to the ISOC elements, is responsible for generating and testing science hypotheses to feed into the long-range mission planning which will be used to prioritize observations by the EPS.

### Instrument Concept of Operations

**Fig. 23:**
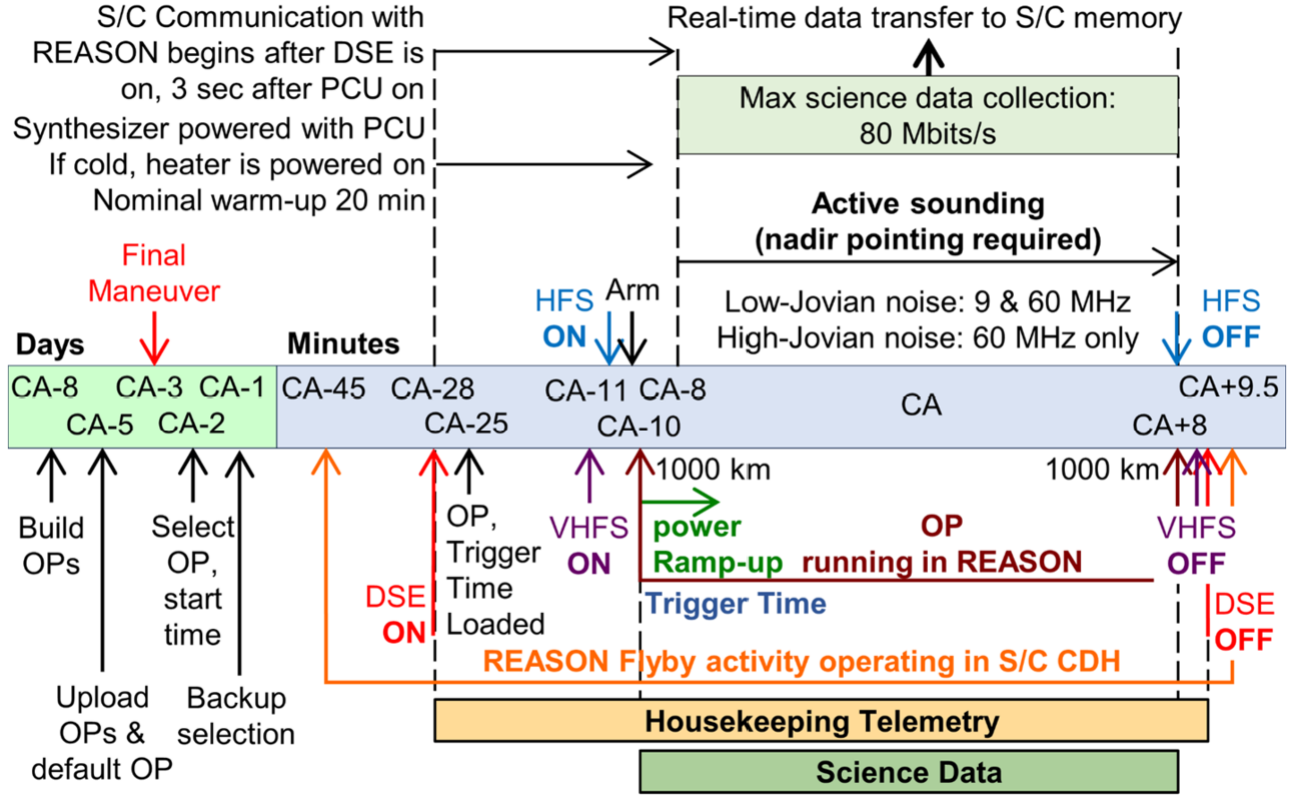
The sequence of events and timing for a typical REASON flyby

Figure 23 illustrates the concept of operations for REASON. The control of the REASON radar is primarily through an Observation Plan (OP) file. A set of OP files is uploaded to the spacecraft early in the orbit. After the final trajectory maneuver, the OP most consistent with the updated trajectory is uploaded to REASON. It is activated typically 10 minutes prior to closest approach, about 20 minutes after REASON power on, and controls REASON behavior for the next 18–20 minutes through closest approach.

During a typical flyby, both HF and VHF radar data will be collected, HF for full depth sounding (surface to ∼30 km sub-surface), and VHF +X and –X channels for shallow sounding (surface to ∼3 km depth), which are all multi-bit, high dynamic range data sets, and full-depth VHF (FDVHF, up to 30 km depth), which is a single-bit, low dynamic range data set. FDVHF is used for full depth sounding when the Jovian noise environment prohibits full-depth HF operations, plus it provides higher spatial resolution than the HF data.

As the spacecraft altitude changes, many radar parameters must be adjusted due to the constantly changing distance from the target and round-trip travel time for the radar pulses (Table 10). The OP captures all these variations over the length of a flyby.

**Table 10:** Radar Parameters Adjustable Over a Flyby

Radar Parameter	Description
Pulse Repetition Frequency (PRF)	Used to collect the desired along-track density of measurements and to optimize usage of the round-trip echo travel times (more than one pulse may be in the air at one time). In the OP, this is also referred to as the Cycle Repetition Frequency (CRF), as the dual-frequency PRF may be more complex than in a single frequency radar.
Chirp (transmit pulse)	Used to optimize SNR given round-trip echo time and the PRF
Receive window duration and position	Used to collect surface and subsurface echoes
Receiver gain	Used to correct for echo power variations with altitude and environment
Number of on-board pre-summed pulses	Used to limit data volume
Number of bits in digitized echo	Used to limit data volume while maintaining dynamic range

REASON does not provide any internal data storage. All data, both housekeeping telemetry and science data, are immediately sent to the mass memory (Bulk Data Storage, BDS) of the spacecraft for storage and eventual downlinking to Earth. Data during a flyby are accumulated in about 16 minutes, and are to be downlinked to Earth within one, and hopefully no more than two, orbits of Jupiter (approximately two weeks per orbit). If the entire flyby dataset cannot be downlinked in a timely fashion, a subset of data sufficient to verify key performance during the flyby and sufficient to plan subsequent flybys will be downlinked within the required time frame. The ordering of data for science prioritization can be selected before the flyby and integrated into the OP, while the actual downlink prioritization can be modified after the fact. Instrument health and status telemetry and other “feed-forward” data are nominally given highest priority. The data packet types collected are shown in Table 11.

**Table 11:** Packet Types Generated by REASON sent to BDS

Packet Type	Packet description
Health & Status (H&S) Telemetry	Telemetry to monitor health/status/safety, sent to both BDS and Clipper Avionics for fault monitoring
Engineering Telemetry	Currents and voltage telemetry sampled at 8 Hz
Processing parameters	Dwell parameter sets and time sync information
HF loopback*	HF reference waveform
HF receive*	HF echo window
VHF loopback (+X) starboard*	VHF reference waveform
VHF loopback (-X) port*	VHF reference waveform
VHF +X 10 MHz receive*	VHF echo window, starboard side, 10–MHz sampling
VHF -X 10 MHz receive*	VHF echo window, portside, 10–MHz sampling
VHF 1-bit full depth sounding receive	FDVHF combined channel, 1-bit, 10–MHz sampling
Raw data (HF, VHF ±X)	Raw data, for diagnostic purposes
BFPQ exponents (H, VHF ±X)	Exponents only from above packets for quick power assessments

* These data sets use BFPQ, a data compression method.

## Characterization of REASON Antennas

**Fig. 24:**
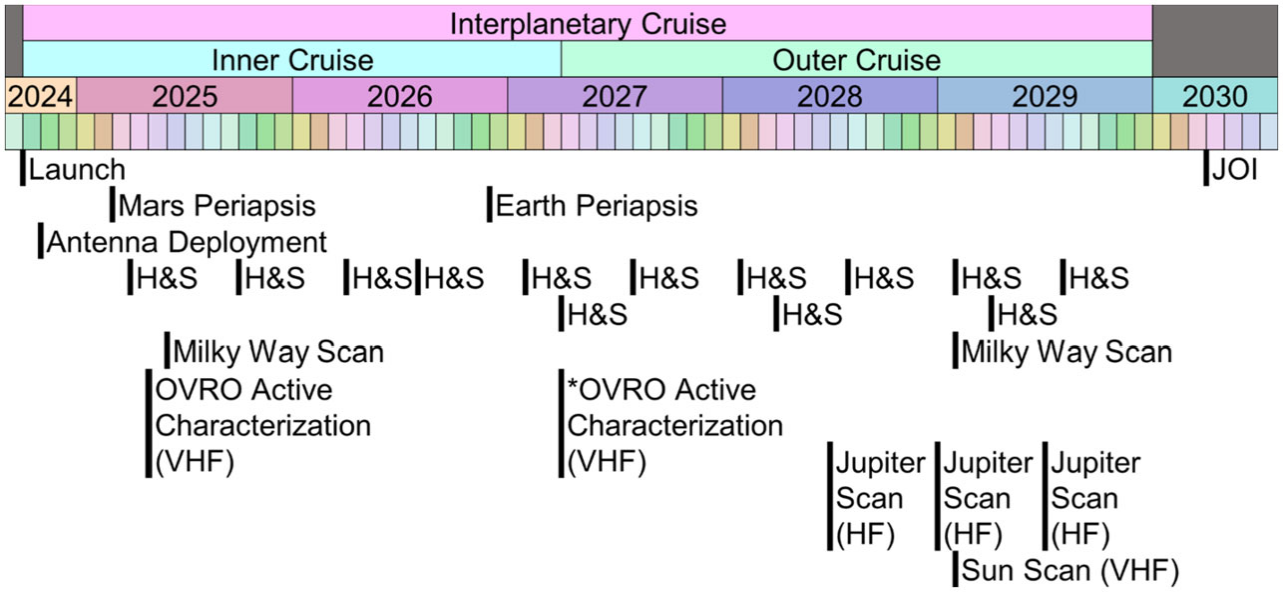
Visualization of planned cruise activities for REASON ($+/-$ 1 month), the asterisk denotes a backup activity awaiting approval

Understanding the performance of REASON’s antennas given their thermal environment and their interactions with the spacecraft is a major challenge. Prior to launch, there will be no testing of the individual antenna elements at flight-like, cryogenic temperatures; the primary validation and verification of the antennas will be done by modeling. Additionally, because of the size of both the antenna hardware and the Europa Clipper solar arrays, the fully integrated spacecraft/solar array/antenna structure cannot be tested end-to-end on the ground and must be characterized *in situ*. Intrinsic characteristics of the fully assembled and deployed antenna, in particular the directivity beam patterns and interactions with the spacecraft, the other instruments and the external electromagnetic environment will be measurable after launch. For this reason, calibration and characterization activities for the antennas are planned from the post-launch stage through the tour stage of the Europa mission (Fig. 24). As many of these activities as possible are to be scheduled before the first Europa flyby. The activities consider improved calibration geometries earlier in the mission, flyby-like antenna element temperatures later in cruise, spacecraft power/energy limitations after Jupiter Orbit Insertion (JOI), and the need to try a variety of characterization options as the effectiveness of any one technique is not assured.

### Cruise Characterization

**Fig. 25:**
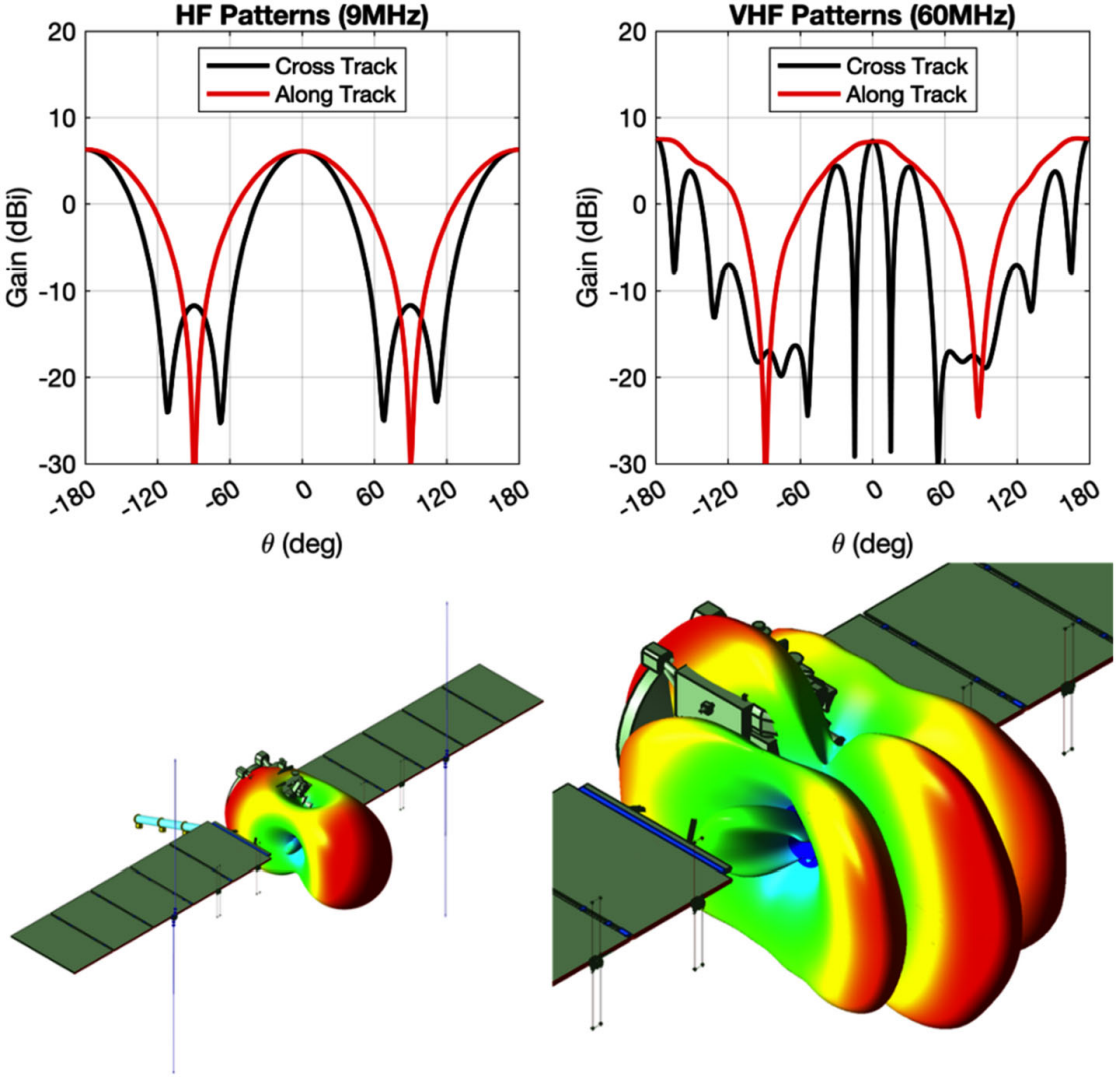
Modeled HF (left) and VHF (right) antenna beam patterns, referenced to their phase centers at the center of the spacecraft bus

Cruise-phase activities can be classified into three groups: instrument checks, passive beam characterization, and active beam characterization. Models of the beam pattern are shown in Fig. 25. Instrument checks involve powering on REASON electronics, loading and running health check OPs on the internal electronics, collecting the results of the OP, transmitting them back to Earth, and powering down. Passive beam characterization requires powering on REASON electronics and using the antennas in receive mode while the spacecraft slews in a predetermined manner to observe external radio sources to determine the shape of the antenna beam patterns. Active beam characterization is similar to passive beam characterization, but the instrument also transmits, either to a remote receiver or toward a target (from which return data is collected).

#### Instrument Checks

The REASON instrument has a maintenance requirement for a power-on every six months during cruise, beginning as early as possible. These power-on instances will allow instrument health checks using both internal and external sources. The instrument will operate the HF- and VHF-band electronics with the antenna staring at regions of the sky with known galactic background emission levels that are also free from point sources at 9 and 60 MHz (“cold sky”). These tests consist of digitizing the HF-and VHF-band channels in the receive-only mode, stepping through the receive attenuator settings, and evaluating the change in value that digitized signals exhibit during ground processing.

#### Owens Valley Radio Observatory (OVRO) VHF Active Characterization

The REASON VHF directivity pattern is expected to be very wide along-track but contains regions of sensitivity (beam pattern side lobes) and regions of insensitivity (beam pattern nulls) cross-track. Because the array beam pattern cannot be fully characterized prior to launch, obtaining an accurate understanding of the beam pattern post-launch and post-deployment is a priority for REASON. One way to characterize the VHF antenna pattern is through direct transmission to a radio facility on Earth. The REASON system engineering team has been working with California Institute of Technology (Caltech) to design a characterization activity using the 352-element Owens Valley Radar Observatory Long Wavelength Array (OVRO-LWA) in Bishop, California as a receiver for REASON transmissions post-launch. OVRO is operated by Caltech. This activity can also be used to detect a deployment failure.

#### Jupiter HF Passive Characterization

The REASON HF directivity pattern is expected to be largely symmetric. Validating the modeled directivity pattern for the HF antenna will require a passive raster or spiral scan of a strong HF source. Jupiter is a natural source of strong decameter radiation up to 44 MHz. Once the instrument is close enough to distinguish this signal from the galactic background noise, a raster or spiral scan of the planet’s disk can be used to probe the HF beam pattern. When the spacecraft is within 1–2 AU of Jupiter there will be an adequate SNR for REASON to detect this signal, while the planet’s disk will still appear small within the antennas’ fields of view. A difficulty in using Jupiter as an HF source for beam pattern characterization is the variability in Jupiter’s radio signal strength (Carrer et al. 2018, 2021). There is some regularity to the pattern of Jovian radio emissions, but they are very unstable, often varying in magnitude over the course of minutes. Unpredictable bursts occur across many frequencies and may last several hours. Due to this Jovian noise variability, the HF beam characterization activity is expected to obtain coarse HF beam pattern knowledge, mostly as a sanity check. It is not expected to provide highly accurate beam pattern characterization. However, coarse knowledge is enough to meet the following goals 1) to know that the instrument is properly receiving the signal and 2) to locate antenna pattern nulls.

#### Milky Way HF and VHF Passive Characterization

The Milky Way galactic plane is a constant source of incoherent radio emissions over a wide range of frequencies. This radio noise is known as the “galactic background”. The average magnitude of emissions from 1–50 MHz is well-known and was used to calibrate the Cassini radar over its range of frequencies (Zarka et al. 2004). The galactic background shows a bright band in the plane of the Milky Way, with emissions highest near the galactic center. Several discrete sources of radio transmissions add to the Milky Way emission pattern. Given a galactic background pattern with sufficient accuracy, or knowledge of a few dominant point sources, it may be possible to deconvolve the HF or VHF antenna patterns from a collection of noise measurements over a set of spacecraft orientations. This activity requires maps of galactic background emissions at or near 9 MHz and 60 MHz with sufficient resolution for comparison to REASON measurements. Many partial maps of Milky Way radio emissions have been created at VHF frequencies (de Oliveira-Costa et al. 2008; Dowell et al. 2017). These maps vary in resolution and coverage. However, the Milky Way radio pattern is not well characterized in HF. A model describing galactic emissions from approximately 10–100 MHz, along with Fortran code to implement it, is presented in de Oliveira-Costa (2008). The REASON team has used this model to generate galactic background temperature maps at 9 MHz and 60 MHz.

#### The Sun VHF Passive Characterization

The Sun is a natural source of weak radio emissions, with the intensity of normal solar emissions at 60 MHz falling below that of galactic radio emissions at Earth, and the intensity at 9 MHz even lower. Higher intensity, but unpredictable, solar bursts and storms may provide strong emissions that could be used to characterize the REASON antennas. For these solar radio outbursts to be useful, REASON measurements of bursts and storms would need to be cross-checked with Earth-based radio telescopes or dedicated solar missions (e.g., Solar Terrestrial Relations Observatory (STEREO), Wind, Parker Solar Probe). If this could be successfully arranged, it could provide a valuable comparison for calibration of the VHF beam pattern. This activity has the best chance of success if performed near the peak of solar activity.

### Calibration and Characterization Activities After Jupiter Orbit Insertion (JOI)

#### Ganymede Flyby

A flyby of Ganymede will provide important information about the active performance of the radar subsystems prior to the first Europa flyby. The data collected at this flyby will be used to validate the REASON command parameter design and to fine-tune key radar parameters, as well as to characterize HF/VHF band transmitted RF power levels, nadir beam realized gains, and beam patterns at Europan flyby temperatures.

During a Ganymede flyby, REASON turns on at a high altitude (10,000 km). This contrasts with a Europan flyby during which REASON turns on at approximately 1000 km in altitude (Fig. 23). Two approaches will be taken to REASON’s high-altitude operation: slewing and non-slewing. The slewing approach requires slewing the spacecraft cross-track in order to provide active beam characterization data for the antenna beams. This approach will be taken if the beam patterns remain poorly characterized despite the beam characterization activities described above. The high altitude will mitigate the effects of surface inhomogeneity and altitude changes on beam pattern characterization. A separate, non-slewing, high-altitude flyby will be used to collect average backscatter values to increase the science return of the mission, and possibly for comparison with data from the Radar for Icy Moon Exploration (RIME) on ESA’s JUICE mission (Sect. 7.2.3). For one of these flybys, below 1000 km the spacecraft will transition to a steady state with nadir pointed toward the satellite surface for a low-altitude (below 400 km) pass. During this low-altitude pass, data collected will be used to validate the REASON command parameter design and to fine-tune key radar parameters, as well as to characterize HF/VHF band transmitted RF power levels and nadir beam realized gains.

#### Internal Electrostatic Discharge (IESD) Characterization

To allow REASON to learn about IESD levels and their impact on REASON’s data volume, REASON will conduct one or more in-flight IESD tests when the spacecraft is in a radiation environment similar to that found at Europa. This test requires all instruments which violate REASON’s EMI/EMC requirements to be off and therefore cannot take place during CA of a Europa flyby. Instead, these tests will take place when the spacecraft comes to or within the orbit of Europa, with no Europa nearby. Candidate events include during a Ganymede or Callisto flyby, or on the retreating end of a Europan flyby. During these tests, REASON will collect HF and VHF receive-only data to quantify the level of electrostatic discharge (also known as “snap, crackle, pop”) from the spacecraft including solar arrays generated by buildup from Jupiter’s intense radiation environment.

#### Concurrence with RIME

Galilean moon flybys will allow REASON to take advantage of data collected by the European Space Agency’s JUICE spacecraft, which will be touring the Jovian system concurrently with REASON. RIME’s CBE arrival date is 2032. The JUICE RIME instrument operates at a frequency of 9 MHz radar and is very close in design to REASON’s HF system. JUICE will conduct flybys of Callisto, Europa, and Ganymede, and will orbit Ganymede. This provides unprecedented calibration and science opportunities for the REASON team to compare surface backscatter values for these planetary surfaces with observations acquired by RIME.

## Data Processing and Archiving

**Fig. 26:**
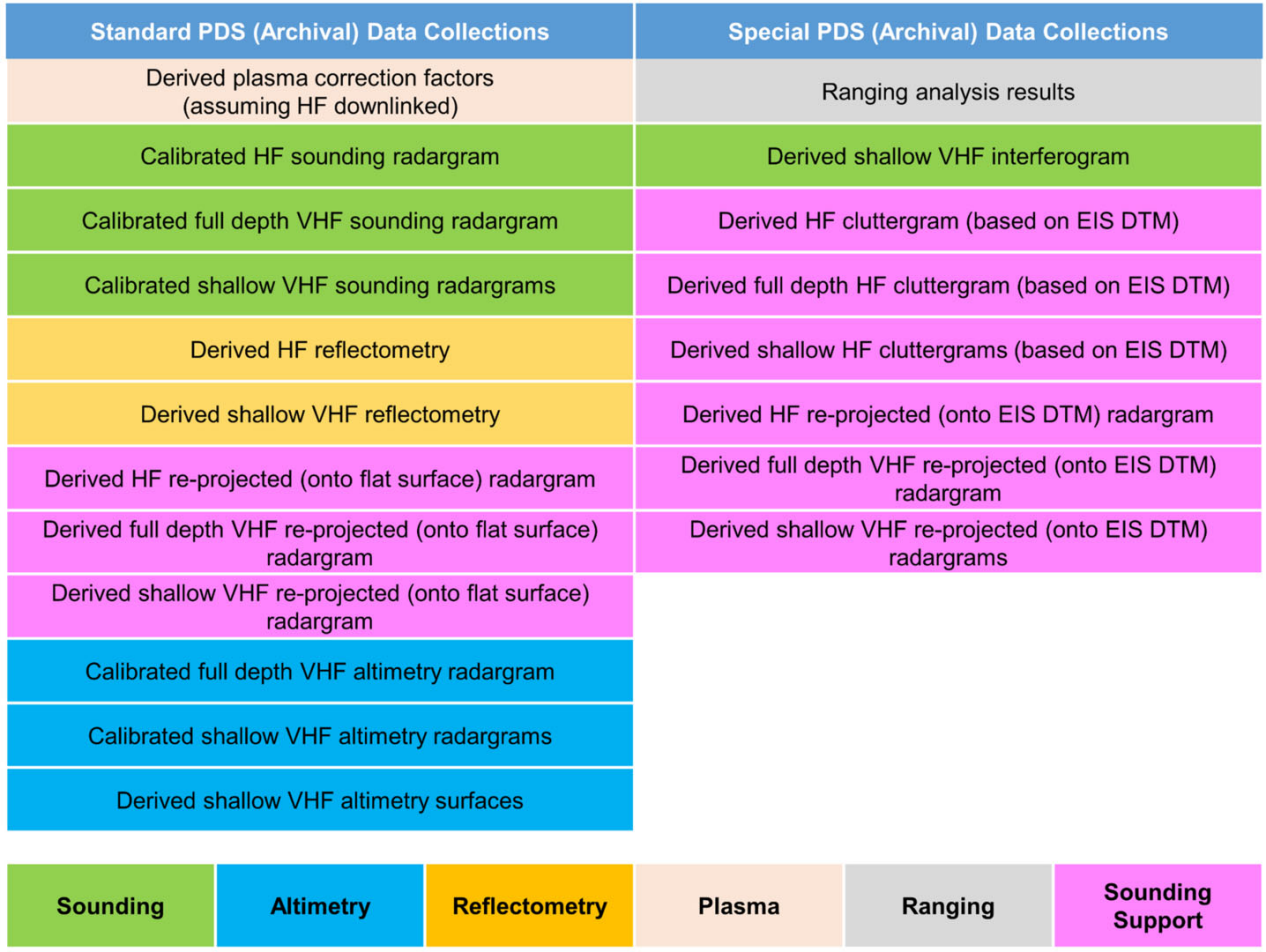
REASON PDS data collections

REASON generates multiple data products which are distinguished by their PDS4 level of processing (e.g., Raw, Partially Processed, Calibrated, and Derived) or to reflect whether the processing is routine (e.g., Standard) or ad hoc (e.g., Special) (Hughes et al. 2014). Standard and Special products are shown in Fig. 26. Once REASON data have been downlinked from Europa Clipper, versions of the Raw Data Products (RAWs) and Partially Processed Data Products (PPDPs) are generated by the IOS where they are then securely transferred to the iSDS and converted into a PDS4-compatible format (Hughes et al. 2014). The PPDPs also serve as the input from which the iSDS produces Calibrated, Derived, and Special Data Products. In general, Calibrated Data Products are two-dimensional radargrams, whereas Derived Data Products are georeferenced text profiles of properties extracted from radargrams and can be either one dimensional profile data or, in the case of clutter discrimination-related data products, two-dimensional data arrays. Raw, Partially Processed, and Calibrated REASON Data Products will be delivered to PDS for archiving within six months of receiving required science and ancillary data products from the Europa Clipper project. Derived and Special Data Products are expected to take longer to generate and may not be archived until after the end of the primary mission. Note that as of publication Raw and PPDP formats are undergoing review by the PDS. Users are referred to the final published PDS Software Interface Specification documents for data formats.

### Raw Data Products (RAWs)

REASON raw data products provide the complete content of the radar science and telemetry data downloaded from the Europa Clipper spacecraft for a specified flyby of Europa or for other data collections. Raw data products are extracted from original downlink data packets and minimally processed. Any compression, reformatting, packetization or translation applied for the purposes of transmission or storage will be reversed to ensure archived data is in a PDS-approved format. Information relevant to instrument commanding, command responses, and firmware state and health that are not relevant to science performance and science data processing are not included as part of the raw products.

### Partially Processed Data Products (PPDPs)

REASON PPDPs contain all the science data, ancillary data, and telemetry needed to produce the REASON science product for a specified flyby of Europa. Internally, PPDPs are distributed as Hierarchical Data Format v 5 files (HDF5). For archiving at the PDS, PPDPs are organized in folders by encounter and then files for each channel with science and engineering data files as well as ancillary data files for relevant spacecraft telemetry, instrument telemetry, and intermediate data (e.g., trajectory information and geometry information). Science data are stored in binary three-dimensional arrays and complementary channel-specific data are stored as text tab-delimited tables format. The Engineering Data File contains the parameters that define the state of the radar, such as mode, cycle duration, and BFPQ bit depths. It also includes data quality metrics such as noise levels and the correlation between subsequent fast-time records. The Intermediate Level Data File contains elements that specify timing, spacecraft state vector, and moon and planet ephemerides derived from SPICE.^12^ The Instrument Telemetry Data File contains the elements that specify the status such as temperatures of REASON subsystems. The Spacecraft Telemetry Data File contains the temperatures of several REASON components located outside the vault, mostly the antennas and signal paths leading to the antennas.

### Calibrated Data Products

For REASON, Calibrated Data Products are two-dimensional radargrams. There are three types of REASON Calibrated Data Products: (i) unfocused *altimetry* radargrams, (ii) single-look complex-valued SAR focused *sounding* radargrams, and (iii) multi-look SAR focused *sounding* radargrams. Unfocused *altimetry* and multi-look SAR focused *sounding* data products are each comprised of two distinct deliverables: (i) A binary array of 32-bit integers corresponding to the radargram magnitudes (dB × scale factor), where fast-time records are padded such that they are all the same length and the surface echoes are properly aligned, and (ii) an ascii table of metadata containing timing and geolocation data associated with each fast-time record in the corresponding byte array. In addition to these two deliverables, the single-look complex-valued SAR focused data product contain a third deliverable: a binary array of 32-bit integers corresponding to the radargram phase (radians × scale factor) where fast-time records are padded such that they are all the same length, and the surface echoes are properly aligned. There are three processing modules used to generate these Calibrated Data Products: (i) range compression and ionospheric correction module, (ii) *sounding* module, and (iii) *altimetry* module.

#### Range Compression and Ionospheric Correction Module

Range compression and ionospheric corrections represent two fundamental processing steps critical for interpretation of REASON data. Echoes recorded in unprocessed fast time records are reflected versions of the chirp from the surface and subsurface. Energy from these echoes is often overlapping making them challenging to differentiate and interpret. Range compression is used to compress the energy in the reflected chirps; thereby improving the SNR of surface and subsurface reflections as well as enhancing fast time resolution. Any electromagnetic wave propagating through a charged plasma may experience attenuating and/or dispersive (i.e., radar waves of different frequencies travel at different velocities) effects due to its interaction with the various constituent particles. Ionospheric correction is used to estimate and correct for phase delays caused by ionospheric effects across the HF and VHF signal bandwidths (Grima et al. 2015; Scanlan et al. 2019). The REASON range compression and ionospheric correction processor operates on a dwell-by-dwell basis producing a set of range compressed and ionosphere corrected fast time records as well as an estimate of the average TEC of the ionosphere between Europa Clipper and Europa during the dwell.

#### Sounding Module

SAR focusing is a key step in the processing of space-borne radar sounding data. Mass and power constraints limit the design of REASON HF and VHF antennas to folded dipoles, which produce wide beam patterns in both the along-track (azimuthal) and cross-track directions. Wider beam patterns have larger footprints (i.e., the portion of Europa’s surface illuminated by a single REASON chirp), which limits the ability to resolve closely spaced surface and subsurface targets of interest.

SAR processing attempts to reduce the along-track extent of REASON footprints by creating a synthetic antenna with an aperture equal to the Fresnel zone radius. The predictable response of a nadir point target in the middle of the synthetic aperture is then used to coherently re-combine (focus) the echoes recorded by REASON across the synthetic aperture to their corresponding position on the mid-aperture fast time record. This focusing decreases the size of the radar footprint in the along-track direction but note that the cross-track extent of the footprint remains unchanged. Repeating the process along the groundtrack yields a focused radargram with enhanced along-track resolution and improved SNR.

While multiple SAR focusing algorithms exist for use with a variety of different data sets (e.g., Cumming and Wong 2005; Peters et al. 2007a; Kobayashi et al. 2011), the REASON sounding processor is based on the delay Doppler algorithm used in the processing of Martian SHARAD sounding data sets (Campbell et al. 2013a). The delay Doppler technique was chosen as the base algorithm for REASON due to its simplicity (and therefore more readily modifiable considering REASON idiosyncrasies) and multiple decades of heritage with SHARAD.

REASON is distinct compared to previous space-borne radar sounders (SHARAD, MARSIS, LRS) due to the combination of its Doppler sampling requirements and the design of the Europa Clipper mission. A detailed description of how REASON’s Doppler sampling requirements and the Europa Clipper mission design give rise to these unique idiosyncrasies is provided in Scanlan et al. (2021).

#### Altimetry Module

The *altimetry* processor has the objective to create height profiles along the groundtrack of the spacecraft between 1000 km altitude and closest approach. The profiles might be biased by residual ionospheric effects and are only designed for relative accuracy (not as an absolute geodetic reference). The input data for the processor are pulse compressed and ionospherically corrected radargrams.

In order to retrieve the altimetric return, classical altimetry processing techniques rely on leading edge detection, tracking the radar echo centroid, or maximum likelihood estimations as applied for instance to the Cassini radar. The REASON *altimetry* processor uses a ß-5 retracking algorithm which will produce a range estimate of the leading-edge position of the waveform (LEP). The ß-5 tracker has been developed to measure ranges from the Seasat radar altimeter over continental ice sheets and is also known as the NASA GSFC V4 retracker. For a detailed description of the method and its application to SHARAD data see Steinbrügge et al. (2021).

### Derived Data Products

Derived Data Products are results that have been generated using one or more Calibrated Data Products. There are six REASON Derived Data Products: (i) the TEC (plasma) profile, (ii) *altimetry* profile, (iii) *reflectometry* profile, (iv) interferogram and correlation map images, (v) reprojected radargram image, and (vi) cluttergram image. There are five processing modules used to generate these derived data products: (i) *altimetry* module, (ii) *reflectometry* module, (iii) *interferometry* clutter discrimination module, (iv) reprojected radargram clutter discrimination module, and (v) cluttergram clutter discrimination module.

### Special Data Products

Special Data Products are data products which are not produced from routine processing. There are two REASON Special Data Products: (i) EIS-derived reprojected radargram image and (ii) *ranging*. There are two processing modules used to generate these special data products: (i) EIS-derived reprojected radargram clutter discrimination module and (ii) *ranging* module. The EIS-derived reprojected radargram image requires data from EIS and will have to be processed manually. The ranging product requires human intervention to select appropriate radar returns.

### Browse Data Product

An individual Browse Data Product is produced for each Derived Data Product. These REASON Browse Data Products are meant to allow end users of REASON data to easily assess if the corresponding data set is potentially useful to their scientific goals and serve as a human-interpretable representation of the data set. As such, Browse Data Products are not meant to be the foundation on which scientific analyses based on REASON data are built. Browse Data Products corresponding to profile Derived Data Products (i.e., TEC, *altimetry*, and *reflectometry* profiles) are presented as two-dimensional images. Each image presents a colored nadir REASON groundtrack overlain on top of a Europa image mosaic. Colors along the groundtrack represent the values in the specific Derived REASON Data Product being presented. A separate image is provided for each panel crossed by the groundtrack.

## Beyond REASON Guiding and Extended Science

### Future Work to Support REASON Guiding Science

Future work, including laboratory, analog, and synergistic studies, will be necessary to support timely interpretation of REASON observations during the prime mission.

#### Laboratory Experiments

The dielectric properties of pure ice have been measured over a range of cryogenic temperatures (Matsuoka et al. 1996 and references therein). However, differences in experimental procedures and sample properties represent a significant source of uncertainty (see review in Pettinelli et al. 2015). The presence of non-ice impurities and/or void space further alters these properties. Mixing models are often employed to represent the effective permittivity of materials composed of multiple components (Sihvola 2013). Classical mixing models assume that the scale of inclusions in a matrix is small relative to the wavelength and inclusions are homogeneously distributed isotropic spheres (Sihvola 2013). More complex mixing models which incorporate geometry and anisotropy exist but are not typically used by the planetary community. Recent laboratory experiments motivated by ocean worlds exploration have focused on constraining the dielectric properties of salt hydrates, specifically meridianiite (MgSO$_{4}\cdot $11H_2_O) (Pettinelli et al. 2016; Grimm and Stillman 2019).

The dielectric properties of Europa’s near-surface are likely dominantly governed by three phases: ice, void space, and salt hydrates. However, at depths/temperatures where brine is thermodynamically stable, brine will likely represent the dominant non-ice contribution to the effective permittivity. Unlike salt hydrates, which have a negligible electrical conductivity (Pettinelli et al. 2016; Grimm and Stillman 2019), the electrical conductivity of these brines could represent a significant contribution to the effective permittivity of the bulk ice (Stillman et al. 2018). Future laboratory experiments to support interpretation of REASON observations should focus on constraining dielectric properties of these multiphase, multicomponent materials, specifically (i) evaluating the applicability of different mixing models for representing hypothesized mixtures, (ii) investigating the dependence on the volume fraction and geometry of brine inclusions, and (iii) considering a broader range of non-ice impurities including clathrates and sulfuric acid hydrates.

#### Terrestrial and Planetary Analog Studies

##### Sub-Ice Hypersaline Systems

Ice-penetrating radar data have been used to identify putative subglacial hypersaline systems beneath ice caps on Earth (Rutishauser et al. 2018, 2022) and on Mars (Orosei et al. 2018). These systems are attributed to basal reflectors that are both highly reflective (suggesting the presence of water) and coincident with estimated bed temperatures that are too low for water to be thermodynamically stable. However, because apparent reflectivity is governed by the dielectric contrast, interface roughness, and attenuation to the interface — interpretation is challenged by uncertainties in both interface characteristics and ice column properties. This ambiguity has been emphasized in recent publications which have been proposing alternative hypotheses to subglacial hypersaline systems as the source of the radar-bright regions beneath the southern polar cap of Mars (e.g., Bierson et al. 2021; Schroeder and Steinbrügge 2021; Smith et al. 2021; Grima et al. 2022a; Lalich et al. 2022). Some of these alternative hypotheses have in turn been claimed to be either unphysical (Mattei et al. 2022) or incapable of producing the observed signal (Orosei et al. 2022; Stillman et al. 2022; Lauro et al. 2022; Cosciotti et al. 2023). On Earth, ice-penetrating radar data sets can be complemented by other geophysical data sets (e.g., seismic, gravity, magnetics) or additional ice-penetrating radar data collected using systems with different bandwidths and/or center frequencies (Chu et al. 2021; Chan et al. 2023b), to improve interpretation. However, REASON will be the only investigation on Europa Clipper capable of direct sensing of the ice shell interior. As such, the development of more robust approaches for analyzing radar data acquired over putative hypersaline subglacial water systems on Earth is essential for interpretation of future data collected by REASON.

##### Accreted Ice

Ice shelves and glaciers represent important analogs for ice-penetrating radar studies of Europa. Although the environmental conditions at Europa and Earth’s cryosphere are generally not directly comparable, there are shared characteristics that will produce similar responses when studied with an ice-penetrating radar. Beneath the ice shelves, seawater can freeze onto the base forming “accreted ice”. The physical processes that govern this freeze-on may occur at Europa’s ice–ocean interface, incorporating salt into the ice shell (Buffo et al. 2020; Wolfenbarger et al. 2022a). These salts alter the dielectric properties of pure ice (Pettinelli et al. 2016), suggesting accretion could serve as a mechanism to introduce dielectric contrasts into the ice shell. On Earth, marine ice layers at the base of meteoric ice shelves can manifest as internal reflectors that precede and often mask the ice–ocean interface (Sect. 1.3, Fig. 5). These marine–meteoric interfaces could be considered a radar-analog for eutectic interfaces within Europa’s ice shell. As such, understanding how properties of these interfaces on Earth influence the character of the radar reflection will improve interpretation of analogous reflectors at Europa.

### REASON Complementary Science

Europa Science beyond the formal Science Requirements and Planning Guidelines presented above (Table 1) is often a focus of discussion for the Europa Clipper Science Team. Three examples are presented below as a powerful indicator of the potential impact of the REASON science investigations beyond the established definition of Guiding and Extended Science with the caveat that this science is opportunistic and not required by the mission.

#### Landing Site Reconnaissance

It is the standard scheme of planetary exploration that an in-situ investigation of the surface by a planetary lander would follow a flagship science spacecraft such as Europa Clipper (e.g., Pappalardo et al. 2013). Although the timeline of such a development is unknown, Europa Clipper is expected to be the mission that will provide the reconnaissance data set to support the selection of a future landing site (Phillips et al. 2024). Consequently, observations from Europa Clipper must be capable of providing physical characterization of the surface to ensure a safe deployment of a probe, as well as assessment of the target science value for a follow-on lander.

REASON will mainly contribute to reconnaissance goals through its *sounding*, *altimetry* and *reflectometry* measurements. *Sounding* measurements will support the regional science assessment of the landing area by providing insights into the subsurface structure, including possible thermal boundaries and compositional contacts (e.g., salt layers). In contrast to *sounding*, REASON *altimetry* measurements are solely focused on determining the relative distance from the Europa Clipper spacecraft to Europa’s surface using the higher vertical resolution (15 m in free space) VHF surface echoes. *Altimetry* measurements will complement visible stereoscopy by providing contextual surface topography along the Europa Clipper groundtracks. The combination of REASON *sounding* and *altimetry* data will also be used to search for the presence of liquid water either within or at the base of the ice shell by way of isostasy. The detection of zones of liquid water within the ice shell will be relevant to a future Europa lander as it signifies an area of on-going geological activity and may host a potentially habitable, near-surface environment. *Reflectometry* measurements of the surface echo at the two REASON frequencies will provide insights to dominant surface and near-surface properties (e.g., roughness) within the radar footprint, to depths of 15 m to 150 m, that are directly relevant to both landing science and risk assessment. That includes porosity (Grima et al. 2014a), roughness (Grima et al. 2014b), fallout-deposit from recent plume activity (Scanlan et al. 2022), near-surface refrozen brines (Rutishauser et al. 2016; Chan et al. 2023b), or the presence of near-surface liquid water potentially accessible to future in-situ sampling (Grima et al. 2016).

#### JUICE/RIME Synergy

JUICE will operate in the Jovian system concurrent with Europa Clipper. One of JUICE’s primary mission objectives is to make detailed observations of Ganymede, but it will also collect measurements at Europa and Callisto during its initial orbital insertion phases. The JUICE payload includes RIME (Bruzzone et al. 2011), a 9-MHz sister instrument to REASON HF. RIME and REASON overlapping targets and similar instrumental parameters, offer a unique opportunity for synergistic science. Both instruments will have common observations at intersections, allowing radiometric calibration of their respective measurements. The outcome is to produce a radiometrically homogeneous 9-MHz *reflectometry* and *sounding* dataset covering Europa, Ganymede, and Callisto (a first of its kind in planetary exploration), which could be used to relatively compare signal attenuation and scattering from the various englacial and surface structures encountered across the Jovian icy moons. RIME can also set its transmitted bandwidth to either 1 MHz (same as REASON) or 3 MHz. Because surface roughness is wavelength-dependent, observations with REASON and RIME at the same center frequency (9 MHz) but different bandwidths could help to isolate the effects of surface roughness in order to better characterize bulk near-surface properties (e.g., layering) over various types of terrains (Chan et al. 2023a). Assuming the effects of roughness are either negligible, analogous to internal layers in glacial ice (e.g., MacGregor et al. 2015 and references therein), or can be quantified and corrected (e.g., Grima et al. 2019), multi-frequency/bandwidth analyses could also be used to better characterize plume fallout deposits at the surface (Scanlan et al. 2022) or salt layers at depth (Wolfenbarger et al. 2023). Finally, the concurrent presence of JUICE and Europa Clipper in the Jovian system can help to better characterize its magnetospheric plasma where both radars could be used as transmitters-receivers of each other signal, the relative delay and attenuation of which are indicative of the plasma density along the propagation path.

#### Passive Radar Sounding

REASON has the capability to perform passive sounding using signals of opportunity originating from the Jovian magnetosphere (Cecconi et al. 2012; Romero-Wolf et al. 2015; Schroeder et al. 2016). In this mode of operation, REASON would not be transmitting a signal, but would record incoming Jovian emissions and their possible reflections from the surface and subsurface of Europa. A processing step analogous to range-compression would then reveal geological features as in standard, active sounding (Gerekos et al. 2020). Jovian decametric emissions are coherent and highly beamed, making them good candidates for passive sounding (Carrer et al. 2021; Gassot et al. 2022). These characteristics could similarly be favorable for estimating TEC and studying the ionosphere (Peters et al. 2020). The use of passive sounding could complement the standard concept of operations on the sub-Jovian side of Europa in a high-noise environment. While the spatial extent of solar radio-sources likely prevents their use for passive subsurface sounding (Peters et al. 2022), passive observations of solar bursts from the outer solar system can be envisioned. Recent re-analyses of archival SHARAD and MARSIS data have shown that radar sounders have the capability to image type-III bursts as high resolution from Martian orbit (Gerekos et al. 2024), and such multi-point observations can inform about the processes behind burst generation (Jebaraj et al. 2020) and their propagation across interplanetary space (Reid and Kontar 2021).

## Summary

REASON is a combined HF/VHF ice penetrating radar specifically conceived, developed, and operated to provide a top-to-bottom window into many of the most critical and poorly understood processes and conditions governing the behavior, evolution, and habitability of Europa’s ice shell. At the highest level, REASON is designed to probe the exosphere, the surface/near-surface, the shallow subsurface to three kilometers and the full depth of Europa’s ice shell to an ice–ocean interface at depths as great as thirty kilometers with nadir-directed active radar signals centered at both 9 MHz and 60 MHz. This pair of frequencies provides both robustness (with the 9 MHz band less sensitive to surface roughness and volume scattering and the 60 MHz band less sensitive to Jovian radio noise) and richness (capturing cross-frequency signatures of ionosphere and ice-shell properties and processes) to the full suite of Europa Clipper radar assessment and sounding investigations. Similarly, the unique dual-channel, cross-track interferometric capability of REASON’s VHF band allows for potentially obfuscating cross-track surface clutter with similar delays as subsurface signals to be discriminated internally to the instrument, increasing the fidelity and reliability of radargram interpretation. REASON will be calibrated through carefully coordinated combinations of transmitting and receiving HF and VHF before and after Jupiter orbit insertion, d. With these calibrations, REASON will be operated and its data analyzed to explore physical constraints on the potential habitability of Europa through a full suite of *altimetry*, *reflectometry*, *sounding*, *interferometry*, *plasma characterization*, and *ranging* measurements—each described briefly below with their implications for REASON guiding and extended science (Table 2) as well as complementary science.

Surface returns from the REASON VHF (with ionospheric-delay corrections provided by concurrent HF surface returns) are a significant but often underappreciated observation at Europa, representing the primary *altimetry* measurement for the Europa Clipper payload. This *altimetry* measurement will be used, in combination with EIS stereo imagery, to provide registration and measurement of the surface geometry of Europa which in turn provides a framework for critical hypothesis tests of floatation from any shallow subsurface water bodies. In addition to providing context for both interior ice shell processes and their expression in surface geology through *altimetry*, REASON near-surface *reflectometry* will provide a quantitative assessment of Europa’s surface roughness and near-surface material properties (including brines, salt layers, porosity, and plume deposits). *Reflectometry* measurements will also provide wavelength-scale statistical constraints on surface roughness, which are also critical for landing-zone reconnaissance. As a *sounding* instrument, REASON will be able to probe both shallow (to three kilometers) and deeper subsurface processes of Europa’s ice shell (up to thirty kilometers) by exploiting the sensitivity of HF and VHF (assisted by *interferometry*) radar signals to both the compositional and geometric character of the shallow subsurface (e.g. water and salt bodies) as well as the thermophysical structure (e.g. temperature, brine pockets) across the full depth of the ice shell potentially including the ice-ocean interface or its thermophysical proxy. The detection and characterization of reflectivity, attenuation, and scattering signatures in REASON *sounding* data at intersections, along profiles, and across panels will place unique constraints on subsurface properties and processes such as the depth of pore-closure, the existence of eutectic horizons, shallow and relict water bodies, and the thickness and salinity of the ice shell and ocean. The global distribution of REASON VHF returns from *ranging* between the spacecraft and the surface at flyby crossovers compared with simulated surface returns (from EIS digital elevation models including both returns at nadir and off-nadir “clutter”) will provide the opportunity to place additional constraints on tidal deformation as part of larger, mission-wide, geodetic inversions to constrain ice-shell and ocean thickness. The combination of REASON HF and VHF surface returns provide a critical *plasma characterization* measurement that will not only allow for ionospheric correction of *altimetry* and *ranging* measurements but also provide essential constraints on the nadir TEC, which will enable both better characterization of the ionosphere and local plume detection. Taken together, the REASON investigation and instrument are designed to enable a unique suite of radar observations and analyses to test hypotheses and reveal processes, by assessing and sounding Europa from the ocean to the near-surface of the ice shell.

## Appendix: Ensemble of Point Models (EPM) Tables

Tables 12–14 present the regime modes assumed in the construction of the point models described in Sect. 4.3. For point models in parentheses, salt layers represent the target interface, as opposed to the subsurface water interface.

**Table 12:** Shallow Subsurface Structure EPM

Regime	SS01(07)	SS02(08)	SS03(09)	SS04(10)	SS05(11)	SS06(12)
Surface Roughness	Ridged Plains	Chaotic Terrain	Chaotic Terrain	Ridged Plains	Ridged Plains	Chaotic Terrain
Near-Surface Density	Non-Porous					
Near-Surface Thickness	Thin					
Near-Surface Composition	Clean Ice					
Off-Nadir Surface Feature	Ribbons & Curbs					
Deep Regolith	Absent	Present	Absent	Absent	Absent	Absent
Surface Temperature	Polar & Equatorial					
Ice Shell Composition	Marine	Pure	Pure	Marine	Pure	Marine
Ice Shell Thickness	Thin					
Subsurface Water Interface	Sharp	Sharp	Sharp	Mush	Sharp	Sharp
Basal Temperature	Melting	Melting	Melting	Eutectic	Melting	Melting
(Salt Layer Thickness)	1 m					
(Salt Layer Depth)	1/2 Ice Shell Thickness

**Table 13:** Full Depth Subsurface Exchange EPM

Regime	FD01(07)	FD02(08)	FD03(09)	FD04(10)	FD05(11)	FD06(12)
Surface Roughness	Ridged Plains	Chaotic Terrain	Chaotic Terrain	Ridged Plains	Ridged Plains	Chaotic Terrain
Near-Surface Density	Non-Porous					
Near-Surface Thickness	Thin					
Near-Surface Composition	Clean Ice					
Off-Nadir Surface Feature	Ribbons & Curbs					
Deep Regolith	Absent	Present	Absent	Absent	Absent	Absent
Surface Temperature	Polar & Equatorial					
Ice Shell Composition	Marine	Pure	Pure	Pure	Pure	Marine
Ice Shell Thickness	Thin	Thin	Thin	Thick	Thick	Thick
Subsurface Water Interface	Mush	Mush	Mush	Sharp	Mush	Sharp
Basal Temperature	Eutectic	Eutectic	Eutectic	Melting	Eutectic	Melting
(Salt Layer Thickness)	1 m	1 m	1 m	1 m	1 m	0.2 m
(Salt Layer Depth)	1/2 Ice Shell Thickness					10 km

**Table 14:** Altimetry Reflectometry EPM

Regime	AR01	AR02	AR03	AR04	AR05	AR06
Surface Roughness	Ridged Plains	Chaotic Terrain	Ridged Plains	Ridged Plains	Chaotic Terrain	Ridged Plains
Near-Surface Density	Non-Porous	Non-Porous	Porous	Non-Porous	Non-Porous	Porous
Near-Surface Thickness	Thick	Thick	Thick	Thick	Thick	Thin
Near-Surface Comp.	Clean Ice	Clean Ice	Clean Ice	Brine-Filled Ice	Brine-Filled Ice	Clean Ice
Off-Nadir Surface Feature	–	–	–	–	–	–
Deep Regolith	–	–	–	–	–	–
Surface Temperature	–	–	–	–	–	–
Ice Shell Composition	–	–	–	–	–	–
Ice Shell Thickness	–	–	–	–	–	–
Subsurface Water Interface	–	–	–	–	–	–
Basal Temperature	–	–	–	–	–	–
(Salt Layer Thickness)	–	–	–	–	–	–
(Salt Layer Depth)	–	–	–	–	–	–

## Footnotes

1 Habitability in this context refers to the conditions necessary to support life as we know it (see Vance et al. 2023, this collection).

2 Here we use the terms radar sounder and ice-penetrating radar to refer specifically to pulse radar systems on airborne or space-based platforms (Schlegel et al. 2022). Radar sounder is the term typically used in planetary radioglaciology, whereas Radio Echo Sounder (RES) and ice-penetrating radar are terms commonly used in terrestrial radioglaciology.

3 Formally, near-surface refers to depths <300 m, shallow subsurface refers to depths ≤3 km, and full depth subsurface refers to depths from the surface to ≤30 km. The shallow and full depth subsurface depths are defined consistent with the thin and thick ice shell modes described in Sect. 4.2.6. The near-surface is defined as the region close to the surface where subsurface structure cannot be directly resolved by the radar (i.e., the vertical resolution). In practice, the vertical resolution is governed by the bandwidth, windowing function, and dielectric properties of the material; however, here we adopt the required HF vertical resolution in ice as a conservative upper bound (Table 2).

4 REASON’s *ranging* measurement technique should not be confused with ranging as used by Gravity/Radio Science (see Mazarico et al. 2023, this collection). The term *ranging* was adopted to distinguish the measurement technique from *altimetry*.

5 Permittivity is a complex quantity which describes the polarizability of a material (i.e., energy storage and dissipation in response to an applied electromagnetic field) (Sihvola 1999).

6 The Fresnel zone is defined as the distance over which the phase of echoes from the central nadir point change by $\pi /2$ radians (Peters et al. 2005b).

7 The term eutectic is used to refer to the interface defined by the minimum temperature at which ice transitions from solid to molten, resulting from the presence of impurities which depress the freezing point (Kalousová et al. 2017; e.g., Culha et al. 2020). The eutectic temperature is governed by the composition of non-ice impurities (Wolfenbarger et al. 2022b).

8 Lenticulae are small circular or elliptical dark spots corresponding to topographic lows (pits) or highs (domes) (Doggett et al. 2009).

9 Cluttergrams are synthetic radargrams generated from DEMs which are used to determine if nadir subsurface interfaces can be explained by energy reflecting from off-nadir surface features (e.g., Choudhary et al. 2016).

10 In radar sounding data, side lobes are peaks (lobes) that appear adjacent to the main peak in a reflected signal resulting from pulse compression of the transmitted chirp waveform.

11 The term “interfero-metric” was adopted to convey that this KRP is a metric used to verify the REASON measurement technique of *interferometry*, hence the distinct spelling from the adjective interferometric.

12 SPICE is an information system Navigation and Ancillary Information Facility (NAIF) to support planetary exploration missions by providing data sets known as “kernels” that contain information including ephemerides of spacecraft and planetary bodies as a function of time (Acton et al. 2018).

## Acronyms and Abbreviations

A/D: Analog/Digital
Actr: Actuator
ADC: Analog-to-Digital Converter
ALSE: Apollo 17 Lunar Sounder Experiment
ANT: Antenna
APL: Applied Physics Laboratory
AS: Antenna Subsystem
AS-Mech: Antenna Subsystem – Mechanical
ATLO: Assembly, Test, Launch and Operations
ATTN: Attenuation
AWG: Arbitrary Waveform Generator
BDS: Bulk Data Storage
BFPQ: Block Floating-Point Quantization
B/H: Ratio of magnetic flux density (B) to magnetic field strength (H)
CA: Closest Approach
Caltech: California Institute of Technology
CBE: Current Best Estimate
CDH: Command and Data Handling
CLK: Clock
Coax: Coaxial cable
COTS: Commercial Off-the-Shelf
CRF: Cycle Repetition Frequency
CSV: Comma-Separated Value
CTRL/TLM: Control/Telemetry
CTRL/TMG: Control/Timing
CTU: Control and Timing Unit
D/A: Digital/Analog
DAC: Digital-to-Analog Converter
DAD: Deployment Assist Device
DC: Direct Current
DEM: Digital Elevation Model
DES: Digital Electronic Subsystem
DMM: Digital Multimeter
DTM: Digital Terrain Model
DPU: Digital and Power Unit
DQA: Data Quality Analyzer
DSE: Digital and Synthesizer Electronics
DSEPCU: DSE Power Converter Unit
DSES: Digital and Synthesizer Electronics Stacks
DSN: Deep Space Network
E-THEMIS: Europa Thermal Emission Imaging System
ECM: Europa Clipper Magnetometer
EGSE: Electronic Ground Support Equipment
EIS: Europa Imaging System
EM: Engineering Module
EMC: Electromagnetic Compatibility
EMI: Electromagnetic Interference
EMI/EMC: Electromagnetic Interference and Compatibility
EPM: Ensemble of Point Models
EPS: Experiment Planning System
ESA: European Space Agency
Europa-UVS: Europa Ultraviolet Spectrograph
FDVHF: Full Depth Very High Frequency
FET: Field-Effect Transistor
FJ: Field Joint
Flt Sys: Flight System
FM: Flight Module
FODL: Fiber Optic Delay Line
FPGA: Field-Programmable Gate Array
Frang: Frangibolt
FrngActuator: Frangibolt Actuator
Func Gen: Function Generator
GaN: Gallium Nitride
GPS: Global Positioning System
GSE: Ground Support Equipment
GSFC: Goddard Space Flight Center
H&S: Health and Status
HAAMX: High Frequency Antenna Assembly -X
HAAPX: High Frequency Antenna Assembly +X
HD: Hard Drive
HF: High Frequency
HFPAU: High Frequency Power Amplifier Unit
HFPSU: High Frequency Power Supply Unit
HFRx: High Frequency Receivers Assembly
HFS: High Frequency Electronic Stack
HFTxDA: High Frequency Transmit Distribution Assembly
HW: Hardware
I/F: Interface
I&O: Inner and Outer
I&T: Integration-and-Test
IC: Integrated Circuit
IEP: Initial Encounter Plan
IESD: Internal Electrostatic Discharge
II&T: Instrument Integration-and-Test
Instr: Instrument
IOS: Instrument Operations System
iSDS: Instrument Science Data System
ISOC: Instrument Science Operations Center
iST: Instrument Science Team
Itfc: Interface
JOI: Jupiter Orbit Insertion
JPL: Jet Propulsion Laboratory
JUICE: JUpiter ICy moons Explorer
KRP: Key Radar Parameter
LEP: Leading Edge Position
LRS: Lunar Radar Sounder
LVDS: Low-Voltage Differential Signaling
m-SDS: Mission Science Data System
magamp: Magnetic Amplifier
MARSIS: Mars Advanced Radar for Subsurface and Ionospheric Sounding
MASPEX: MAss Spectrometer for Planetary EXploration
Meas.: Measurement
MISE: Mapping Imaging Spectrometer for Europa
MN: Matching Network
MOS: Mission Operations System
MRAM: Magnetoresistive Random-Access Memory
MRAM_SEL: Magnetoresistive Random-Access Memory Select
MRO: Mars Reconnaissance Orbiter
MX: -X
MXI: -X Inner
MXO: -X Outer
NAIF: Navigation and Ancillary Information Facility
NASA: National Aeronautics and Space Administration
NSLE: Normalized Side Lobe Envelope
O-scope: Oscilloscope
OBP: On-Board Processor
OP: Observation Plan
OTM: Orbit Trim Maneuver
OVRO: Owens Valley Radar Observatory
OVRO-LWA: Owens Valley Radar Observatory Long Wavelength Array
PAU: Power Amplification Unit
PA: Power Amplifier
PC: Personal Computer
PCE: Planning, Coordination, and Execution Operations
PCDA: Power Control Distribution Assembly
PCU: Power Converter Unit
PDS: Planetary Data System
PEEK: Polyetheretherketone
PI: Principal Investigator
PIMS: Plasma Instrument for Magnetic Sounding
PLL: Phase Locked Loop
PME: Propulsion Module Electronics
PPDP: Partially Processed Data Product
PRF: Pulse Repetition Frequency
Prop: Propulsion
PRT: Platinum Resistance Thermometer
PSS: Power Switch Slice
PSU: Power Supply Unit
PWM: Pulse-Width Modulation
PWR: Power
PX: +X
PX/MX: +X/-X
PXI: +X Inner
PXO: +X Outer
RAMBo: Radar Acquisition & Management Board
RAP: Reference Activity Plan
RAW: Raw Data Product
REASON: Radar for Europa Assessment and Sounding: Ocean to Near-surface
REU: Remote Engineering Unit
RF: Radio Frequency
RFES: Radio Frequency Electronics System
RFES-JPL: Radio Frequency Electronics System Jet Propulsion Laboratory
RFI: Radio Frequency Interference
RFPSU: Radio Frequency Power Supply Unit
RIME: Radar for Icy Moons Exploration
Rx: Receiver
S/C: Spacecraft
SA: Solar Arrays
SAR: Synthetic Aperture Radar
SC/PropMod: Spacecraft Propulsion Module
Sci Sys: Science System
SCOS: Spacecraft Operations Subsystem
SDS: Science Data System
SELENE: SELenological and ENgineering Explorer
SEG: Segment
SHARAD: SHAllow RADar
Sig Gens: Signal Generators
Sim: Simulator
SIOS: Science and Instrument Operations Subsystem
SNR: Signal-to-Noise Ratio
Spec-A: Spectrum Analyzer
SPLD: South Polar Layered Deposits
SPOC: Science Planning Operations Coordination
SpW: SpaceWire
SRAM: Static Random Access Memory
SSPG: Strategic Science Planning Guide
STACER: Spiral Tube and Actuator for Controlled Extension and Retraction
STALO: STAble Local Oscillator
STB: System Testbed
STEREO: Solar Terrestrial Relations Observatory
SUDA: SUrface Dust Analyzer
SVV: Science Verification and Validation
Synth: Synthesizer Assembly
T/R: Transmit/Receive
TEC: Total Electron Content
Temp: Temperature
TID: Total Ionizing Dose
TLM: Telemetry
TT: Telltale
TT/T: Telltale/Temps
TTL: Transistor–transistor logic
TVAC: Thermal Vacuum
Tx: Transmit
Tx/Rx: Transmit/Receive
TxDA: Transmit Driver Assembly
UTIG: University of Texas Institute for Geophysics
V&V: Verification and Validation
VAA: Very High Frequency Antenna Assembly
VAAMXI: Very High Frequency Antenna Assembly -X Inner
VAAMXI/O: Very High Frequency Antenna Assembly -X Inner/Outer
VAAMXO: Very High Frequency Antenna Assembly -X Outer
VAAPXI: Very High Frequency Antenna Assembly +X Inner
VAAPXI/O: Very High Frequency Antenna Assembly +X Inner/Outer
VAAPXO: Very High Frequency Antenna Assembly +X Outer
VDD: Positive supply voltage
VHF: Very High Frequency
VHFPAU: Very High Frequency Power Amplifier Unit
VHFPSU: Very High Frequency Power Supply Unit
VHFRx: Very High Frequency Receivers Assembly
VHFS: Very High Frequency Electronic Stack
VHFTxDA: Very High Frequency Transmitters Distribution Assembly
WBS: Work Breakdown Structure

## References

[CR1] Abramov O, Spencer JR (2008) Numerical modeling of endogenic thermal anomalies on Europa. Icarus 195:378–385. 10.1016/j.icarus.2007.11.027 10.1016/j.icarus.2007.11.027

[CR2] Abramov O, Rathbun JA, Schmidt BE, Spencer JR (2013) Detectability of thermal signatures associated with active formation of ‘chaos terrain’ on Europa. Earth Planet Sci Lett 384:37–41. 10.1016/j.epsl.2013.09.027 10.1016/j.epsl.2013.09.027

[CR3] Acton C, Bachman N, Semenov B, Wright E (2018) A look towards the future in the handling of space science mission geometry. Planet Space Sci 150:9–12. 10.1016/j.pss.2017.02.013 10.1016/j.pss.2017.02.013

[CR4] Aglyamov Y, Schroeder DM, Vance SD (2017) Bright prospects for radar detection of Europa’s ocean. Icarus 281:334–337. 10.1016/j.icarus.2016.08.014 10.1016/j.icarus.2016.08.014

[CR5] Alexander C, Carlson R, Consolmagno G, Greeley R, Morrison D (2009) The exploration history of Europa. In: Pappalardo RT, McKinnon WB, Khurana KK (eds) Europa. University of Arizona Press, Tucson, pp 3–26. 10.2307/j.ctt1xp3wdw.7

[CR6] Anderson J, Schubert G, Jacobson R, Lau E, Moore W, Sjogren W (1998) Europa’s differentiated internal structure: inferences from four Galileo encounters. Science 281:2019–2022 10.1126/science.281.5385.2019 9748159 10.1126/science.281.5385.2019

[CR7] Ashkenazy Y (2019) The surface temperature of Europa. Heliyon 5:e01908. 10.1016/j.heliyon.2019.e01908 31294099 10.1016/j.heliyon.2019.e01908 PMC6595243

[CR8] Ashmore DW, Bingham RG, Hindmarsh RCA, Corr HFJ, Joughin IR (2014) The relationship between sticky spots and radar reflectivity beneath an active West Antarctic ice stream. Ann Glaciol 55:29–38. 10.3189/2014AoG67A052 10.3189/2014AoG67A052

[CR9] Bagenal F, Dols V (2020) The space environment of Io and Europa. J Geophys Res Space Phys 125:e2019JA027485. 10.1029/2019JA027485 10.1029/2019JA027485

[CR10] Bagenal F, Wilson RJ, Siler S, Paterson WR, Kurth WS (2016) Survey of Galileo plasma observations in Jupiter’s plasma sheet. J Geophys Res, Planets 121:871–894. 10.1002/2016JE005009 10.1002/2016JE005009

[CR11] Bagenal F, Dougherty L, Bodisch K, Richardson J, Belcher J (2017) Survey of Voyager plasma science ions at Jupiter: 1. Analysis method. J Geophys Res Space Phys 122:8241–8256. 10.1002/2016JA023797 10.1002/2016JA023797

[CR12] Bailey J, Evans S, Robin GdQ (1964) Radio echo sounding of polar ice sheets. Nature 204:420–421. 10.1038/204420a0 10.1038/204420a0

[CR13] Barr AC, Showman AP (2009) Heat transfer in Europa’s icy shell. In: Pappalardo RT, McKinnon WB, Khurana KK (eds) Europa. University of Arizona Press, Tucson, pp 405–430. 10.2307/j.ctt1xp3wdw.23

[CR14] Becker HN, Lunine JI, Schenk PM, Florence MM, Brennan MJ, Hansen CJ, Martos YM, Bolton SJ, Alexander JW (2023) A complex region of Europa’s surface with hints of recent activity revealed by Juno’s Stellar Reference Unit. J Geophys Res, Planets 128:e2023JE008105. 10.1029/2023JE008105 10.1029/2023JE008105

[CR15] Bentley CR (1987) Antarctic ice streams: a review. J Geophys Res, Solid Earth 92:8843–8858. 10.1029/JB092iB09p08843 10.1029/JB092iB09p08843

[CR16] Bentley C, Blankenship D, Moline G (1988) Electromagnetic studies on the Siple Coast, 1987–1988. Antarct J U S 23:59–59

[CR17] Bierhaus EB, Zahnle K, Chapman CR (2009) Europa’s crater distributions and surface ages. In: Pappalardo RT, McKinnon WB, Khurana KK (eds) Europa. University of Arizona Press, Tucson, pp 161–180. 10.2307/j.ctt1xp3wdw.13

[CR18] Bierson CJ, Tulaczyk S, Courville SW, Putzig NE (2021) Strong MARSIS radar reflections from the base of Martian south polar cap may be due to conductive ice or minerals. Geophys Res Lett 48:e2021GL093880. 10.1029/2021GL093880 10.1029/2021GL093880

[CR19] Billings SE, Kattenhorn SA (2005) The great thickness debate: ice shell thickness models for Europa and comparisons with estimates based on flexure at ridges. Icarus 177:397–412. 10.1016/j.icarus.2005.03.013 10.1016/j.icarus.2005.03.013

[CR20] Black GJ, Campbell DB, Nicholson PD (2001) Icy Galilean satellites: modeling radar reflectivities as a coherent backscatter effect. Icarus 151:167–180. 10.1006/icar.2001.6616 10.1006/icar.2001.6616

[CR21] Blankenship D, Edwards B, Kim Y, Geissler P, Gurnett D, Johnson W, Kofman W, Moore J, Morse D, Pappalardo R (1999) Feasibility study and design concept for an orbiting ice-penetrating radar sounder to characterize in three-dimensions the Europan ice mantle down to (and including) any ice/ocean interface. Institute for Geophysics

[CR22] Blankenship D, Morse D, Finn C, Bell R, Peters M, Kempf S, Hodge S, Studinger M, Behrendt JC, Brozena J (2001) Geologic controls on the initiation of rapid basal motion for West Antarctic ice streams: a geophysical perspective including new airborne radar sounding and laser altimetry results. West Antarct Ice Sheet Behav Environ 77:105–121. 10.1029/AR077p0105 10.1029/AR077p0105

[CR23] Blankenship DD, Young DA, Moore WB, Moore JC (2009) Radar sounding of Europa’s subsurface properties and processes: the view from Earth. In: Pappalardo RT, McKinnon WB, Khurana KK (eds) Europa. University of Arizona Press, Tucson, pp 631–654. 10.2307/j.ctt1xp3wdw.33

[CR24] Brown W Jr, Adams G, Eggleton R, Jackson P, Jordan R, Kobrick M, Peeples W, Phillips R, Porcello L, Schaber G (1974) Elevation profiles of the Moon. In: Proceedings of the Fifth Lunar Conference. vol 3, pp 3037–3048

[CR25] Brown G (1977) The average impulse response of a rough surface and its applications. IEEE Trans Antennas Propag 25:67–74. 10.1109/TAP.1977.1141536 10.1109/TAP.1977.1141536

[CR26] Brown DW (2021) The mission: a true story. Harper Collins, New York

[CR27] Brown M, Hand K (2013) Salts and radiation products on the surface of Europa. Astron J 145:110. 10.1088/0004-6256/145/4/110 10.1088/0004-6256/145/4/110

[CR28] Bruzzone L, Alberti G, Catallo C, Ferro A, Kofman W, Orosei R (2011) Subsurface radar sounding of the Jovian moon Ganymede. Proc IEEE 99:837–857. 10.1109/JPROC.2011.2108990 10.1109/JPROC.2011.2108990

[CR29] Bruzzone L, Plaut JJ, Alberti G, Blankenship DD, Bovolo F, Campbell BA, Ferro A, Gim Y, Kofman W, Komatsu G, McKinnon W, Mitri G, Orosei R, Patterson GW, Plettemeier D, Seu R (2013) RIME: radar for icy moon exploration. In: 2013 IEEE international geoscience and remote sensing symposium - IGARSS, pp 3907–3910. 10.1109/IGARSS.2013.6723686

[CR30] Buffo JJ, Schmidt BE, Huber C, Walker CC (2020) Entrainment and dynamics of ocean-derived impurities within Europa’s Ice Shell. J Geophys Res, Planets 125:e2020JE006394. 10.1029/2020JE006394 10.1029/2020JE006394

[CR31] Campbell BA (2009) Scale-dependent surface roughness behavior and its impact on empirical models for radar backscatter. IEEE Trans Geosci Remote Sens 47:3480–3488. 10.1109/TGRS.2009.2022752 10.1109/TGRS.2009.2022752

[CR32] Campbell BA, Morgan GA (2018) Fine-scale layering of Mars polar deposits and signatures of ice content in nonpolar material from multiband SHARAD data processing. Geophys Res Lett 45:1759–1766. 10.1002/2017GL075844 10.1002/2017GL075844

[CR33] Campbell BA, Watters TR (2016) Phase compensation of MARSIS subsurface sounding data and estimation of ionospheric properties: new insights from SHARAD results. J Geophys Res, Planets 121:180–193. 10.1002/2015JE004917 10.1002/2015JE004917

[CR34] Campbell BA, Putzig NE, Carter LM, Phillips RJ (2011) Autofocus correction of phase distortion effects on SHARAD echoes. IEEE Geosci Remote Sens Lett 8:939–942. 10.1109/LGRS.2011.2143692 10.1109/LGRS.2011.2143692

[CR35] Campbell BA, Putzig NE, Carter LM, Morgan GA, Phillips RJ, Plaut JJ (2013a) Roughness and near-surface density of Mars from SHARAD radar echoes. J Geophys Res, Planets 118:436–450. 10.1002/jgre.20050 10.1002/jgre.20050

[CR36] Campbell BA, Putzig NE, Foss FJ Phillips RJ (2013b) SHARAD signal attenuation and delay offsets due to the Martian ionosphere. IEEE Geosci Remote Sens Lett 11:632–635. 10.1109/LGRS.2013.2273396 10.1109/LGRS.2013.2273396

[CR37] Carlson R, Johnson R, Anderson M (1999) Sulfuric acid on Europa and the radiolytic sulfur cycle. Science 286:97–99. 10.1126/science.286.5437.97 10506568 10.1126/science.286.5437.97

[CR38] Carlson R, Anderson M, Johnson R, Schulman M, Yavrouian A (2002) Sulfuric acid production on Europa: the radiolysis of sulfur in water ice. Icarus 157:456–463. 10.1006/icar.2002.6858 10.1006/icar.2002.6858

[CR39] Carnahan E, Wolfenbarger NS, Jordan JS, Hesse MA (2021) New insights into temperature-dependent ice properties and their effect on ice shell convection for icy ocean worlds. Earth Planet Sci Lett 563:116886. 10.1016/j.epsl.2021.116886 10.1016/j.epsl.2021.116886

[CR40] Carnahan E, Vance SD, Cox R, Hesse MA (2022) Surface-to-ocean exchange by the sinking of impact generated melt chambers on Europa. Geophys Res Lett 49:e2022GL100287. 10.1029/2022GL100287 10.1029/2022GL100287

[CR41] Carr MH, Belton MJS, Chapman CR, Davies ME, Geissler P, Greenberg R, McEwen AS, Tufts BR, Greeley R, Sullivan R, Head JW, Pappalardo RT, Klaasen KP, Johnson TV, Kaufman J, Senske D, Moore J, Neukum G, Schubert G, Burns JA, Thomas P, Veverka J (1998) Evidence for a subsurface ocean on Europa. Nature 391:363–365. 10.1038/34857 9450749 10.1038/34857

[CR42] Carrer L, Schroeder DM, Romero-Wolf A, Ries P, Brurzone L (2018) Noise character constraints on passive radio sounding of Jupiter’s icy moons using Jovian decametric radiation. In: IGARSS 2018 - 2018 IEEE international geoscience and remote sensing symposium, pp 4158–4161. 10.1109/IGARSS.2018.8517931

[CR43] Carrer L, Schroeder DM, Romero-Wolf A, Ries PA, Bruzzone L (2021) Analysis of temporal and structural characteristics of Jovian radio emissions for passive radar sounding of Jupiter’s Icy Moons. IEEE Trans Geosci Remote Sens 59:3857–3874. 10.1109/TGRS.2020.3023249 10.1109/TGRS.2020.3023249

[CR44] Cartacci M, Amata E, Cicchetti A, Noschese R, Giuppi S, Langlais B, Frigeri A, Orosei R, Picardi G (2013) Mars ionosphere total electron content analysis from MARSIS subsurface data. Icarus 223:423–437. 10.1016/j.icarus.2012.12.011 10.1016/j.icarus.2012.12.011

[CR45] Carter SP, Blankenship DD, Peters ME, Young DA, Holt JW, Morse DL (2007) Radar-based subglacial lake classification in Antarctica. Geochem Geophys Geosyst 8:Q03016. 10.1029/2006GC001408 10.1029/2006GC001408

[CR46] Cassen P, Reynolds RT, Peale SJ (1979) Is there liquid water on Europa? Geophys Res Lett 6:731–734. 10.1029/GL006i009p00731 10.1029/GL006i009p00731

[CR47] Castelletti D, Schroeder DM, Hensley S, Grima C, Ng G, Young D, Gim Y, Bruzzone L, Moussessian A, Blankenship DD (2017) An interferometric approach to cross-track clutter detection in two-channel VHF radar sounders. IEEE Trans Geosci Remote Sens 55:6128–6140. 10.1109/TGRS.2017.2721433 10.1109/TGRS.2017.2721433

[CR48] Castelletti D, Schroeder DM, Mantelli E, Hilger A (2019) Layer optimized SAR processing and slope estimation in radar sounder data. J Glaciol 65:983–988. 10.1017/jog.2019.72 10.1017/jog.2019.72

[CR49] Cavitte MGP, Blankenship DD, Young DA, Schroeder DM, Parrenin F, Lemeur E, Macgregor JA, Siegert MJ (2016) Deep radiostratigraphy of the East Antarctic Plateau: connecting the Dome C and Vostok ice core sites. J Glaciol 62:323–334. 10.1017/jog.2016.11 10.1017/jog.2016.11

[CR50] Cecconi B, Hess S, Hérique A, Santovito MR, Santos-Costa D, Zarka P, Alberti G, Blankenship D, Bougeret J-L, Bruzzone L, Kofman W (2012) Natural radio emission of Jupiter as interferences for radar investigations of the icy satellites of Jupiter. Planet Space Sci 61:32–45. 10.1016/j.pss.2011.06.012 10.1016/j.pss.2011.06.012

[CR51] Chan K, Grima C, Gerekos C, Blankenship DD (2023a) RIME-REASON synergistic opportunities for surface and near-surface investigations of icy moons. In: EGU General Assembly Conference Abstracts, pp EGU–10554. 10.5194/egusphere-egu23-10554

[CR52] Chan K, Grima C, Rutishauser A, Young DA, Culberg R, Blankenship DD (2023b) Spatial characterization of near-surface structure and meltwater runoff conditions across the Devon Ice Cap from dual-frequency radar reflectivity. Cryosphere 17:1839–1852. 10.5194/tc-17-1839-2023 10.5194/tc-17-1839-2023

[CR53] Chivers CJ, Buffo JJ, Schmidt BE (2021) Thermal and chemical evolution of small, shallow water bodies in Europa’s Ice Shell. J Geophys Res, Planets 126:e2020JE006692. 10.1029/2020JE006692 10.1029/2020JE006692

[CR54] Choudhary P, Holt JW, Kempf SD (2016) Surface clutter and echo location analysis for the interpretation of SHARAD data from Mars. IEEE Geosci Remote Sens Lett 13:1285–1289. 10.1109/LGRS.2016.2581799 10.1109/LGRS.2016.2581799

[CR55] Choukroun M, Molaro JL, Hodyss R, Marteau E, Backes P, Carey EM, Dhaouadi W, Moreland S, Schulson EM (2020) Strength evolution of ice plume deposit analogs of Enceladus and Europa. Geophys Res Lett 47:e2020GL088953. 10.1029/2020GL088953 10.1029/2020GL088953

[CR56] Christensen PR, Spencer JR, Mehall GL et al (2024) The Europa Thermal Emission Imaging System (E-THEMIS) investigation for the Europa Clipper Mission. Space Sci Rev 220. 10.1007/s11214-024-01074-1 10.1007/s11214-023-01029-y PMC10730683 38130909

[CR57] Christianson K, Jacobel RW, Horgan HJ, Alley RB, Anandakrishnan S, Holland DM, DallaSanta KJ (2016) Basal conditions at the grounding zone of Whillans Ice Stream, West Antarctica, from ice-penetrating radar. J Geophys Res, Earth Surf 121:1954–1983. 10.1002/2015JF003806 10.1002/2015JF003806

[CR58] Chu W, Schroeder DM, Siegfried MR (2018) Retrieval of englacial firn aquifer thickness from ice-penetrating radar sounding in southeastern Greenland. Geophys Res Lett 45:11,770–11,778. 10.1029/2018GL079751 10.1029/2018GL079751

[CR59] Chu W, Hilger AM, Culberg R, Schroeder DM, Jordan TM, Seroussi H, Young DA, Blankenship DD, Vaughan DG (2021) Multisystem synthesis of radar sounding observations of the Amundsen sea sector from the 2004–2005 field season. J Geophys Res, Earth Surf 126:e2021JF006296. 10.1029/2021JF006296 10.1029/2021JF006296 PMC9286636 35865452

[CR60] Chuah TS (1996) Design and development of a coherent radar depth sounder for measurement of Greenland ice sheet thickness. RSL Technical Report 10470-5. University of Kansas

[CR61] Chyba CF (2000) Energy for microbial life on Europa. Nature 403:381–382. 10.1038/35000281 10667778 10.1038/35000281

[CR62] Chyba CF, Ostro SJ, Edwards BC (1998) Radar detectability of a subsurface ocean on Europa. Icarus 134:292–302. 10.1006/icar.1998.5961 10.1006/icar.1998.5961

[CR63] Clark K, Tan-Wang G, Boldt J, Greeley R, Jun I, Lock R, Ludwinski J, Pappalardo R, Van Houten T, Yan T (2009) Return to Europa: overview of the Jupiter Europa orbiter mission. In: 2009 IEEE Aerospace Conference, pp 1–20. 10.1109/AERO.2009.4839315

[CR64] Collins G, Nimmo F (2009) Chaotic terrain on Europa. In: Pappalardo RT, McKinnon WB, Khurana KK (eds) Europa. University of Arizona Press, Tucson, pp 259–281. 10.2307/j.ctt1xp3wdw.17

[CR65] Collins GC, Head III JW, Pappalardo RT, Spaun NA (2000) Evaluation of models for the formation of chaotic terrain on Europa. J Geophys Res, Planets 105:1709–1716. 10.1029/1999JE001143 10.1029/1999JE001143

[CR66] Cooper A, Mclntyre N, Robin GdQ (1982) Driving stresses in the Antarctic ice sheet. Ann Glaciol 3:59–64. 10.3189/S0260305500002536 10.3189/S0260305500002536

[CR67] Cosciotti B, Mattei E, Brin A, Lauro SE, Stillman DE, Cunje A, Hickson D, Caprarelli G, Pettinelli E (2023) Can clay mimic the high reflectivity of briny water below the Martian SPLD? J Geophys Res, Planets 128:e2022JE007513. 10.1029/2022JE007513 10.1029/2022JE007513

[CR68] Costello ES, Phillips CB, Lucey PG, Ghent RR (2021) Impact gardening on Europa and repercussions for possible biosignatures. Nat Astron 5:951–956. 10.1038/s41550-021-01393-1 10.1038/s41550-021-01393-1

[CR69] Cox R, Bauer AW (2015) Impact breaching of Europa’s ice: constraints from numerical modeling. J Geophys Res, Planets 120:1708–1719. 10.1002/2015JE004877 10.1002/2015JE004877

[CR70] Crabtree R, Doake C (1986) Radio-echo investigations of Ronne Ice Shelf. Ann Glaciol 8:37–41. 10.3189/S0260305500001105 10.3189/S0260305500001105

[CR71] Craft KL, Patterson GW, Lowell RP, Germanovich L (2016) Fracturing and flow: investigations on the formation of shallow water sills on Europa. Icarus 274:297–313. 10.1016/j.icarus.2016.01.023 10.1016/j.icarus.2016.01.023

[CR72] Croci R, Seu R, Flamini E, Russo E (2011) The SHAllow RADar (SHARAD) onboard the NASA MRO mission. In: Proc IEEE, vol 99, pp 794–807. 10.1109/JPROC.2010.2104130

[CR73] Culberg R, Schroeder DM (2020) Firn clutter constraints on the design and performance of orbital radar ice sounders. IEEE Trans Geosci Remote Sens 58:6344–6361. 10.1109/TGRS.2020.2976666 10.1109/TGRS.2020.2976666

[CR74] Culberg R, Schroeder DM, Steinbrügge G (2022) Double ridge formation over shallow water sills on Jupiter’s moon Europa. Nat Commun 13:2007. 10.1038/s41467-022-29458-3 35440535 10.1038/s41467-022-29458-3 PMC9018861

[CR75] Culha C, Schroeder DM, Jordan TM, Haynes MS (2020) Assessing the detectability of Europa’s eutectic zone using radar sounding. Icarus 339:113578. 10.1016/j.icarus.2019.113578 10.1016/j.icarus.2019.113578

[CR76] Cumming IG, Wong FH (2005) Digital processing of Synthetic Aperture Radar data. Artech House 1:108–110

[CR77] Daubar IJ, Hayes AG, Collins GC et al. (2024) Planned geological investigations of the Europa Clipper Mission. Space Sci Rev 220:18. 10.1007/s11214-023-01036-z 10.1007/s11214-023-01036-z

[CR78] de Oliveira-Costa A, Tegmark M, Gaensler B, Jonas J, Landecker T, Reich P (2008) A model of diffuse galactic radio emission from 10 MHz to 100 GHz. Mon Not R Astron Soc 388:247–260. 10.1111/j.1365-2966.2008.13376.x 10.1111/j.1365-2966.2008.13376.x

[CR79] Doggett T, Greeley R, Figueredo P, Tanaka K (2009) Geologic stratigraphy and evolution of Europa’s surface. In: Pappalardo RT, McKinnon WB, Khurana KK (eds) Europa. University of Arizona Press, Tucson, pp 137–160. 10.2307/j.ctt1xp3wdw.12

[CR80] Dombard AJ, Patterson GW, Lederer AP, Prockter LM (2013) Flanking fractures and the formation of double ridges on Europa. Icarus 223:74–81. 10.1016/j.icarus.2012.11.021 10.1016/j.icarus.2012.11.021

[CR81] Dowell J, Taylor GB, Schinzel FK, Kassim NE, Stovall K (2017) The LWA1 low frequency sky survey. Mon Not R Astron Soc 469:4537–4550. 10.1093/mnras/stx1136 10.1093/mnras/stx1136

[CR82] Drewry DJ (1983) Antarctica: glaciological and geophysical folio. University of Cambridge, Scott Polar Research Institute

[CR83] Eluszkiewicz J (2004) Dim prospects for radar detection of Europa’s ocean. Icarus 170:234–236. 10.1016/j.icarus.2004.02.011 10.1016/j.icarus.2004.02.011

[CR84] Engelhardt H, Determann J (1987) Borehole evidence for a thick layer of basal ice in the central Ronne Ice Shelf. Nature 327:318–319. 10.1038/327318a0 10.1038/327318a0

[CR85] Evans S (1963) Radio techniques for the measurement of ice thickness. Polar Rec 11:406–410. 10.1017/S0032247400053523 10.1017/S0032247400053523

[CR86] Ewert H, Popov SV, Richter A, Schwabe J, Scheinert M, Dietrich R (2012) Precise analysis of ICESat altimetry data and assessment of the hydrostatic equilibrium for subglacial Lake Vostok, East Antarctica. Geophys J Int 191:557–568. 10.1111/j.1365-246X.2012.05649.x 10.1111/j.1365-246X.2012.05649.x

[CR87] Fagents SA (2003) Considerations for effusive cryovolcanism on Europa: the post-Galileo perspective. J Geophys Res, Planets 108:E12. 10.1029/2003JE002128 10.1029/2003JE002128

[CR88] Fagents SA, Greeley R, Sullivan RJ, Pappalardo RT Prockter LM, The Galileo SSI Team (2000) Cryomagmatic mechanisms for the formation of rhadamanthys linea, triple band margins, and other low-albedo features on Europa. Icarus 144:54–88. 10.1006/icar.1999.6254 10.1006/icar.1999.6254

[CR89] Ferro A (2019) Squinted SAR focusing for improving automatic radar sounder data analysis and enhancement. Int J Remote Sens 40:4762–4786. 10.1080/01431161.2019.1573339 10.1080/01431161.2019.1573339

[CR90] Fischer PD, Brown ME, Hand KP (2015) Spatially resolved spectroscopy of Europa: the distinct spectrum of large-scale chaos. Astron J 150:164. 10.1088/0004-6256/150/5/164 10.1088/0004-6256/150/5/164

[CR91] Franke S, Jansen D, Beyer S, Neckel N, Binder T, Paden J, Eisen O (2021) Complex basal conditions and their influence on ice flow at the onset of the northeast Greenland ice stream. J Geophys Res, Earth Surf 126:e2020JF005689. 10.1029/2020JF005689 10.1029/2020JF005689

[CR92] Frémand AC, Fretwell P, Bodart JA, Pritchard HD, Aitken A, Bamber JL, Bell R, Bianchi C, Bingham RG, Blankenship DD (2023) Antarctic bedmap data: findable, accessible, interoperable, and reusable (FAIR) sharing of 60 years of ice bed, surface, and thickness data. Earth Syst Sci Data 15:2695–2710. 10.5194/essd-15-2695-2023 10.5194/essd-15-2695-2023

[CR93] Fretwell P, Pritchard HD, Vaughan DG, Bamber JL, Barrand NE, Bell R, Bianchi C, Bingham RG, Blankenship DD, Casassa G, Catania G, Callens D, Conway H, Cook AJ, Corr HFJ, Damaske D, Damm V, Ferraccioli F, Forsberg R, Fujita S, Gim Y, Gogineni P, Griggs JA, Hindmarsh RCA, Holmlund P, Holt JW, Jacobel RW, Jenkins A, Jokat W, Jordan T, King EC, Kohler J, Krabill W, Riger-Kusk M, Langley KA, Leitchenkov G, Leuschen C, Luyendyk BP, Matsuoka K, Mouginot J, Nitsche FO, Nogi Y, Nost OA, Popov SV, Rignot E, Rippin DM, Rivera A, Roberts J, Ross N, Siegert MJ, Smith AM, Steinhage D, Studinger M, Sun B, Tinto BK, Welch BC, Wilson D, Young DA, Xiangbin C, Zirizzotti A (2013) Bedmap2: improved ice bed, surface and thickness datasets for Antarctica. Cryosphere 7:375–393. 10.5194/tc-7-375-2013 10.5194/tc-7-375-2013

[CR94] Fricker HA, Padman L (2012) Thirty years of elevation change on Antarctic Peninsula ice shelves. J Geophys Res, Oceans 117:C2. 10.1029/2011JC007126 10.1029/2011JC007126

[CR95] Fricker HA, Popov S, Allison I, Young N (2001) Distribution of marine ice beneath the Amery Ice Shelf. Geophys Res Lett 28:2241–2244. 10.1029/2000GL012461 10.1029/2000GL012461

[CR96] Fujita S, Matsuoka T, Ishida T, Matsuoka K, Mae S (2000) A summary of the complex dielectric permittivity of ice in the megahertz range and its applications for radar sounding of polar ice sheets. Hokkaido University Press, pp 185–212

[CR97] Fung AK, Li Z, Chen KS (1992) Backscattering from a randomly rough dielectric surface. IEEE Trans Geosci Remote Sens 30:356–369. 10.1109/36.134085 10.1109/36.134085

[CR98] Gaidos EJ, Nimmo F (2000) Tectonics and water on Europa. Nature 405:637–637. 10.1038/35015170 10864313 10.1038/35015170

[CR99] Gassot O, Herique A, Kofman W, Cecconi B, Witasse O (2022) Performances of the passive SAR imaging of Jupiter’s Icy Moons. IEEE Trans Geosci Remote Sens 60:1–13. 10.1109/TGRS.2022.3172633 10.1109/TGRS.2022.3172633

[CR100] Gerekos C, Bruzzone L, Imai M (2020) A coherent method for simulating active and passive radar sounding of the Jovian Icy Moons. IEEE Trans Geosci Remote Sens 58:2250–2265. 10.1109/TGRS.2019.2945079 10.1109/TGRS.2019.2945079

[CR101] Gerekos C, Steinbrügge G, Jebaraj IC, Casillas A, Donini E, Sánchez-Cano B, Lester M, Magdalenić J, Peters ST, Romero-Wolf AF, Blankenship DD (2024) Observation of solar radio burst events from Mars orbit with the Shallow Radar instrument. Astron Astrophys 683:A56. 10.1051/0004-6361/202347900 10.1051/0004-6361/202347900

[CR102] Gogineni S, Chuah T, Allen C, Jezek K, Moore RK (1998) An improved coherent radar depth sounder. J Glaciol 44:659–669. 10.3189/S0022143000002161 10.3189/S0022143000002161

[CR103] Goldsby DL, Kohlstedt DL (2001) Superplastic deformation of ice: experimental observations. J Geophys Res, Solid Earth 106:11017–11030. 10.1029/2000JB900336 10.1029/2000JB900336

[CR104] Grasset O, Dougherty M, Coustenis A, Bunce E, Erd C, Titov D, Blanc M, Coates A, Drossart P, Fletcher L (2013) JUpiter ICy moons Explorer (JUICE): an ESA mission to orbit Ganymede and to characterise the Jupiter system. Planet Space Sci 78:1–21. 10.1016/j.pss.2012.12.002 10.1016/j.pss.2012.12.002

[CR105] Greely R, Johnson T (2004) The jupitor icy moons orbiter project: the scientific rationale. Eos Trans AGU 85:337–343. 10.1029/2004EO360001 10.1029/2004EO360001

[CR106] Greenberg R, Hoppa GV, Tufts BR, Geissler P, Riley J, Kadel S (1999) Chaos on Europa. Icarus 141:263–286. 10.1006/icar.1999.6187 10.1006/icar.1999.6187

[CR107] Grima C, Blankenship DD, Young DA, Schroeder DM (2014a) Surface slope control on firn density at Thwaites Glacier, West Antarctica: results from airborne radar sounding. Geophys Res Lett 41:6787–6794. 10.1002/2014GL061635 10.1002/2014GL061635

[CR108] Grima C, Schroeder DM, Blankenship DD, Young DA (2014b) Planetary landing-zone reconnaissance using ice-penetrating radar data: concept validation in Antarctica. Planet Space Sci 103:191–204. 10.1016/j.pss.2014.07.018 10.1016/j.pss.2014.07.018

[CR109] Grima C, Blankenship DD, Schroeder DM (2015) Radar signal propagation through the ionosphere of Europa. Planet Space Sci 117:421–428. 10.1016/j.pss.2015.08.017 10.1016/j.pss.2015.08.017

[CR110] Grima C, Greenbaum JS, Lopez Garcia EJ, Soderlund KM, Rosales A, Blankenship DD, Young DA (2016) Radar detection of the brine extent at McMurdo Ice Shelf, Antarctica, and its control by snow accumulation. Geophys Res Lett 43:7011–7018. 10.1002/2016GL069524 10.1002/2016GL069524

[CR111] Grima C, Mastrogiuseppe M, Hayes AG, Wall SD, Lorenz RD, Hofgartner JD, Stiles B, Elachi C, Cassini RADAR Team (2017) Surface roughness of Titan’s hydrocarbon seas. Earth Planet Sci Lett 474:20–24. 10.1016/j.epsl.2017.06.007 10.1016/j.epsl.2017.06.007

[CR112] Grima C, Koch I, Greenbaum J, Soderlund K, Blankenship D, Young D, Schroeder D, Fitzsimons S (2019) Surface and basal boundary conditions at the southern McMurdo and Ross Ice Shelves, Antarctica. J Glaciol 65:675–688. 10.1017/jog.2019.44 10.1017/jog.2019.44

[CR113] Grima C, Mouginot J, Kofman W, Hérique A, Beck P (2022a) The basal detectability of an ice-covered Mars by MARSIS. Geophys Res Lett 49:e2021GL096518. 10.1029/2021GL096518 10.1029/2021GL096518

[CR114] Grima C, Putzig NE, Campbell BA, Perry M, Gulick SPS, Miller RC, Russell AT, Scanlan KM, Steinbrügge G, Young DA, Kempf SD, Ng G, Buhl D, Blankenship DD (2022b) Investigating the Martian surface at decametric scale: population, distribution, and dimension of heterogeneity from radar statistics. Planet Sci J 3:236. 10.3847/PSJ/ac9277 10.3847/PSJ/ac9277

[CR115] Grimm R, Stillman D (2019) On the electrical properties of meridianiite and implications for radar sounding of icy satellites. Earth Planet Sci Lett 520:34–39. 10.1016/j.epsl.2019.05.010 10.1016/j.epsl.2019.05.010

[CR116] Grimm RE, Stillman DE, Dec SF, Bullock MA (2008) Low-frequency electrical properties of polycrystalline saline ice and salt hydrates. J Phys Chem B 112:15382–15390. 10.1021/jp8055366 19006262 10.1021/jp8055366

[CR117] Gross GW, Wong PM, Humes K (1977) Concentration dependent solute redistribution at the ice–water phase boundary. III. Spontaneous convection. Chloride solutions. J Chem Phys 67:5264–5274. 10.1063/1.434704 10.1063/1.434704

[CR118] Gudmandsen P (1969) Airborne radio echo sounding of the Greenland Ice Sheet. Geogr J 135:548–551. 10.2307/1795099 10.2307/1795099

[CR119] Gurnett DA, Kurth WS, Roux A, Bolton SJ, Thomsen EA, Groene JB (1998) Galileo plasma wave observations near Europa. Geophys Res Lett 25:237–240. 10.1029/97GL03706 10.1029/97GL03706

[CR120] Hammond N (2020) Estimating the Magnitude of Cyclic Slip on Strike-Slip faults on Europa. J Geophys Res, Planets 125:7. 10.1029/2019JE006170 10.1029/2019JE006170

[CR121] Hand KP, Carlson RW, Chyba CF (2007) Energy, chemical disequilibrium, and geological constraints on Europa. Astrobiology 7:1006–1022. 10.1089/ast.2007.0156 18163875 10.1089/ast.2007.0156

[CR122] Hand KP, Chyba CF, Priscu JC, Carlson RW, Nealson KH (2009) Astrobiology and the potential for life on Europa. In: Pappalardo RT, McKinnon WB, Khurana KK (eds) Europa. University of Arizona Press, Tucson, pp 589–630. 10.2307/j.ctt1xp3wdw.32

[CR123] Haynes MS, Chapin E, Moussessian A, Madsen SN (2018a) Surface clutter discrimination analysis for radar sounding interferometry. IEEE Trans Aerosp Electron Syst 55:989–1003. 10.1109/TAES.2018.2867689 10.1109/TAES.2018.2867689

[CR124] Haynes MS, Chapin E, Schroeder DM (2018b) Geometric power fall-off in radar sounding. IEEE Trans Geosci Remote Sens 56:6571–6585. 10.1109/TGRS.2018.2840511 10.1109/TGRS.2018.2840511

[CR125] Head JW, Pappalardo RT (1999) Brine mobilization during lithospheric heating on Europa: implications for formation of chaos terrain, lenticula texture, and color variations. J Geophys Res, Planets 104:27143–27155. 10.1029/1999JE001062 10.1029/1999JE001062

[CR126] Head JW, Pappalardo RT, Sullivan R (1999) Europa: morphological characteristics of ridges and triple bands from Galileo data (E4 and E6) and assessment of a linear diapirism model. J Geophys Res, Planets 104:24223–24236. 10.1029/1998JE001011 10.1029/1998JE001011

[CR127] Heister A, Scheiber R (2018) Coherent large beamwidth processing of radio-echo sounding data. Cryosphere 12:2969–2979. 10.5194/tc-12-2969-2018 10.5194/tc-12-2969-2018

[CR128] Hills BH, Christianson K, Holschuh N (2020) A framework for attenuation method selection evaluated with ice-penetrating radar data at South Pole Lake. Ann Glaciol 61:176–187. 10.1017/aog.2020.32 10.1017/aog.2020.32

[CR129] Holt JW, Safaeinili A, Plaut JJ, Head JW, Phillips RJ, Seu R, Kempf SD, Choudhary P, Young DA, Putzig NE, Biccari D, Gim Y (2008) Radar sounding evidence for buried glaciers in the southern mid-latitudes of Mars. Science 322:1235–1238. 10.1126/science.1164246 19023078 10.1126/science.1164246

[CR130] Horgan HJ, Anandakrishnan S, Jacobel RW, Christianson K, Alley RB, Heeszel DS, Picotti S, Walter JI (2012) Subglacial lake Whillans — seismic observations of a shallow active reservoir beneath a West Antarctic ice stream. Earth Planet Sci Lett 331–332:201–209. 10.1016/j.epsl.2012.02.023 10.1016/j.epsl.2012.02.023

[CR131] Howell SM (2021) The likely thickness of Europa’s icy shell. Planet Sci J 2:129. 10.3847/PSJ/abfe10 10.3847/PSJ/abfe10

[CR132] Hughes JS, Crichton D, Hardman S, Law E, Joyner R, Ramirez P (2014) PDS4: a model-driven planetary science data architecture for long-term preservation. In: 2014 IEEE 30th international conference on data engineering workshops, pp 134–141. 10.1109/ICDEW.2014.6818317

[CR133] Humbert A, Steinhage D, Helm V, Beyer S, Kleiner T (2018) Missing evidence of widespread subglacial lakes at recovery glacier, Antarctica. J Geophys Res, Earth Surf 123:2802–2826. 10.1029/2017JF004591 10.1029/2017JF004591

[CR134] Humphrey NF, Harper JT, Pfeffer WT (2012) Thermal tracking of meltwater retention in Greenland’s accumulation area. J Geophys Res, Earth Surf 117:F1. 10.1029/2011JF002083 10.1029/2011JF002083

[CR135] Hussmann H, Shoji D, Steinbrügge G, Stark A, Sohl F (2016) Constraints on dissipation in the deep interiors of Ganymede and Europa from tidal phase-lags. Celest Mech Dyn Astron 126:131–144. 10.1007/s10569-016-9721-0 10.1007/s10569-016-9721-0

[CR136] Ilisei A-M, Khodadadzadeh M, Ferro A, Bruzzone L (2019) An automatic method for subglacial lake detection in ice sheet radar sounder data. IEEE Trans Geosci Remote Sens 57:3252–3270. 10.1109/TGRS.2018.2882911 10.1109/TGRS.2018.2882911

[CR137] Jebaraj IC, Magdalenić J, Podladchikova T, Scolini C, Pomoell J, Veronig AM, Dissauer K, Krupar V, Kilpua EKJ, Poedts S (2020) Using radio triangulation to understand the origin of two subsequent type II radio bursts. Astron Astrophys 639:A56. 10.1051/0004-6361/201937273 10.1051/0004-6361/201937273

[CR138] Jenkins A, Doake C (1991) Ice-ocean interaction on Ronne Ice Shelf, Antarctica. J Geophys Res, Oceans 96:791–813. 10.1029/90JC01952 10.1029/90JC01952

[CR139] Jezek KC (1984) A modified theory of bottom crevasses used as a means for measuring the buttressing effect of ice shelves on inland ice sheets. J Geophys Res, Solid Earth 89:1925–1931. 10.1029/JB089iB03p01925 10.1029/JB089iB03p01925

[CR140] Jezek KC, Bentley CR (1983) Field studies of bottom crevasses in the Ross Ice Shelf, Antarctica. J Glaciol 29:118–126. 10.3189/S0022143000005189 10.3189/S0022143000005189

[CR141] Jezek KC, Bentley CR, Clough JW (1979) Electromagnetic sounding of bottom crevasses on the Ross Ice Shelf, Antarctica. J Glaciol 24:321–330. 10.3189/S0022143000014842 10.3189/S0022143000014842

[CR142] Jia X, Kivelson MG, Khurana KK, Kurth WS (2018) Evidence of a plume on Europa from Galileo magnetic and plasma wave signatures. Nat Astron 2:459–464. 10.1038/s41550-018-0450-z 10.1038/s41550-018-0450-z

[CR143] Johnston SA, Montési LG (2014) Formation of ridges on Europa above crystallizing water bodies inside the ice shell. Icarus 237:190–201. 10.1016/j.icarus.2014.04.026 10.1016/j.icarus.2014.04.026

[CR144] Jordan R, Picardi G, Plaut J, Wheeler K, Kirchner D, Safaeinili A, Johnson W, Seu R, Calabrese D, Zampolini E, Cicchetti A, Huff R, Gurnett D, Ivanov A, Kofman W, Orosei R, Thompson T, Edenhofer P, Bombaci O (2009) The Mars Express MARSIS sounder instrument. Planet Space Sci 57:1975–1986. 10.1016/j.pss.2009.09.016 10.1016/j.pss.2009.09.016

[CR145] Jordan TM, Cooper MA, Schroeder DM, Williams CN, Paden JD, Siegert MJ, Bamber JL (2017) Self-affine subglacial roughness: consequences for radar scattering and basal water discrimination in northern Greenland. Cryosphere 11:1247–1264. 10.5194/tc-11-1247-2017 10.5194/tc-11-1247-2017

[CR146] Jordan TM, Williams CN, Schroeder DM, Martos YM, Cooper MA, Siegert MJ, Paden JD, Huybrechts P, Bamber JL (2018) A constraint upon the basal water distribution and thermal state of the Greenland Ice Sheet from radar bed echoes. Cryosphere 12:2831–2854. 10.5194/tc-12-2831-2018 10.5194/tc-12-2831-2018

[CR147] Joughin I, Vaughan DG (2004) Marine ice beneath the Filchner–Ronne Ice Shelf, Antarctica: a comparison of estimated thickness distributions. Ann Glaciol 39:511–517. 10.3189/172756404781814717 10.3189/172756404781814717

[CR148] Kalousová K, Souček O, Tobie G, Choblet G, Čadek O (2016) Water generation and transport below Europa’s strike-slip faults. J Geophys Res, Planets 121:2444–2462. 10.1002/2016JE005188 10.1002/2016JE005188

[CR149] Kalousová K, Schroeder DM, Soderlund KM (2017) Radar attenuation in Europa’s ice shell: obstacles and opportunities for constraining the shell thickness and its thermal structure. J Geophys Res, Planets 122:524–545. 10.1002/2016JE005110 10.1002/2016JE005110

[CR150] Kapitsa A, Ridley J, Robin GdQ, Siegert M, Zotikov I (1996) A large deep freshwater lake beneath the ice of central East Antarctica. Nature 381:684–686. 10.1038/381684a0 10.1038/381684a0

[CR151] Kargel JS (1991) Brine volcanism and the interior structures of asteroids and icy satellites. Icarus 94:368–390. 10.1016/0019-1035(91)90235-L 10.1016/0019-1035(91)90235-L

[CR152] Kattenhorn SA, Hurford T (2009) Tectonics of Europa. In: Pappalardo RT, McKinnon WB, Khurana KK (eds) Europa. University of Arizona Press, Tucson, pp 199–236. 10.2307/j.ctt1xp3wdw.15

[CR153] Khurana K, Kivelson M, Stevenson D, Schubert G, Russell C, Walker R, Polanskey C (1998) Induced magnetic fields as evidence for subsurface oceans in Europa and Callisto. Nature 395:777–780. 10.1038/27394 9796812 10.1038/27394

[CR154] Kim W, Chinn JZ, Jun I, Garrett HB (2019) Approach for Defining Internal Electrostatic Discharge Design Environment of a Jovian Mission. In: 2019 19th European Conference on Radiation and Its Effects on Components and Systems (RADECS). pp 1–3. 10.1109/RADECS47380.2019.9745730

[CR155] Kivelson MG, Khurana KK, Russell CT, Volwerk M, Walker RJ, Zimmer C (2000) Galileo magnetometer measurements: a stronger case for a subsurface ocean at Europa. Science 289:1340–1343. 10.1126/science.289.5483.1340 10958778 10.1126/science.289.5483.1340

[CR156] Kliore AJ, Hinson DP, Flasar FM, Nagy AF, Cravens TE (1997) The ionosphere of Europa from Galileo radio occultations. Science 277:355–358. 10.1126/science.277.5324.355 9219689 10.1126/science.277.5324.355

[CR157] Kobayashi T, Kim J-H, Lee SR, Kumamoto A, Nakagawa H, Oshigami S, Oya H, Yamaguchi Y, Yamaji A, Ono T (2011) Synthetic Aperture Radar processing of Kaguya lunar radar sounder data for lunar subsurface imaging. IEEE Trans Geosci Remote Sens 50:2161–2174. 10.1109/TGRS.2011.2171349 10.1109/TGRS.2011.2171349

[CR158] Kofman W, Orosei R, Pettinelli E (2010) Radar signal propagation and detection through ice. Space Sci Rev 153:249–271. 10.1007/s11214-010-9642-2 10.1007/s11214-010-9642-2

[CR159] Kurth W, Gurnett D, Persoon A, Roux A, Bolton S, Alexander C (2001) The plasma wave environment of Europa. Planet Space Sci 49:345–363. 10.1016/S0032-0633(00)00156-2 10.1016/S0032-0633(00)00156-2

[CR160] Kwok R, Johnson WT (1989) Block adaptive quantization of Magellan SAR data. IEEE Trans Geosci Remote Sens 27:375–383. 10.1109/36.29557 10.1109/36.29557

[CR161] Lalich D, Hayes A, Poggiali V (2022) Explaining bright radar reflections below the south pole of Mars without liquid water. Nat Astron 6:1142–1146. 10.1038/s41550-022-01775-z 10.1038/s41550-022-01775-z

[CR162] Lambrecht A, Sandhäger H, Vaughan DG, Mayer C (2007) New ice thickness maps of Filchner–Ronne Ice Shelf, Antarctica, with specific focus on grounding lines and marine ice. Antarct Sci 19:521–532. 10.1017/S0954102007000661 10.1017/S0954102007000661

[CR163] Lauro SE, Pettinelli E, Caprarelli G, Guallini L, Rossi AP, Mattei E, Cosciotti B, Cicchetti A, Soldovieri F, Cartacci M, Di Paolo F, Noschese R, Orosei R (2021) Multiple subglacial water bodies below the south pole of Mars unveiled by new MARSIS data. Nat Astron 5:63–70. 10.1038/s41550-020-1200-6 10.1038/s41550-020-1200-6

[CR164] Lauro SE, Pettinelli E, Caprarelli G, Baniamerian J, Mattei E, Cosciotti B, Stillman DE, Primm KM, Soldovieri F, Orosei R (2022) Using MARSIS signal attenuation to assess the presence of south polar layered deposit subglacial brines. Nat Commun 13:5686. 10.1038/s41467-022-33389-4 36171186 10.1038/s41467-022-33389-4 PMC9519933

[CR165] Lauro SE, Pettinelli E, Caprarelli G, Guallini L, Rossi AP, Mattei E, Cosciotti B, Cicchetti A, Soldovieri F, Cartacci M (2023) Reply to: Explaining bright radar reflections below the south pole of Mars without liquid water. Nat Astron 7:259–261. 10.1038/s41550-022-01871-0 10.1038/s41550-022-01871-0

[CR166] Legarsky JJ, Gogineni SP, Akins TL (2001) Focused Synthetic Aperture Radar processing of ice-sounder data collected over the Greenland ice sheet. IEEE Trans Geosci Remote Sens 39:2109–2117. 10.1109/36.957274 10.1109/36.957274

[CR167] Lesage E, Massol H, Howell SM, Schmidt F (2022) Simulation of freezing cryomagma reservoirs in viscoelastic ice shells. Planet Sci J 3:170. 10.3847/PSJ/ac75bf 10.3847/PSJ/ac75bf

[CR168] Lewis K, Klaasen K, Susca S, Oaida B, Larson M, Vanelli T, Murray A, Jones L, Thomas V, Frank L (2016) Use of model payload for Europa mission development IEEE Aerospace Conference. In: 2016 IEEE Aerospace Conference, pp 1–14. 10.1109/AERO.2016.7500708

[CR169] Ligier N, Poulet F, Carter J, Brunetto R, Gourgeot F (2016) VLT/SINFONI observations of Europa: new insights into the surface composition. Astron J 151:163. 10.3847/0004-6256/151/6/163 10.3847/0004-6256/151/6/163

[CR170] Lindzey LE (2015) In: Brief introd. Ice-penetrating radar. https://lindzey.github.io/blog/2015/07/27/a-brief-introduction-to-ice-penetrating-radar/

[CR171] Lindzey LE, Beem LH, Young DA, Quartini E, Blankenship DD, Lee C-K, Lee WS, Lee JI, Lee J (2020) Aerogeophysical characterization of an active subglacial lake system in the David Glacier catchment, Antarctica. Cryosphere 14:2217–2233. 10.5194/tc-14-2217-2020 10.5194/tc-14-2217-2020

[CR172] Livingstone SJ, Li Y, Rutishauser A, Sanderson RJ, Winter K, Mikucki JA, Björnsson H, Bowling JS, Chu W, Dow CF, Fricker HA, McMillan M, Ng FSL, Ross N, Siegert MJ, Siegfried M, Sole AJ (2022) Subglacial lakes and their changing role in a warming climate. Nat Rev Earth Environ 3:106–124. 10.1038/s43017-021-00246-9 10.1038/s43017-021-00246-9

[CR173] Ludwinski J, Guman M, Johannesen J, Mitchell R, Staehle R (1998) The Europa Orbiter Mission Design

[CR174] Lythe MB, Vaughan DG (2001) BEDMAP: a new ice thickness and subglacial topographic model of Antarctica. J Geophys Res, Solid Earth 106:11335–11351. 10.1029/2000JB900449 10.1029/2000JB900449

[CR175] MacGregor JA, Winebrenner DP, Conway H, Matsuoka K, Mayewski PA, Clow GD (2007) Modeling englacial radar attenuation at Siple Dome, West Antarctica, using ice chemistry and temperature data. J Geophys Res, Earth Surf 112:F3. 10.1029/2006JF000717 10.1029/2006JF000717

[CR176] MacGregor JA, Li J, Paden JD, Catania GA, Clow GD, Fahnestock MA, Gogineni SP, Grimm RE, Morlighem M, Nandi S, Seroussi H, Stillman DE (2015) Radar attenuation and temperature within the Greenland Ice Sheet. J Geophys Res, Earth Surf 120:983–1008. 10.1002/2014JF003418 10.1002/2014JF003418

[CR177] Machguth H, MacFerrin M, van As D, Box JE, Charalampidis C, Colgan W, Fausto RS, Meijer HA, Mosley-Thompson E, van de Wal RS (2016) Greenland meltwater storage in firn limited by near-surface ice formation. Nat Clim Change 6:390–393. 10.1038/nclimate2899 10.1038/nclimate2899

[CR178] Manga M, Michaut C (2017) Formation of lenticulae on Europa by saucer-shaped sills. Icarus 286:261–269. 10.1016/j.icarus.2016.10.009 10.1016/j.icarus.2016.10.009

[CR179] Matsuoka K (2011) Pitfalls in radar diagnosis of ice-sheet bed conditions: Lessons from englacial attenuation models. Geophys Res Lett 38:L05505. 10.1029/2010GL046205 10.1029/2010GL046205

[CR180] Matsuoka T, Fujita S, Mae S (1996) Effect of temperature on dielectric properties of ice in the range 5–39 GHz. J Appl Phys 80:5884–5890. 10.1063/1.363582 10.1063/1.363582

[CR181] Matsuoka K, MacGregor JA, Pattyn F (2012) Predicting radar attenuation within the Antarctic ice sheet. Earth Planet Sci Lett 359:173–183. 10.1016/j.epsl.2012.10.018 10.1016/j.epsl.2012.10.018

[CR182] Mattei E, Pettinelli E, Lauro SE, Stillman DE, Cosciotti B, Marinangeli L, Tangari AC, Soldovieri F, Orosei R, Caprarelli G (2022) Assessing the role of clay and salts on the origin of MARSIS basal bright reflections. Earth Planet Sci Lett 579:117370. 10.1016/j.epsl.2022.117370 10.1016/j.epsl.2022.117370

[CR183] Mazarico E, Barker MK, Neumann GA, Zuber MT, Smith DE (2014) Detection of the lunar body tide by the Lunar Orbiter Laser Altimeter. Geophys Res Lett 41:2282–2288. 10.1002/2013GL059085 26074646 10.1002/2013GL059085 PMC4459177

[CR184] Mazarico E, Buccino D, Castillo-Rogez J et al. (2023) The Europa Clipper gravity and radio science investigation. Space Sci Rev 219:30. 10.1007/s11214-023-00972-0 10.1007/s11214-023-00972-0

[CR185] McCarthy C, Cooper RF, Goldsby DL, Durham WB, Kirby SH (2011) Transient and steady state creep response of ice I and magnesium sulfate hydrate eutectic aggregates. J Geophys Res, Planets 116:E04007. 10.1029/2010JE003689 10.1029/2010JE003689

[CR186] McCollom TM (1999) Methanogenesis as a potential source of chemical energy for primary biomass production by autotrophic organisms in hydrothermal systems on Europa. J Geophys Res, Planets 104:30729–30742. 10.1029/1999JE001126 10.1029/1999JE001126

[CR187] McGrath M, Hansen C, Hendrix A (2009) Observations of Europa’s tenuous atmosphere. In: Pappalardo RT, McKinnon WB, Khurana KK (eds) Europa. University of Arizona Press, Tucson, pp 485–506. 10.2307/j.ctt1xp3wdw.26

[CR188] McKinnon WB (1999) Convective instability in Europa’s floating ice shell. Geophys Res Lett 26:951–954. 10.1029/1999GL900125 10.1029/1999GL900125

[CR189] McKinnon W (2005) Radar sounding of convecting ice shells in the presence of convection: application to Europa, Ganymede, and Callisto. In: Workshop on Radar Investigations of Planetary and Terrestrial Environments, pp 6039

[CR190] Melosh HJ, Ekholm AG, Showman AP, Lorenz RD (2004) The temperature of Europa’s subsurface water ocean. Icarus 168:498–502. 10.1016/j.icarus.2003.11.026 10.1016/j.icarus.2003.11.026

[CR191] Mercer JH (1978) West Antarctic ice sheet and CO2 greenhouse effect: a threat of disaster. Nature 271:321–325. 10.1038/271321a0 10.1038/271321a0

[CR192] Michaut C, Manga M (2014) Domes, pits, and small chaos on Europa produced by water sills. J Geophys Res, Planets 119:550–573. 10.1002/2013JE004558 10.1002/2013JE004558

[CR193] Mitri G, Showman AP (2005) Convective–conductive transitions and sensitivity of a convecting ice shell to perturbations in heat flux and tidal-heating rate: implications for Europa. Icarus 177:447–460. 10.1016/j.icarus.2005.03.019 10.1016/j.icarus.2005.03.019

[CR194] Molaro JL, Choukroun M, Phillips CB, Phelps ES, Hodyss R, Mitchell KL, Lora JM, Meirion-Griffith G (2019) The microstructural evolution of water ice in the Solar System through sintering. J Geophys Res, Planets 124:243–277. 10.1029/2018JE005773 32874819 10.1029/2018JE005773 PMC7458059

[CR195] Moore JC (2000) Models of radar absorption in europan ice. Icarus 147:292–300. 10.1006/icar.2000.6425 10.1006/icar.2000.6425

[CR196] Moore WB, Schubert G (2000) The tidal response of Europa. Icarus 147:317–319. 10.1006/icar.2000.6460 10.1006/icar.2000.6460

[CR197] Moore JC, Reid AP, Kipfstuhl J (1994) Microstructure and electrical properties of marine ice and its relationship to meteoric ice and sea ice. J Geophys Res, Oceans 99:5171–5180. 10.1029/93JC02832 10.1029/93JC02832

[CR198] Moore JM, Asphaug E, Belton MJ, Bierhaus B, Breneman HH, Brooks SM, Chapman CR, Chuang FC, Collins GC, Giese B (2001) Impact features on Europa: results of the Galileo Europa Mission (GEM). Icarus 151:93–111. 10.1006/icar.2000.6558 10.1006/icar.2000.6558

[CR199] Moore JM, Black G, Buratti B, Phillips CB, Spencer J, Sullivan R (2009) Surface properties, regolith, and landscape degradation. In: Pappalardo RT, McKinnon WB, Khurana KK (eds) Europa. University of Arizona Press, Tucson, pp 329–349. 10.2307/j.ctt1xp3wdw.19

[CR200] Mortimer CA, Sharp M, Wouters B (2016) Glacier surface temperatures in the Canadian High Arctic, 2000–15. J Glaciol 62:963–975. 10.1017/jog.2016.80 10.1017/jog.2016.80

[CR201] Mouginot J, Kofman W, Safaeinili A, Hérique A (2008) Correction of the ionospheric distortion on the MARSIS surface sounding echoes. Planet Space Sci 56:917–926. 10.1016/j.pss.2008.01.010 10.1016/j.pss.2008.01.010

[CR202] Mouginot J, Kofman W, Safaeinili A, Grima C, Herique A, Plaut JJ (2009) MARSIS surface reflectivity of the south residual cap of Mars. Icarus 201:454–459. 10.1016/j.icarus.2009.01.009 10.1016/j.icarus.2009.01.009

[CR203] Moussessian A, Jordan RL, Rodriguez E, Safaeinili A, Akins TL, Edelstein WN, Kim Y, Gogineni SP (2000) A new coherent radar for ice sounding in Greenland. In: IGARSS 2000. IEEE 2000 international geoscience and remote sensing symposium. Taking the pulse of the planet: the role of remote sensing in managing the environment. Proceedings (Cat. No. 00CH37120), vol 2, pp 484–486. 10.1109/IGARSS.2000.861604

[CR204] National Research Council (2003) New frontiers in the Solar System: an integrated exploration strategy. The National Academies Press, Washington DC. 10.17226/10432

[CR205] National Research Council (2011) Vision and voyages for planetary science in the decade 2013-2022. The National Academies Press, Washington DC. 10.17226/13117

[CR206] Nimmo F, Gaidos E (2002) Strike-slip motion and double ridge formation on Europa. J Geophys Res, Planets 107:5-1. 10.1029/2000JE001476 10.1029/2000JE001476

[CR207] Nimmo F, Giese B (2005) Thermal and topographic tests of Europa chaos formation models from Galileo E15 observations. Icarus 177:327–340. 10.1016/j.icarus.2004.10.034 10.1016/j.icarus.2004.10.034

[CR208] Nimmo F, Manga M (2002) Causes, characteristics and consequences of convective diapirism on Europa. Geophys Res Lett 29:24-1–24-4. 10.1029/2002GL015754 10.1029/2002GL015754

[CR209] Nimmo F, Manga M (2009) Geodynamics of Europa’s icy shell. In: Pappalardo RT, McKinnon WB, Khurana KK (eds) Europa. University of Arizona Press, Tucson, pp 381–404. 10.2307/j.ctt1xp3wdw.22

[CR210] Nimmo F, Schenk PM (2008) Stereo and Photoclinometric Comparisons and Topographic Roughness of Europa. In: 39th Lunar and Planetary Science Conference, LPI Contribution No. 1391, pp 1464

[CR211] Nimmo F, Pappalardo RT, Giese B (2003) On the origins of band topography, Europa. Icarus 166:21–32. 10.1016/j.icarus.2003.08.002 10.1016/j.icarus.2003.08.002

[CR212] Oerter H, Kipfstuhl J, Determann J, Miller H, Wagenbach D, Minikin A, Graft W (1992) Evidence for basal marine ice in the Filchner–Ronne Ice Shelf. Nature 358:399–401. 10.1038/358399a0 10.1038/358399a0

[CR213] Ono T, Kumamoto A, Kasahara Y, Yamaguchi Y, Yamaji A, Kobayashi T, Oshigami S, Nakagawa H, Goto Y, Hashimoto K, Omura Y, Imachi T, Matsumoto H, Oya H (2010) The Lunar Radar Sounder (LRS) onboard the KAGUYA (SELENE) spacecraft. Space Sci Rev 154:145–192. 10.1007/s11214-010-9673-8 10.1007/s11214-010-9673-8

[CR214] Onoda GY, Liniger EG (1990) Random loose packings of uniform spheres and the dilatancy onset. Phys Rev Lett 64:2727. 10.1103/PhysRevLett.64.2727 10041794 10.1103/PhysRevLett.64.2727

[CR215] Orosei R, Jordan RL, Morgan DD, Cartacci M, Cicchetti A, Duru F, Gurnett DA, Heggy E, Kirchner DL, Noschese R, Kofman W, Masdea A, Plaut JJ, Seu R, Watters TR, Picardi G (2015) Mars Advanced Radar for Subsurface and Ionospheric Sounding (MARSIS) after nine years of operation: a summary. Planet Space Sci 112:98–114. 10.1016/j.pss.2014.07.010 10.1016/j.pss.2014.07.010

[CR216] Orosei R, Lauro SE, Pettinelli E, Cicchetti A, Coradini M, Cosciotti B, Di Paolo F, Flamini E, Mattei E, Pajola M (2018) Radar evidence of subglacial liquid water on Mars. Science 361:490–493. 10.1126/science.aar7268 30045881 10.1126/science.aar7268

[CR217] Orosei R, Caprarelli G, Lauro S, Pettinelli E, Cartacci M, Cicchetti A, Cosciotti B, De Lorenzis A, De Nunzio G, Mattei E (2022) Numerical simulations of radar echoes rule out basal CO2 ice deposits at Ultimi Scopuli, Mars. Icarus 386:115163. 10.1016/j.icarus.2022.115163 10.1016/j.icarus.2022.115163

[CR218] Pappalardo RT, Barr AC (2004) The origin of domes on Europa: the role of thermally induced compositional diapirism. Geophys Res Lett 31:L01701. 10.1029/2003GL019202 10.1029/2003GL019202

[CR219] Pappalardo RT, Head JW, Greeley R, Sullivan RJ, Pilcher C, Schubert G, Moore WB, Carr MH, Moore JM, Belton MJS, Goldsby DL (1998) Geological evidence for solid-state convection in Europa’s ice shell. Nature 391:365–368. 10.1038/34862 9450750 10.1038/34862

[CR220] Pappalardo RT, Belton MJ, Breneman H, Carr M, Chapman CR, Collins G, Denk T, Fagents S, Geissler PE, Giese B (1999) Does Europa have a subsurface ocean? Evaluation of the geological evidence. J Geophys Res, Planets 104:24015–24055. 10.1029/1998JE000628 10.1029/1998JE000628

[CR221] Pappalardo R, Vance S, Bagenal F, Bills B, Blaney D, Blankenship D, Brinckerhoff W, Connerney J, Hand K, Hoehler TM (2013) Science potential from a Europa lander. Astrobiology 13:740–773. 10.1089/ast.2013.1003 23924246 10.1089/ast.2013.1003

[CR222] Pappalardo RT, Buratti BJ, Korth H et al (2024) Science overview of the Europa Clipper Mission. Space Sci Rev 220

[CR223] Peeples WJ, Sill WR, May TW, Ward SH, Phillips RJ, Jordan RL, Abbott EA, Killpack TJ (1978) Orbital radar evidence for lunar subsurface layering in Maria Serenitatis and crisium. J Geophys Res, Solid Earth 83:3459–3468. 10.1029/JB083iB07p03459 10.1029/JB083iB07p03459

[CR224] Peters M, Blankenship D, Holt J, Morse D, Young D (2005a) Airborne radar sounding: a survey of the Thwaites Glacier Catchment of the Amundsen Sea Embayment, West Antarctica. In: AGU Fall Meeting, pp U23C-06

[CR225] Peters ME, Blankenship DD, Morse DL (2005b) Analysis techniques for coherent airborne radar sounding: Application to West Antarctic ice streams. J Geophys Res, Solid Earth 110:B06303. 10.1029/2004JB003222 10.1029/2004JB003222

[CR226] Peters ME, Blankenship DD, Carter SP, Kempf SD, Young DA, Holt JW (2007a) Along-track focusing of airborne radar sounding data from West Antarctica for improving basal reflection analysis and layer detection. IEEE Trans Geosci Remote Sens 45:2725–2736. 10.1109/TGRS.2007.897416 10.1109/TGRS.2007.897416

[CR227] Peters ME, Blankenship DD, Smith DE, Holt JW, Kempf SD (2007b) The distribution and classification of bottom crevasses from radar sounding of a large tabular iceberg. IEEE Geosci Remote Sens Lett 4:142–146. 10.1109/LGRS.2006.887057 10.1109/LGRS.2006.887057

[CR228] Peters LE, Anandakrishnan S, Holland CW, Horgan HJ, Blankenship DD, Voigt DE (2008) Seismic detection of a subglacial lake near the South Pole, Antarctica. Geophys Res Lett 35:L23501. 10.1029/2008GL035704 10.1029/2008GL035704

[CR229] Peters ST, Schroeder DM, Romero-Wolf A (2020) Passive radio sounding to correct for Europa’s ionospheric distortion of VHF signals. Planet Space Sci 187:104925. 10.1016/j.pss.2020.104925 10.1016/j.pss.2020.104925

[CR230] Peters ST, Roberts TM, Nessly K, Schroeder DM, Romero-Wolf A (2022) Revisiting the limits of spatial coherence for passive radar sounding using radio-astronomical sources. In: IGARSS 2022 - 2022 IEEE international geoscience and remote sensing symposium, pp 3880–3883. 10.1109/IGARSS46834.2022.9884673

[CR231] Pettinelli E, Cosciotti B, Di Paolo F, Lauro SE, Mattei E, Orosei R, Vannaroni G (2015) Dielectric properties of Jovian satellite ice analogs for subsurface radar exploration: a review. Rev Geophys 53:593–641. 10.1002/2014RG000463 10.1002/2014RG000463

[CR232] Pettinelli E, Lauro SE, Cosciotti B, Mattei E, Di Paolo F, Vannaroni G (2016) Dielectric characterization of ice/MgSO4⋅11H2O mixtures as Jovian icy moon crust analogues. Earth Planet Sci Lett 439:11–17. 10.1016/j.epsl.2016.01.021 10.1016/j.epsl.2016.01.021

[CR233] Phillips R, Adams G, Brown W Jr, Eggleton R, Jackson P, Jordan R, Peeples W, Porcello L, Ryu J, Schaber G (1973) Preliminary results of the Apollo lunar sounder experiment

[CR234] Phillips RJ, Davis BJ, Tanaka KL, Byrne S, Mellon MT, Putzig NE, Haberle RM, Kahre MA, Campbell BA, Carter LM (2011) Massive CO2 ice deposits sequestered in the south polar layered deposits of Mars. Science 332:838–841. 10.1126/science.1203091 21512003 10.1126/science.1203091

[CR235] Phillips CB et al A Reconnaissance Strategy for Landing on Europa, based on Europa Clipper Data. Submitted to Planet Sci J

[CR236] Picardi G, Biccari D, Seu R, Plaut J, Johnson W, Jordan R, Safaeinili A, Gurnett D, Huff R, Orosei R (2004) MARSIS: Mars advanced radar for subsurface and ionosphere sounding. In: Mars Express: the scientific payload. pp 51–69

[CR237] Picardi G, Plaut JJ, Biccari D, Bombaci O, Calabrese D, Cartacci M, Cicchetti A, Clifford SM, Edenhofer P, Farrell WM (2005) Radar soundings of the subsurface of Mars. Science 310:1925–1928. 10.1126/science.1122165 16319122 10.1126/science.1122165

[CR238] Plaut JJ, Picardi G, Safaeinili A, Ivanov AB, Milkovich SM, Cicchetti A, Kofman W, Mouginot J, Farrell WM, Phillips RJ, Clifford SM, Frigeri A, Orosei R, Federico C, Williams IP, Gurnett DA, Nielsen E, Hagfors T, Heggy E, Stofan ER, Plettemeier D, Watters TR, Leuschen CJ, Edenhofer P (2007) Subsurface radar sounding of the south polar layered deposits of Mars. Science 316:92–95. 10.1126/science.1139672 17363628 10.1126/science.1139672

[CR239] Porcello LJ, Jordan RL, Zelenka JS, Adams GF, Phillips RJ, Brown WE, Ward SH, Jackson PL (1974) The Apollo lunar sounder radar system. Proc IEEE 62:769–783. 10.1109/PROC.1974.9517 10.1109/PROC.1974.9517

[CR240] Priscu JC, Kalin J, Winans J, Campbell T, Siegfried MR, Skidmore M, Dore JE, Leventer A, Harwood DM, Duling D, Zook R, Burnett J, Gibson D, Krula E, Mironov A, McManis J, Roberts G, Rosenheim BE, Christner BC, Kasic K, Fricker HA, Lyons WB, Barker J, Bowling M, Collins B, Davis C, Gagnon A, Gardner C, Gustafson C, Kim O-S, Li W, Michaud A, Patterson MO, Tranter M, Venturelli R, Vick-Majors T, Elsworth C, TSS Team (2021) Scientific access into Mercer Subglacial Lake: scientific objectives, drilling operations and initial observations. Ann Glaciol 62:340–352. 10.1017/aog.2021.10 10.1017/aog.2021.10

[CR241] Prockter LM, Patterson GW (2009) Morphology and evolution of Europa’s ridges and bands. In: Pappalardo RT, McKinnon WB, Khurana KK (eds) Europa. University of Arizona Press, Tucson, pp 237–258. 10.2307/j.ctt1xp3wdw.16

[CR242] Prockter L, Schenk P (2005) Origin and evolution of Castalia Macula, an anomalous young depression on Europa. Icarus 177:305–326. 10.1016/j.icarus.2005.08.003 10.1016/j.icarus.2005.08.003

[CR243] Putzig NE, Phillips RJ, Campbell BA, Holt JW, Plaut JJ, Carter LM, Egan AF, Bernardini F, Safaeinili A, Seu R (2009) Subsurface structure of planum boreum from Mars reconnaissance orbiter shallow radar soundings. Icarus 204:443–457. 10.1016/j.icarus.2009.07.034 10.1016/j.icarus.2009.07.034

[CR244] Quick LC, Hedman MM (2020) Characterizing deposits emplaced by cryovolcanic plumes on Europa. Icarus 343:113667. 10.1016/j.icarus.2020.113667 10.1016/j.icarus.2020.113667

[CR245] Raju G, Xin W, Moore R (1990) Design, development, field observations, and preliminary results of the Coherent Antarctic Radar Depth Sounder (CARDS) of the University of Kansas, USA. J Glaciol 36:247–254. 10.3189/S0022143000009503 10.3189/S0022143000009503

[CR246] Reid HAS, Kontar EP (2021) Fine structure of type III solar radio bursts from Langmuir wave motion in turbulent plasma. Nat Astron 5:796–804. 10.1038/s41550-021-01370-8 10.1038/s41550-021-01370-8

[CR247] Rémy F, Parouty S (2009) Antarctic ice sheet and radar altimetry: a review. Remote Sens 1:1212–1239. 10.3390/rs1041212 10.3390/rs1041212

[CR248] Rippin DM, Bingham RG, Jordan TA, Wright AP, Ross N, Corr HFJ, Ferraccioli F, Le Brocq AM, Rose KC, Siegert MJ (2014) Basal roughness of the Institute and Möller Ice Streams, West Antarctica: process determination and landscape interpretation. Geomorphology 214:139–147. 10.1016/j.geomorph.2014.01.021 10.1016/j.geomorph.2014.01.021

[CR249] Robin GdQ, Doake C, Kohnen H, Crabtree R, Jordan S, Möller D (1983) Regime of the Filchner–Ronne ice shelves, Antarctica. Nature 302:582–586. 10.1038/302582a0 10.1038/302582a0

[CR250] Romero-Wolf A, Vance S, Maiwald F, Heggy E, Ries P, Liewer K (2015) A passive probe for subsurface oceans and liquid water in Jupiter’s icy moons. Icarus 248:463–477. 10.1016/j.icarus.2014.10.043 10.1016/j.icarus.2014.10.043

[CR251] Roth L, Saur J, Retherford KD, Strobel DF, Feldman PD, McGrath MA, Nimmo F (2014) Transient water vapor at Europa’s south pole. Science 343:171–174. 10.1126/science.1247051 24336567 10.1126/science.1247051

[CR252] Rutishauser A, Grima C, Sharp M, Blankenship DD, Young DA, Cawkwell F, Dowdeswell JA (2016) Characterizing near-surface firn using the scattered signal component of the glacier surface return from airborne radio-echo sounding. Geophys Res Lett 43:12–502. 10.1002/2016GL071230 10.1002/2016GL071230

[CR253] Rutishauser A, Blankenship DD, Sharp M, Skidmore ML, Greenbaum JS, Grima C, Schroeder DM, Dowdeswell JA, Young DA (2018) Discovery of a hypersaline subglacial lake complex beneath Devon Ice Cap, Canadian Arctic. Sci Adv 4:eaar4353. 10.1126/sciadv.aar4353 29651462 10.1126/sciadv.aar4353 PMC5895444

[CR254] Rutishauser A, Blankenship DD, Young DA, Wolfenbarger NS, Beem LH, Skidmore ML, Dubnick A, Criscitiello AS (2022) Radar sounding survey over Devon Ice Cap indicates the potential for a diverse hypersaline subglacial hydrological environment. Cryosphere 16:379–395. 10.5194/tc-16-379-2022 10.5194/tc-16-379-2022

[CR255] Safaeinili A, Kofman W, Nouvel J, Herique A, Jordan R (2003) Impact of Mars ionosphere on orbital radar sounder operation and data processing. Planet Space Sci 51:505–515. 10.1016/S0032-0633(03)00048-5 10.1016/S0032-0633(03)00048-5

[CR256] Safaeinili A, Kofman W, Mouginot J, Gim Y, Herique A, Ivanov AB, Plaut JJ, Picardi G (2007) Estimation of the total electron content of the Martian ionosphere using radar sounder surface echoes. Geophys Res Lett 34:L23204. 10.1029/2007GL032154 10.1029/2007GL032154

[CR257] Sasaki S, Iijima Y, Tanaka K, Kato M, Hashimoto M, Mizutani H, Takizawa Y (2003) The SELENE mission: goals and status. Adv Space Res 31:2335–2340. 10.1016/S0273-1177(03)00543-X 10.1016/S0273-1177(03)00543-X

[CR258] Saur J, Strobel D, Neubauer F (1998) Interaction of the Jovian magnetosphere with Europa: constraints on the neutral atmosphere. J Geophys Res, Planets 103:19947–19962. 10.1029/97JE03556 10.1029/97JE03556

[CR259] Scanlan KM, Grima C, Steinbrügge G, Kempf SD, Young DA, Blankenship DD (2019) Geometric determination of ionospheric total electron content from dual frequency radar sounding measurements. Planet Space Sci 178:104696. 10.1016/j.pss.2019.07.010 10.1016/j.pss.2019.07.010

[CR260] Scanlan KM, Rutishauser A, Young DA, Blankenship DD (2020) Interferometric discrimination of cross-track bed clutter in ice-penetrating radar sounding data. Ann Glaciol 61:68–73. 10.1017/aog.2020.20 10.1017/aog.2020.20

[CR261] Scanlan KM, Young DA, Steinbrügge G, Kempf SD, Grima C, Blankenship DD (2021) Delay Doppler SAR focusing and quantitative quality control of the radar for Europa assessment and sounding: ocean to near-surface (REASON) sounding data product. IEEE J Sel Top Appl Earth Obs Remote Sens 14:4352–4369. 10.1109/JSTARS.2021.3072276 10.1109/JSTARS.2021.3072276

[CR262] Scanlan KM, Young DA, Blankenship DD (2022) Non-linear radar response to the radial structure of Europa plume fallout deposits. Icarus 378:114935. 10.1016/j.icarus.2022.114935 10.1016/j.icarus.2022.114935

[CR263] Schenk PM (2002) Thickness constraints on the icy shells of the Galilean satellites from a comparison of crater shapes. Nature 417:419–421. 10.1038/417419a 12024207 10.1038/417419a

[CR264] Schenk PM, McKinnon WB (1991) Dark-ray and dark-floor craters on Ganymede, and the provenance of large impactors in the Jovian system. Icarus 89:318–346. 10.1016/0019-1035(91)90181-R 10.1016/0019-1035(91)90181-R

[CR265] Schlegel R, Kulessa B, Murray T, Eisen O (2022) Towards a common terminology in radioglaciology. Ann Glaciol 63:8–12. 10.1017/aog.2023.2 10.1017/aog.2023.2

[CR266] Schmidt BE, Blankenship DD, Patterson GW, Schenk PM (2011) Active formation of ‘chaos terrain’ over shallow subsurface water on Europa. Nature 479:502–505. 10.1038/nature10608 22089135 10.1038/nature10608

[CR267] Schroeder DM, Steinbrügge G (2021) Alternatives to liquid water beneath the south polar ice cap of Mars. Geophys Res Lett 48:e2021GL095912. 10.1029/2021GL095912 10.1029/2021GL095912

[CR268] Schroeder DM, Blankenship DD, Young DA (2013) Evidence for a water system transition beneath Thwaites Glacier, West Antarctica. Proc Natl Acad Sci 110:12225–12228. 10.1073/pnas.1302828110 23836631 10.1073/pnas.1302828110 PMC3725042

[CR269] Schroeder DM, Blankenship DD, Raney RK, Grima C (2014) Estimating subglacial water geometry using radar bed echo specularity: application to Thwaites Glacier, West Antarctica. IEEE Geosci Remote Sens Lett 12:443–447. 10.1109/LGRS.2014.2337878 10.1109/LGRS.2014.2337878

[CR270] Schroeder DM, Grima C, Blankenship DD (2015) Evidence for variable grounding-zone and shear-margin basal conditions across Thwaites Glacier, West Antarctica. Geophysics 81:WA35–WA43. 10.1190/geo2015-0122.1 10.1190/geo2015-0122.1

[CR271] Schroeder DM, Romero-Wolf A, Carrer L, Grima C, Campbell BA, Kofman W, Bruzzone L, Blankenship DD (2016) Assessing the potential for passive radio sounding of Europa and Ganymede with RIME and REASON. Planet Space Sci 134:52–60. 10.1016/j.pss.2016.10.007 10.1016/j.pss.2016.10.007

[CR272] Schroeder DM, Bingham RG, Blankenship DD, Christianson K, Eisen O, Flowers GE, Karlsson NB, Koutnik MR, Paden JD, Siegert MJ (2020) Five decades of radioglaciology. Ann Glaciol 61:1–13. 10.1017/aog.2020.11 10.1017/aog.2020.11

[CR273] Seu R, Biccari D, Orosei R, Lorenzoni LV, Phillips RJ, Marinangeli L, Picardi G, Masdea A, Zampolini E (2004) SHARAD: the MRO 2005 shallow radar. Planet Space Sci 52:157–166. 10.1016/j.pss.2003.08.024 10.1016/j.pss.2003.08.024

[CR274] Seu R, Phillips RJ, Biccari D, Orosei R, Masdea A, Picardi G, Safaeinili A, Campbell BA, Plaut JJ, Marinangeli L, Smrekar SE, Nunes DC (2007) SHARAD sounding radar on the Mars Reconnaissance Orbiter. J Geophys Res 112:E05S05. 10.1029/2006JE002745 10.1029/2006JE002745

[CR275] Shepard MK, Campbell BA (1999) Radar scattering from a self-affine fractal surface: near-nadir regime. Icarus 141:156–171. 10.1006/icar.1999.6141 10.1006/icar.1999.6141

[CR276] Siegfried MR, Fricker HA (2021) Illuminating active subglacial lake processes with ICESat-2 laser altimetry. Geophys Res Lett 48:e2020GL091089. 10.1029/2020GL091089 10.1029/2020GL091089

[CR277] Sihvola AH (1999) Electromagnetic mixing formulas and applications. The Insitution of Engineering and Technology (IET)

[CR278] Sihvola A (2013) Homogenization principles and effect of mixing on dielectric behavior. Photonics Nanostruct Fundam Appl 11:364–373. 10.1016/j.photonics.2013.01.004 10.1016/j.photonics.2013.01.004

[CR279] Singer KN, McKinnon WB, Schenk PM (2021) Pits, uplifts and small chaos features on Europa: morphologic and morphometric evidence for intrusive upwelling and lower limits to ice shell thickness. Icarus 364:114465. 10.1016/j.icarus.2021.114465 10.1016/j.icarus.2021.114465

[CR280] Singer KN, McKinnon WB, Schenk PM (2023) Thin ice lithospheres and high heat flows on Europa from large impact structure ring-graben. J Geophys Res, Planets 128:e2023JE007928. 10.1029/2023JE007928 10.1029/2023JE007928

[CR281] Smith IB, Putzig NE, Holt JW, Phillips RJ (2016) An ice age recorded in the polar deposits of Mars. Science 352:1075–1078. 10.1126/science.aad6968 27230372 10.1126/science.aad6968

[CR282] Smith IB, Lalich DE, Rezza C, Horgan BHN, Whitten JL, Nerozzi S, Holt JW (2021) A solid interpretation of bright radar reflectors under the Mars south polar ice. Geophys Res Lett 48:e2021GL093618. 10.1029/2021GL093618 10.1029/2021GL093618

[CR283] Sotin C, Head III JW Tobie G (2002) Europa: tidal heating of upwelling thermal plumes and the origin of lenticulae and chaos melting. Geophys Res Lett 29:74-1. 10.1029/2001GL013844 10.1029/2001GL013844

[CR284] Squyres SW, Reynolds RT, Cassen PM, Peale SJ (1983) Liquid water and active resurfacing on Europa. Nature 301:225–226. 10.1038/301225a0 10.1038/301225a0

[CR285] Steenson BO (1951) Radar methods for the exploration of glaciers (PhD thesis). California Institute of Technology

[CR286] Steinbrügge G, Schroeder DM, Haynes MS, Hussmann H, Grima C, Blankenship DD (2018) Assessing the potential for measuring Europa’s tidal Love number h_2_ using radar sounder and topographic imager data. Earth Planet Sci Lett 482:334–341. 10.1016/j.epsl.2017.11.028 10.1016/j.epsl.2017.11.028

[CR287] Steinbrügge G, Voigt JRC, Schroeder DM, Stark A, Haynes MS, Scanlan KM, Hamilton CW, Young DA, Hussmann H, Grima C, Blankenship DD (2020b) The surface roughness of Europa derived from Galileo stereo images. Icarus 343:113669. 10.1016/j.icarus.2020.113669 10.1016/j.icarus.2020.113669

[CR288] Steinbrügge G, Voigt JR, Wolfenbarger NS, Hamilton C, Soderlund K, Young D, Blankenship DD, Vance SD, Schroeder DM (2020a) Brine migration and impact-induced cryovolcanism on Europa. Geophys Res Lett 47:e2020GL090797. 10.1029/2020GL090797 10.1029/2020GL090797

[CR289] Steinbrügge G, Haynes MS, Schroeder DM, Scanlan KM, Stark A, Young DA, Grima C, Kempf S, Ng G, Buhl D (2021) Altimetry measurements from planetary radar sounders and application to SHARAD on Mars. IEEE Trans Geosci Remote Sens 60:1–14. 10.1109/TGRS.2021.3134638 10.1109/TGRS.2021.3134638

[CR290] Stern W (1930) Principles, methods and results of electrodynamic thickness measurement of glacier ice. Z Gletscherkunde 18:24

[CR291] Stillman D, Grimm R, MacGregor J (2018) Chloride Salts Prevent Direct Determination of Europa’s Icy Shell Thickness via Radar Sounding. In: 49th Lunar and Planetary Science Conference, LPI Contribution No. 2083, pp 1971

[CR292] Stillman DE, Pettinelli E, Lauro SE, Mattei E, Caprarelli G, Cosciotti B, Primm KM, Orosei R (2022) Partially-saturated brines within basal ice or sediments can explain the bright basal reflections in the south polar layered deposits. J Geophys Res, Planets 127:e2022JE007398. 10.1029/2022JE007398 10.1029/2022JE007398

[CR293] Talalay P (2012) Russian researchers reach subglacial Lake Vostok in Antarctica. Adv Polar Sci 23:176–180

[CR294] Thomas P, Tajeddine R, Tiscareno M, Burns J, Joseph J, Loredo T, Helfenstein P, Porco C (2016) Enceladus’s measured physical libration requires a global subsurface ocean. Icarus 264:37–47. 10.1016/j.icarus.2015.08.037 10.1016/j.icarus.2015.08.037

[CR295] Thomas EC, Hodyss R, Vu TH, Johnson PV, Choukroun M (2017) Composition and evolution of frozen chloride brines under the surface conditions of Europa. ACS Earth Space Chem 1:14–23. 10.1021/acsearthspacechem.6b00003 10.1021/acsearthspacechem.6b00003

[CR296] Thyssen F (1988) Special aspects of the central part of Filchner-Ronne Ice Shelf, Antarctica. Ann Glaciol 11:173–179. 10.3189/S0260305500006509 10.3189/S0260305500006509

[CR297] Tomlinson TC, Hayne PO (2022) Composition and possible origins of dark crater ejecta on Europa. Icarus 385:115037. 10.1016/j.icarus.2022.115037 10.1016/j.icarus.2022.115037

[CR298] Trumbo SK, Brown ME, Hand KP (2019) Sodium chloride on the surface of Europa. Sci Adv 5:eaaw7123. 10.1126/sciadv.aaw7123 31206026 10.1126/sciadv.aaw7123 PMC6561749

[CR299] Trumbo SK, Brown ME, Hand KP (2020) Endogenic and exogenic contributions to visible-wavelength spectra of Europa’s trailing hemisphere. Astron J 160:282. 10.3847/1538-3881/abc34c 10.3847/1538-3881/abc34c

[CR300] Trumbo SK, Becker TM, Brown ME, Denman WT, Molyneux P, Hendrix A, Retherford KD, Roth L, Alday J (2022) A new UV spectral feature on Europa: confirmation of NaCl in leading-hemisphere chaos terrain. Planet Sci J 3:27. 10.3847/PSJ/ac4580 10.3847/PSJ/ac4580

[CR301] Tulaczyk S, Mikucki JA, Siegfried MR, Priscu JC, Barcheck CG, Beem LH, Behar A, Burnett J, Christner BC, Fisher AT, Fricker HA, Mankoff KD, Powell RD, Rack F, Sampson D, Scherer RP, Schwartz SY, TWS Team (2014) WISSARD at Subglacial Lake Whillans, West Antarctica: scientific operations and initial observations. Ann Glaciol 55:51–58. 10.3189/2014AoG65A009 10.3189/2014AoG65A009

[CR302] Turchetti S, Dean K, Naylor S, Siegert M (2008) Accidents and opportunities: a history of the radio echo-sounding of Antarctica, 1958–79. Br J Hist Sci 41:417–444. 10.1017/S0007087408000903 10.1017/S0007087408000903

[CR303] Turtle EP, McEwen AS, Patterson GW et al (2024) The Europa Imaging System (EIS) investigation. Space Sci Rev 220 10.1007/s11214-024-01115-9 PMC11618168 39650165

[CR304] Ulaby F, Long D (2015) Microwave radar and radiometric remote sensing. Artech House

[CR305] Vance SD, Craft KL, Shock E et al. (2023) Investigating Europa’s habitability with the Europa Clipper. Space Sci Rev 219:81. 10.1007/s11214-023-01025-2 38046182 10.1007/s11214-023-01025-2 PMC10687213

[CR306] Vaughan D, Sievers J, Doake C, Hinze H, Mantripp D, Pozdeev V, Sandhäger H, Schenke H-W, Solheim A, Thyssen F (1995) Subglacial and seabed topography, ice thickness and water columm thickness in the vicinity of Filchner-Ronne-Schelfseis, Antarctica. Polarforschung 64:75–88

[CR307] Vaughan DG, Rivera A, Woodward J, Corr HFJ, Wendt J, Zamora R (2007) Topographic and hydrological controls on Subglacial Lake Ellsworth, West Antarctica. Geophys Res Lett 34:L18501 10.1029/2007GL030769 10.1029/2007GL030769

[CR308] Vilella K, Choblet G, Tsao W, Deschamps F (2020) Tidally heated convection and the occurrence of melting in icy satellites: application to Europa. J Geophys Res, Planets 125:e2019JE006248. 10.1029/2019JE006248 10.1029/2019JE006248

[CR309] Vu TH, Hodyss R, Choukroun M, Johnson PV (2016) Chemistry of frozen sodium–magnesium–sulfate–chloride brines: implications for surface expression of Europa’s ocean composition. Astrophys J Lett 816:L26. 10.3847/2041-8205/816/2/L26 10.3847/2041-8205/816/2/L26

[CR310] Wahr J, Zuber M, Smith D, Lunine J (2006) Tides on Europa, and the thickness of Europa’s icy shell. J Geophys Res, Planets 111:E12005. 10.1029/2006JE002729 10.1029/2006JE002729

[CR311] Waite AH, Schmidt SJ (1962) Gross errors in height indication from pulsed radar altimeters operating over thick ice or snow. Proc IRE 50:1515–1520. 10.1109/JRPROC.1962.288195. 10.1109/JRPROC.1962.288195

[CR312] Walker ME, Rhoden AR (2022) Tidal heating at Europa using the multifrequency analysis of tidal heating toolkit. Planet Sci J 3:149. 10.3847/PSJ/ac6df0 10.3847/PSJ/ac6df0

[CR313] Warren SG (1984) Optical constants of ice from the ultraviolet to the microwave. Appl Opt 23:1206–1225. 10.1029/2007JD009744 18204705 10.1029/2007JD009744

[CR314] Weertman J (1974) Stability of the junction of an ice sheet and an ice shelf. J Glaciol 13:3–11. 10.3189/S0022143000023327 10.3189/S0022143000023327

[CR315] Westlake JH, McNutt RL, Grey M et al. (2023) The Plasma Instrument for Magnetic Sounding (PIMS) on the Europa Clipper Mission. Space Sci Rev 291:62. 10.1007/s11214-023-01002-9 10.1007/s11214-023-01002-9

[CR316] Wingham DJ, Siegert MJ, Shepherd A, Muir AS (2006) Rapid discharge connects Antarctic subglacial lakes. Nature 440:1033–1036. 10.1038/nature04660 16625193 10.1038/nature04660

[CR317] Wolfenbarger NS, Buffo JJ, Soderlund KM Blankenship DD (2022a) Ice shell structure and composition of ocean worlds: insights from accreted ice on Earth. Astrobiology 22:937–961. 10.1089/ast.2021.0044 35787145 10.1089/ast.2021.0044

[CR318] Wolfenbarger NS, Fox-Powell MG, Buffo J, Soderlund KM, Blankenship DD (2022b) Compositional controls on the distribution of brine in Europa’s ice shell. J Geophys Res, Planets 127:e2022JE007305. 10.1029/2022JE007305 10.1029/2022JE007305

[CR319] Wolfenbarger NS, Fox-Powell MG, Buffo J, Soderlund KM, Blankenship DD (2022c) Brine Volume Fraction as a Habitability Metric for Europa’s ice shell. Geophys Res Lett 49:e2022GL100586. 10.1029/2022GL100586 10.1029/2022GL100586

[CR320] Wolfenbarger NS, Scanlan KM, Findlay D, Young DA, Schroeder DM, Blankenship DD (2023) Detecting Fossil Brines within Europa’s Ice Shell Using Ice-Penetrating Radar. In: Brines Across the Solar System: Ancient and Future Brines, LPI Contribution No. 2689, pp 2016

[CR321] Woodward J, Smith AM, Ross N, Thoma M, Corr HFJ, King EC, King MA, Grosfeld K, Tranter M, Siegert MJ (2010) Location for direct access to subglacial Lake Ellsworth: an assessment of geophysical data and modeling. Geophys Res Lett 37:L11501. 10.1029/2010GL042884 10.1029/2010GL042884

[CR322] Zarka P, Cecconi B, Kurth WS (2004) Jupiter’s low-frequency radio spectrum from Cassini/Radio and Plasma Wave Science (RPWS) absolute flux density measurements. J Geophys Res Space Phys 109:A09S151. 10.1029/2003JA010260 10.1029/2003JA010260

[CR323] Zolotov MY, Kargel JS (2009) On the chemical composition of Europa’s icy shell, ocean, and underlying rocks. In: Pappalardo RT, McKinnon WB, Khurana KK (eds) Europa. University of Arizona Press, Tucson, pp 431–457. 10.2307/j.ctt1xp3wdw.18

[CR324] Zolotov MY, Shock EL (2001) Composition and stability of salts on the surface of Europa and their oceanic origin. J Geophys Res, Planets 106:32815–32827. 10.1029/2000JE001413 10.1029/2000JE001413

[CR325] Zolotov MY, Shock EL (2004) A model for low-temperature biogeochemistry of sulfur, carbon, and iron on Europa. J Geophys Res, Planets 109:E06003. 10.1029/2003JE002194 10.1029/2003JE002194

